# Nickel-catalyzed arylation of heteroaryl-containing diarylmethanes: exceptional reactivity of the Ni(NIXANTPHOS)-based catalyst†
†Electronic supplementary information (ESI) available: Data for new compounds, experimental procedures, ^1^H and ^13^C NMR spectra. See DOI: 10.1039/c5sc03704b


**DOI:** 10.1039/c5sc03704b

**Published:** 2015-10-26

**Authors:** Xinyu Cao, Sheng-Chun Sha, Minyan Li, Byeong-Seon Kim, Catherine Morgan, Rudan Huang, Xiaodong Yang, Patrick J. Walsh

**Affiliations:** a Key Laboratory of Cluster Science of Ministry of Education , School of Chemistry , Beijing Institute of Technology , Beijing 100081 , PR China; b Department of Chemistry , University of Pennsylvania , 231 S. 34th St. , Philadelphia , PA 19104 , USA . Email: pwalsh@sas.upenn.edu ; https://sites.google.com/site/titaniumupenn/ ; Fax: +1-215-573-6743; c Key Laboratory of Medicinal Chemistry for Natural Resource , School of Chemical Science and Technology , Yunnan University , Kunming , 650091 , PR China

## Abstract

Nickel(0)-catalyzed cross-coupling of heteroaryl-containing diarylmethanes with both aryl bromides and chlorides has been achieved. The success of this reaction relies on the introduction of a unique nickel/NIXANTPHOS-based catalyst system, which provides a direct route to triarylmethanes from heteroaryl-containing diarylmethanes. Reactivity studies indicate the Ni(NIXANTPHOS)-based catalyst exhibits enhanced reactivity over XANTPHOS derivatives and other Ni(phosphine)-based catalysts in the reactions examined.

## Abstract 2

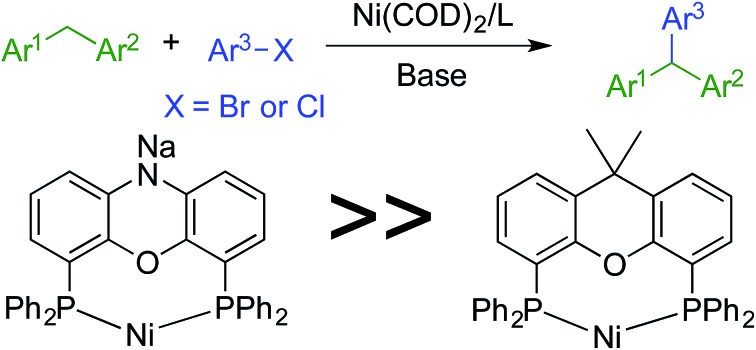
Nickel(0)-catalyzed cross-coupling of heteroaryl-containing diarylmethanes with both aryl bromides and chlorides has been achieved.

## 1 Introduction

Triarylmethanes have attracted significant attention due to their diverse applications as leuco dye precursors,^1^ fluorescent probes,^2^ and photochromic agents.^3^ They also find uses in medicinal chemistry.^4^ Given the utility of triarylmethanes, it is not surprising that considerable effort has been invested in their synthesis.^5^,^6^ Recently, triarylmethanes have been prepared using transition metal catalyzed cross-coupling reactions, usually employing palladium-based catalysts.^7^ To decrease costs and increase sustainability of these processes, much effort has focused on employing earth abundant first row transition metals in reactions traditionally catalyzed by palladium.^8^–^11^ Given nickel's position directly above palladium, and its high reactivity with aryl halides,^12^ nickel stands out as a clear starting point for the development of first-row transition metal catalyzed processes. Indeed, it has been used successfully in many cross-coupling reactions.^12^–^14^

**Scheme 1:**
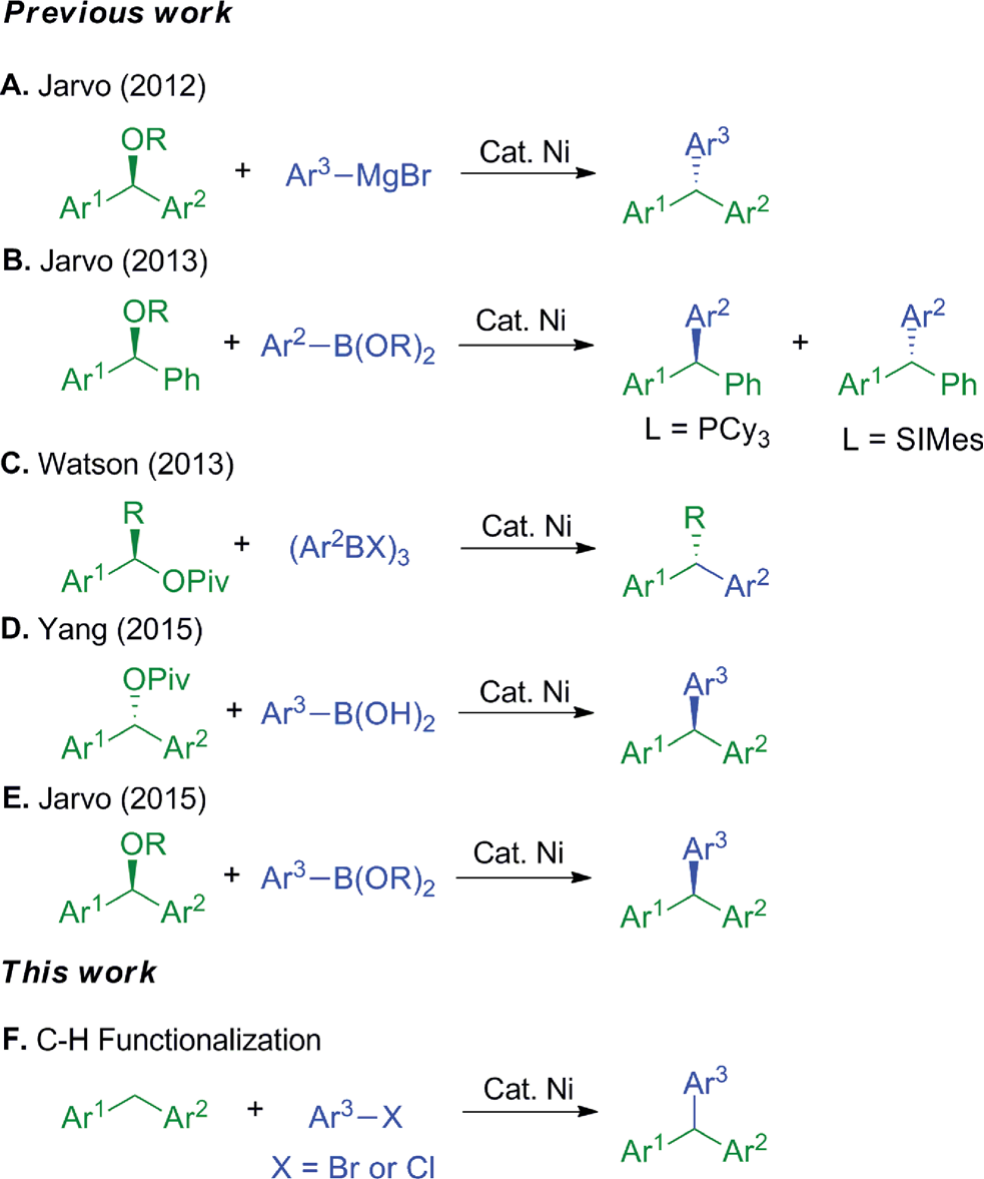
Synthetic approaches to triarylmethanes.

The synthesis of triarylmethanes *via* nickel-catalyzed reactions has been achieved through traditional Kumada and Suzuki–Miyaura coupling approaches.^8^–^11^ In 2012 and 2013, the Jarvo group demonstrated the synthesis of triarylmethanes with high ee by coupling of enantioenriched benzylic ethers with aryl Grignard reagents8*a* (Scheme 1A) and arylboronic esters (Scheme 1B).^9^ Related strategies were reported by the Watson^10^ and Yang^11^ groups with enantioenriched benzylic pivalates and aryl boronic acid derivatives. More recently, the Jarvo group reported Suzuki reaction as a complementary method to the previously reported nickel-catalyzed Kumada coupling.8*b*

Our approach to triarylmethanes differs from these important contributions in that it involves the catalytic functionalization of weakly acidic sp^3^-hybridized C–H bonds *via* a deprotonative-cross-coupling process (DCCP).^15^,^16^ In our prior studies, diarylmethanes were reversibly deprotonated in the presence of a Pd(NIXANTPHOS) catalyst and the resulting carbanions coupled with aryl bromides17a,c and chlorides17*b* in high yields. It is noteworthy that early work on the deprotonation/cross coupling of both aromatic C(sp^2^)–H's and C(sp^3^)–H's of fluorinated hydrocarbons were reported by Daugulis employing base metal catalysts (Cu and Fe).17d–g

One of the most significant findings of our early investigations is that under the basic reaction conditions, van Leeuwen's wide bite-angle ligand,^18^ NIXANTPHOS' N–H (p*K*_a_ ∼ 22)^19^ is deprotonated under the reaction conditions and the resulting heterobimetallic catalyst displays exceptional reactivity when compared with other bidentate phosphine-based palladium catalysts.17*b* The most striking comparison is between the NIXANTPHOS- and XANTPHOS-based palladium catalysts. Despite the outward similarity of these ligand scaffolds, the NIXANTPHOS-based catalyst exhibited over 90% assay yield (AY, determined by ^1^H NMR) in the coupling of 1-chloro-4-*tert*-butylbenzene with diphenylmethane to afford the triarylmethane product, while the parent XANTPHOS-based catalyst, under identical conditions, exhibited only 1% conversion (eqn (1)). Furthermore, other ligands known to participate in cross-coupling reactions with aryl chlorides, including the Buchwald family of ligands, were examined in this reaction and found to give less than 2% product. These results attest to the difficulty of this reaction, particularly with aryl chlorides.1
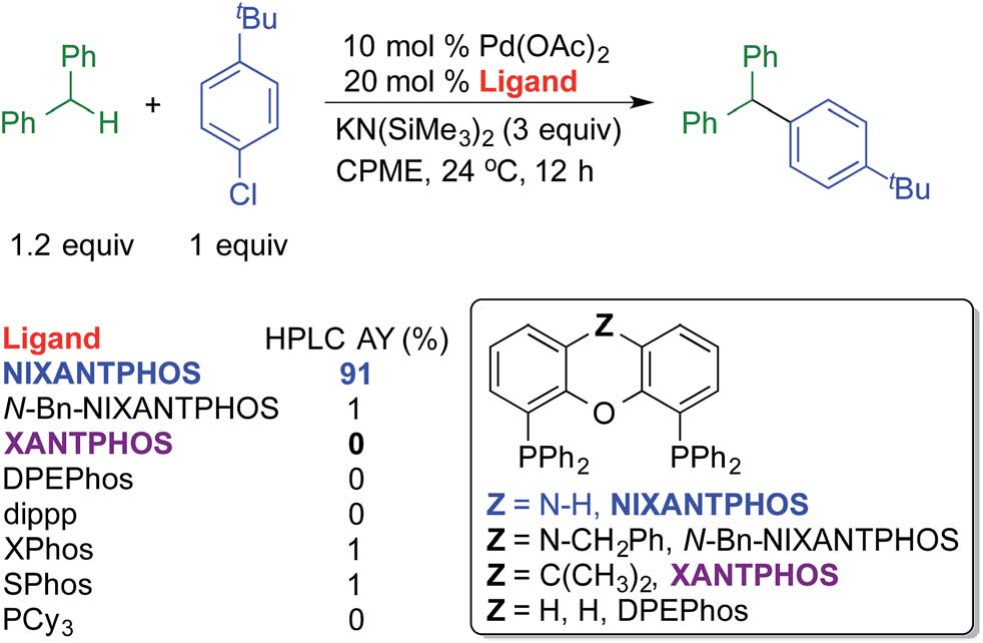



In the present study, our goals were to (1) develop a nickel catalyst to promote the DCCP of heteroaryl-containing diarylmethane derivatives and (2) determine if the high reactivity imparted to palladium when chelated by a deprotonated NIXANTPHOS ligand translates to nickel catalysts. Herein, we describe the synthesis of triarylmethanes by DCCP of heteroaryl-containing diarylmethanes and aryl bromides and chlorides catalyzed by a Ni(NIXANTPHOS)-based system. To determine if the exceptional reactivity of the Ni(NIXANTPHOS)-based catalyst translates to additional reactions, we also report preliminary results on the previously unknown arylation of a 2-pyridylmethyl amine. Compared with 37 mono- and bidentate phosphine ligands, the Ni(NIXANTPHOS)-based catalyst outperformed the other nickel catalysts in the arylation of 2-pyridylmethyl amine under the conditions examined.

## 2 Results and discussion

### 2.1 Catalyst identification

We evaluated nickel precursors in the presence of phosphine ligands by screening 37 electronically diverse mono- and bidentate phosphines using Ni(COD)_2_ as the nickel source. Coupling of 2-benzylpyridine **1a** (1.2 equiv.) with 1-bromo-4-*tert*-butylbenzene **2a** (1 equiv.) in the presence of NaN(SiMe_3_)_2_ (3 equiv.), catalytic Ni(COD)_2_ (10 mol%) and ligand (20 mol% for monodentate phosphine ligands and 10 mol% for bidentate ligands) were performed in cyclopentyl methyl ether (CPME) on microscale (0.01 mmol, 0.1 M) at 110 °C. After heating for 16 h, the reactions were worked up and analyzed by HPLC (retention times, UV/vis characteristics and integration against an internal standard, see ESI† for details). Of the 37 different phosphine ligands tested, NIXANTPHOS was *the only promising hit*. Interestingly, as discussed below, the XANTPHOS-based catalyst was much less active in this transformation. Translation of the microscale results with commercially available NIXANTPHOS to laboratory scale (0.1 mmol) under the same reaction conditions resulted in 78% assay yield (AY, as determined by ^1^H NMR in all cases) (Table 1, entry 1). Examination of different nickel to ligand ratios indicated that a 1 : 1 ratio at 10 mol% loading provided the best yields (entries 2–3). Decreasing the equivalents of NaN(SiMe_3_)_2_ from 3 to 2 resulted in an increase in the yield of coupling product **3aa** to 83% (entry 4). Reducing the concentration to 0.05 M resulted in generation of product **3aa** in 85% (entry 5).

**Table 1:** Optimization of Ni-NIXANTPHOS catalyzed DCCP of **1a** with **2a**^*a*^

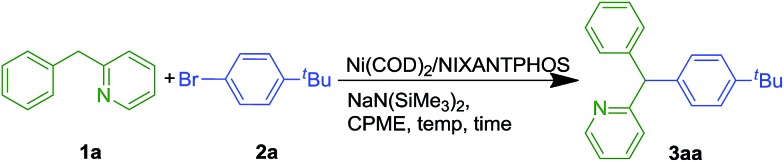						
Entry	**1a** : **2a** : base	Ni/L (mol%)	*T* (°C)	*t* (h)	Concn	Yield^*b*^ (%)
1	1.2 : 1 : 3	10/10	110	16	0.1	78
2	1.2 : 1 : 3	10/15	110	16	0.1	71
3	1.2 : 1 : 3	10/20	110	16	0.1	62
4	1.2 : 1 : 2	10/10	110	16	0.1	83
5	1.2 : 1 : 2	10/10	110	16	0.05	85
6	1 : 1.5 : 2	10/10	110	16	0.05	93
7	1 : 1.5 : 2	10/10	110	8	0.05	77
8	1 : 1.5 : 2	10/10	110	24	0.05	81
9	1 : 1.5 : 2	10/10	50	16	0.05	99
10	1 : 1.5 : 2	10/10	rt	16	0.05	99
11^*c*^	1 : 1.5 : 2	2.5/2.5	rt	16	0.05	99(99)
12	1 : 1.5 : 2	1/1	rt	16	0.05	72

^*a*^Reactions conducted on a 0.10 mmol scale.

^*b*^Yield determined by ^1^H NMR spectroscopy of the crude reaction mixtures.

^*c*^Isolated yield after chromatographic purification.

Optimization was continued by adjusting other reaction parameters. Changing the ratio of 2-benzylpyridine to aryl bromide and base to 1.0 : 1.5 : 2.0 rendered product **3aa** in 93% AY (entry 6). Changing the reaction times from 16 to 8 or 24 h led to lower yields (entries 7 and 8). Lowering the temperature from 110 °C to 50 °C and room temperature increased the AY to 99% at both the lower temperatures (entries 9 and 10). Decreasing the catalyst loading to 2.5 mol% at room temperature also resulted in 99% AY (entry 11). Further decreasing the catalyst loading to 1 mol% at rt led to 72% AY (entry 12). Under the optimized conditions of entry 11, the triarylmethane product was isolated in 99% yield. Substitution of XANTPHOS for NIXANTPHOS at twice the catalyst loading (5 mol%), under otherwise identical conditions afforded only 30% AY. This result prompted a series of experiments probing the difference in reactivity between these two structurally similar ligands (see later sections).

### 2.2 Scope of the diphenylmethane derivatives

**Scheme 2:**
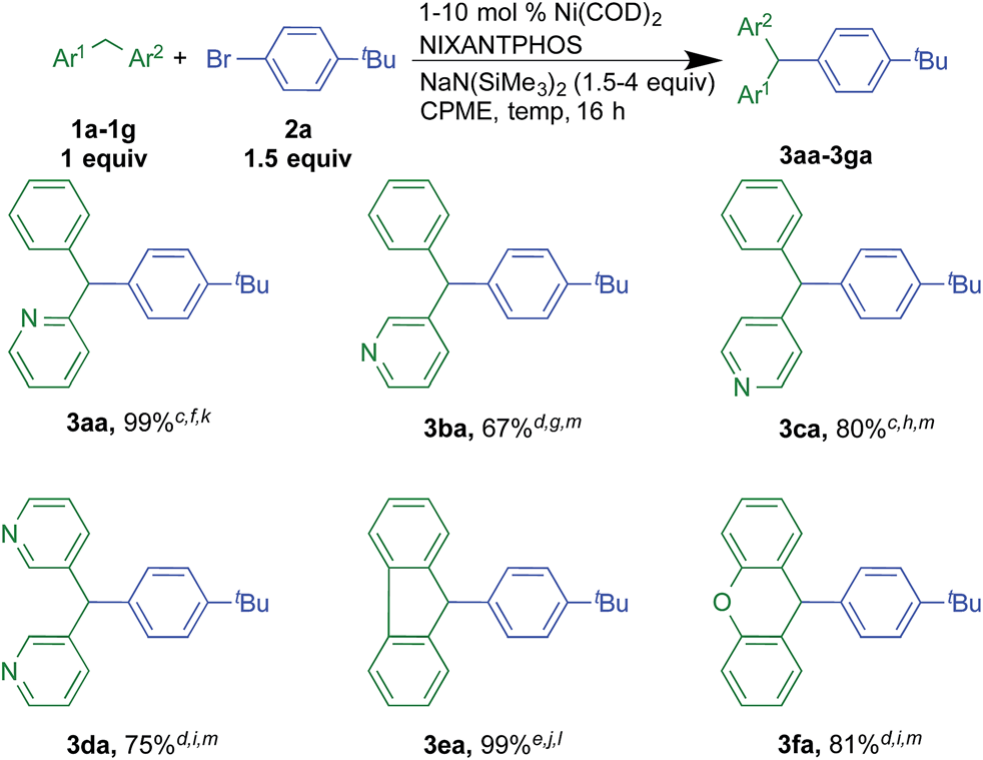
Scope of heteroaryl-containing diarylmethanes in Ni-NIXANTPHOS catalyzed DCCP^*a*,*b*^. ^*a*^ Reactions conducted on a 0.1 mmol scale at 0.05 M. ^*b*^ Isolated yields after chromatographic purification. ^*c*^ 2.5 mol% Ni(COD)_2_/2.5 mol% NIXANTPHOS. ^*d*^ 10 mol% Ni(COD)_2_/10 mol% NIXANTPHOS. ^*e*^ 1 mol% Ni(COD)_2_/1 mol% NIXANTPHOS. ^*f*^ 2 equiv. of NaN(SiMe_3_)_2_. ^*g*^ 4 equiv. of NaN(SiMe_3_)_2_. ^*h*^ 2.5 equiv. of LiN(SiMe_3_)_2_. ^*i*^ 2 equiv. of LiN(SiMe_3_)_2_. ^*j*^ 1.5 equiv. of NaN(SiMe_3_)_2_. ^*k*^ Room temperature. ^*l*^ 50 °C. ^*m*^ 110 °C.

Under the optimized conditions of entry 12 in Table 1, the scope of heteroaryl-containing diarylmethanes with 4-*tert*-butyl bromobenzene (**2a**) was examined (Scheme 2). The isomeric 2-, 3- and 4-benzylpyridine substrates have p*K*_a_ values of 28.2, 30.1, and 26.7 in DMSO, respectively.^19^ It is not entirely surprising that their reactions required different conditions to achieve high yields (Scheme 2, see ESI† for details). Nonetheless, coupling of 2-, 3- and 4-benzylpyridine furnished products **3aa**, **3ba** and **3ca** in 99, 67 and 96% isolated yields, respectively. 3-Benzylpyridine was the most challenging of the benzyl pyridines, because it is the most difficult to deprotonate. Nonetheless, the more acidic dipyridin-3-ylmethane (**1d**) underwent coupling with aryl bromide **2a** and 2 equiv. of LiN(SiMe_3_)_2_ in 75% isolated yield. Unlike analogous palladium-catalyzed reactions with less acidic diarylmethanes,^7^ such as diphenylmethane (p*K*_a_ = 32 in DMSO)^20^ the Ni(NIXANTPHOS)-based catalyst failed to give product with substrates with benzylic C–H p*K*_a_'s values greater than about 30. This result is not totally surprising. Based on the assumption that the reaction pathways are similar,^21^ we hypothesize that the decreased electronegativity of nickel (1.91) relative to palladium (2.20, Pauling scale) renders the Ni catalyst less electrophilic.^22^ This, combined with the smaller size of Ni compared to Pd^23^ and the small value of the equilibrium for deprotonation (p*K*_a_ of HN(SiMe_3_)_2_ in THF = 26),^24^ likely results in very little or no transmetallation with the anion derived from diphenylmethane.

In addition to benzylpyridines, other diphenylmethane derivatives underwent nickel-catalyzed coupling reactions. These include fluorene (**1e**) and xanthene (**1f**, p*K*_a_ in DMSO = 30.0).^19^ The triarylmethane derivatives **3ea** and **3fa** were isolated in 99 and 81% yield, respectively. Notably, reactions with the more acidic fluorene required only 1 mol% catalyst loading. Related triarylmethanes with these core structures have been found to have various applications.^25^,^26^

### 2.3 Scope of the aryl halide

**Scheme 3:**
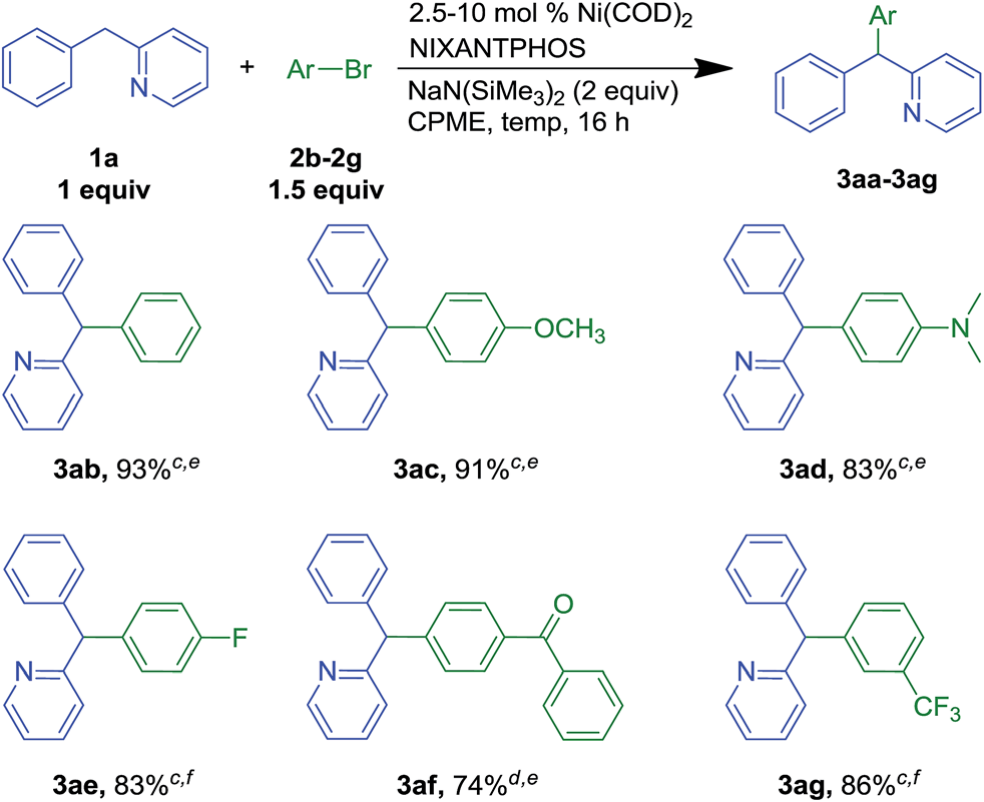
Scope of aryl bromides in Ni-NIXANTPHOS catalyzed DCCP with **1a**^*a*,*b*^. ^*a*^ Reactions conducted on a 0.1 mmol scale at 0.05 M. ^*b*^ Isolated yields after chromatographic purification. ^*c*^ 5 mol% Ni(COD)_2_/5 mol% NIXANTPHOS. ^*d*^ 10 mol% Ni(COD)_2_/10 mol% NIXANTPHOS. ^*e*^ Room temperature. ^*f*^ 50 °C.

To probe the ability of the Ni(NIXANTPHOS)-based catalyst to promote reactions with different electrophiles, 2-benzylpyridine was coupled with various aryl bromides (Scheme 3). Bromobenzene (**2b**) gave coupling product **3ab** in 93% yield with 5 mol% catalyst loading at room temperature. Under the same conditions, electron rich 4-bromoanisole (**2c**) and 4-bromo-*N*,*N*-dimethylaniline (**2d**) furnished products **3ac** and **3ad** in 91 and 83% yields, respectively. Aryl bromide with a 4-fluoro- group afforded triarylmethane **3ae** in 83% isolated yield, (5 mol% catalyst loading at 50 °C). Keto-containing 4-bromobenzophenone delivered **3af** in 74% yield with 10 mol% catalyst at room temperature. Aryl bromide bearing a 3-CF_3_ substituent furnished the product **3ag** in 86% yield with 5 mol% catalyst at 50 °C.

**Scheme 4:**
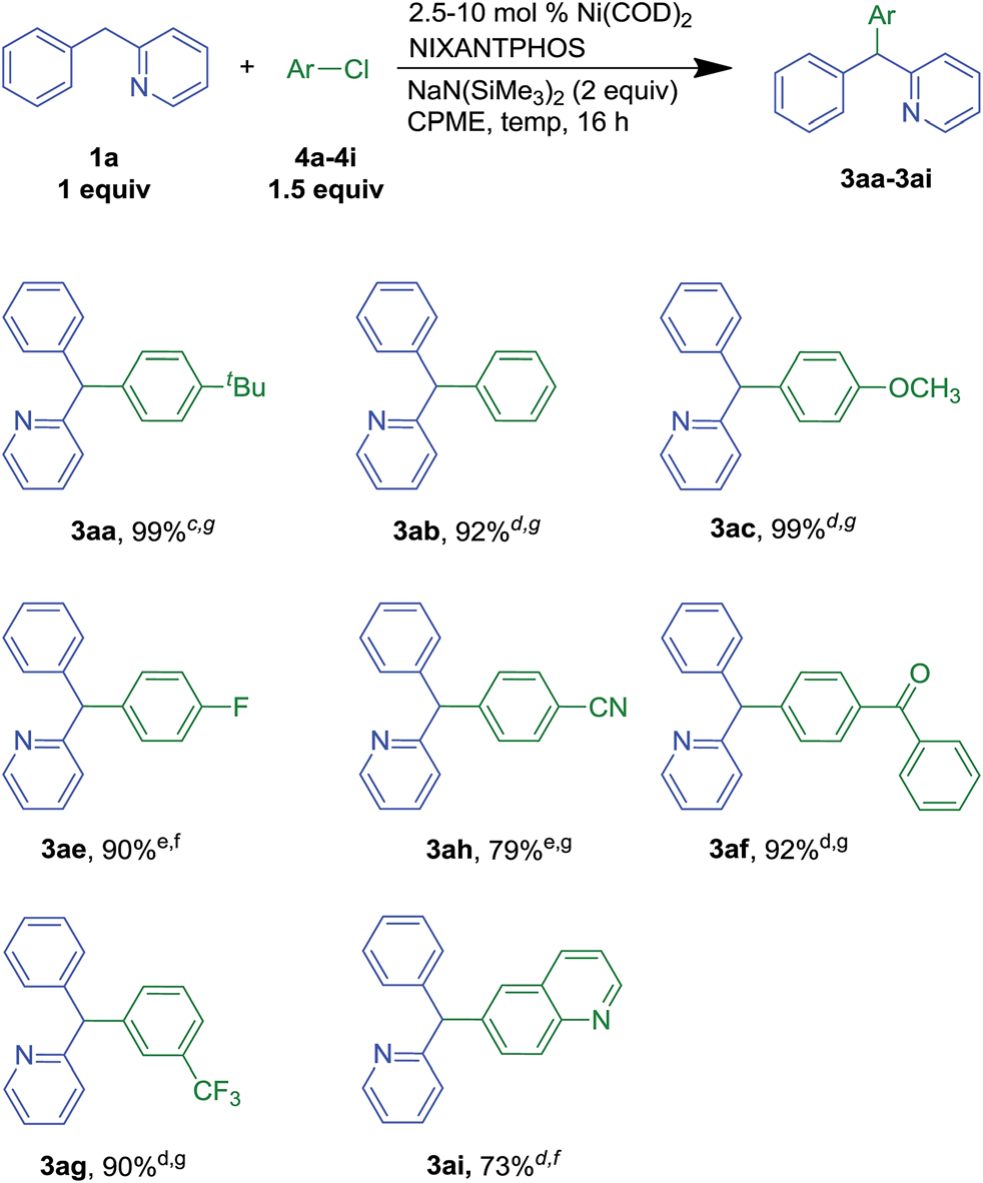
Scope of aryl chlorides in Ni-NIXANTPHOS catalyzed DCCP with **1a**^*a*,*b*^. ^*a*^ Reactions conducted on a 0.1 mmol scale at 0.05 M. ^*b*^ Isolated yield after chromatographic purification. ^*c*^ 2.5 mol% Ni(COD)_2_/2.5 mol% NIXANTPHOS. ^*d*^ 5 mol% Ni(COD)_2_/5 mol% NIXANTPHOS. ^*e*^ 10 mol% Ni(COD)_2_/10 mol% NIXANTPHOS. ^*f*^ room temperature. ^*g*^ 50 °C.

We next examined our Ni(NIXANTPHOS)-based catalyst with more challenging aryl chlorides (Scheme 4). We were pleased to find that the Ni(NIXANTPHOS)-based catalyst gave good to excellent yields in the coupling of aryl chlorides with 2-benzylpyridine using catalyst loadings as low as 2.5 mol%. Coupling chlorobenzene, 1-*tert*-butyl-4-chlorobenzene, and 4-chloroanisole furnished products in 99 (**3aa**), 92 (**3ab**) and 99% (**3ac**) yields with 2.5–5 mol% catalyst loadings at room temperature. 4-Chloro-1-fluorobenzene (**4e**) led to triarylmethane products **3ae** in 90% yield with 10 mol% catalyst loading at room temperature. 4-Chlorobenzonitrile (**4h**) delivered **3ah** in 79% yield with 10 mol% catalyst loading at 50 °C, despite the known reaction of nitriles with silyl amide bases.^27^ Chlorobenzophenone afforded **3af** in 92% yield (5 mol% catalyst loading at 50 °C) while 3-chlorobenzotrifluoride (**4g**) furnished **3ag** in 90% yield with 5 mol% catalyst loading at 50 °C. Heteroaromatic 6-chloroquinoline **4i** successfully underwent reaction in 73% yield with 5 mol% catalyst loading at room temperature. Overall, good scope of the nickel-catalyzed DCCP was demonstrated in Schemes 2–4, and aryl chlorides were found to be better substrates than aryl bromides (based on yields).

**Scheme 5:**
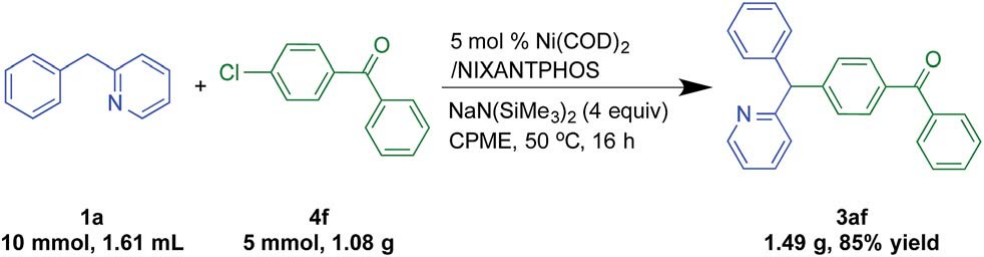
Gram scale reaction *via* nickel-catalyzed DCCP reaction.

To demonstrate the potential utility of our method, we conducted a gram scale reaction of chlorobenzophenone (**4f**, 5 mmol, 1.08 g) and the 2-benzylpyridine (**1a**, 10 mmol, 1.61 mL) in 85% yield (Scheme 5), suggesting the reaction is scalable.

### 2.4 Comparison of NIXANTPHOS and XANTPHOS derivatives

We previously demonstrated that oxidative addition of aryl chlorides to a Pd^0^(NIXANTPHOS) catalyst took place at room temperature in the presence of base.17*b* This result was surprising because the low energy pathway to oxidative addition of unactivated aryl chlorides to Pd(0) has been shown to proceed through a Pd–L intermediate, where L is a monodentate phosphine.^28^ Palladium complexes of bulky trialkyl phosphines and certain Buchwald ligands will oxidatively add aryl chlorides at room temperature or below.^29^ In contrast, Pd(0) complexes of bidentate phosphines can oxidatively add aryl chlorides *via* a higher energy PdL_2_ pathway.^30^ Such reactions usually take place around 90 °C, rather than at room temperature with our Pd^0^(NIXANTPHOS)-based catalyst.^31^ We also demonstrated that the NIXANTPHOS N–H must be deprotonated to perform the oxidative addition of aryl chlorides at room temperature, and that the actual catalyst is a heterobimetallic Pd/main group metal complex (main group = K, Na, Li). Although the precise mechanism of the oxidative addition in this system is currently unknown, we speculate that cooperativity between the metals plays an important role. As a point of reference, the structurally related Pd^0^(XANTPHOS) exhibited no reactivity with aryl chlorides under the same reaction conditions.17*b*

With this backdrop, we were interested to determine if the exceptional reactivity imparted to palladium when bound to NIXANTPHOS would translate to an increase in reactivity of analogous nickel complexes. Nickel-based phosphine catalysts are more reactive toward oxidative addition than most palladium complexes.^12^,^32^ Unfortunately, the nickel-based catalysts proved more challenging to study than their palladium counterparts, due to precipitate formation. Nonetheless, some experiments are presented that highlight the significant differences in reactivity between the nickel NIXANTPHOS and XANTPHOS systems.

As discussed earlier, our initial ligand screen indicated that the NIXANTPHOS-based nickel catalyst outperformed the XANTPHOS-based nickel catalyst by a significant margin. Additionally, the NIXANTPHOS ligand will be deprotonated under the nickel-catalyzed reaction conditions outlined in these arylation reactions.

**Scheme 6:**
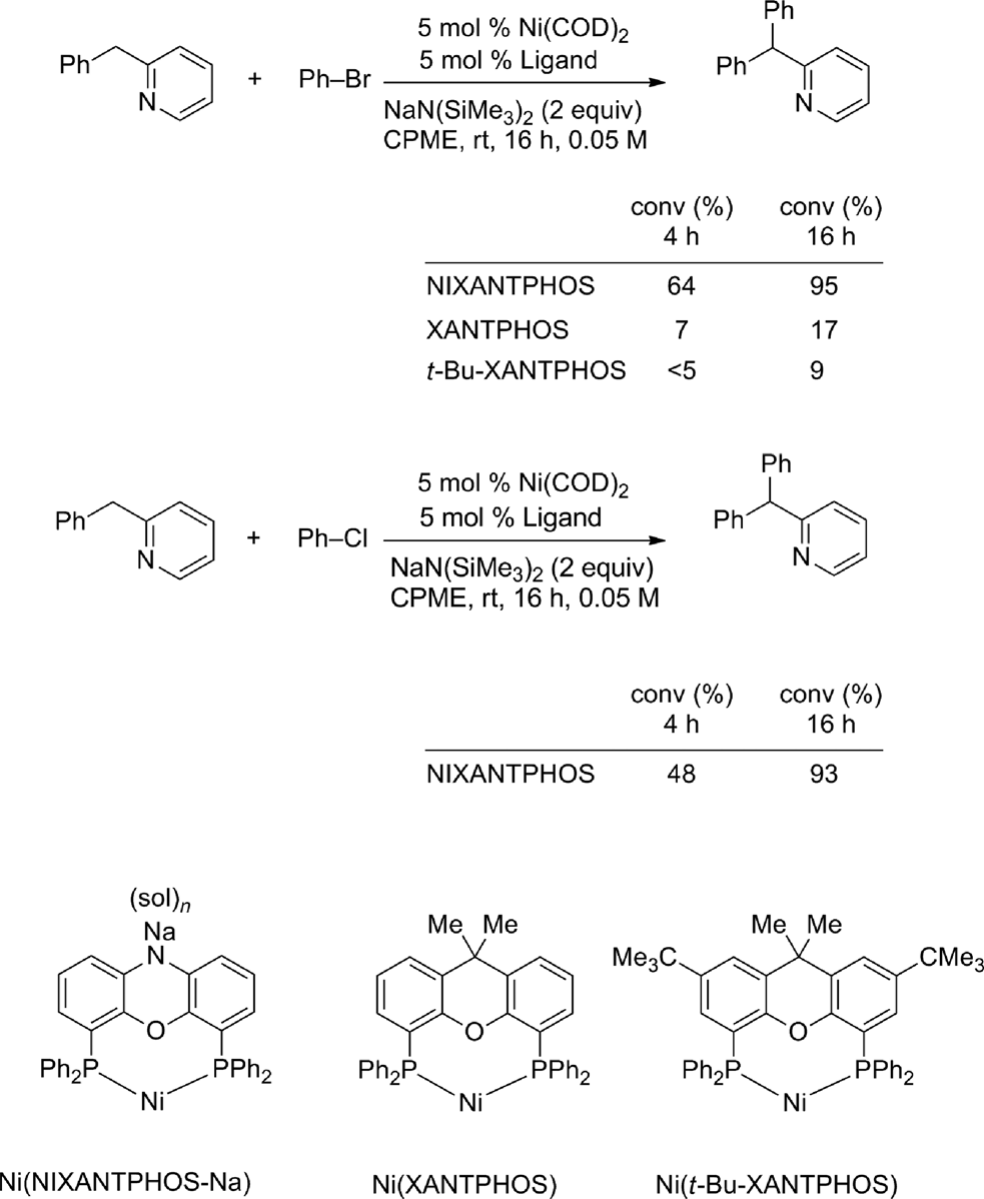
Reactivity comparisons with Ni-based catalysts.

To probe this difference in reactivity, we compared conversions of NIXANTPHOS- and XANTPHOS-based nickel catalysts in the arylation of 2-benzylpyridine with bromobenzene. As shown in Scheme 6, the Ni(NIXANTPHOS)-based catalyst exhibited higher conversion (64%) after 4 h at room temperature than the XANTPHOS analogue (7% conversion by ^1^H NMR). The presence of a significant quantity of precipitate in the reaction with XANTPHOS was cause for concern, however. We isolated the precipitate by filtration, but attempts to redissolve it in common organic solvents for the purpose of characterization were thwarted by the insolubility. It has been reported that Pd(XANTPHOS)_2_ also exhibits very low solubility.^33^

We were worried that precipitate formation would decrease the amount of active catalyst in the solution. We therefore synthesized the structurally similar 4,7-di-*tert*-butyl-XANTPHOS (*t*-Bu-XANTPHOS, Scheme 6, see ESI† for details). Palladium complexes of *t*-Bu-XANTPHOS exhibit greater solubility than analogous XANTPHOS complexes in palladium catalyzed reactions.^28^ When *t*-Bu-XANTPHOS was used in the nickel-catalyzed reactions, no solubility problems were encountered. However, we were surprised to find that under the same conditions the reaction with *t*-Bu-XANTPHOS exhibited *lower* conversions than both NIXANTPHOS- and XANTPHOS-based catalysts. It is possible that transmetallation is turnover limiting and the more hindered Ni(*t*-Bu-XANTPHOS)-based catalyst undergoes slower transmetallation.

When the reaction was conducted with the Ni(NIXANTPHOS)-based catalyst, 2-benzylpyridine and chlorobenzene, the conversion was 48% after 4 h. The similarity of conversion in the reactions with chloro and bromobenzene under otherwise identical conditions suggests that oxidative addition is not turnover limiting (Scheme 6).

At this point, we can conclude that the Ni(NIXANTPHOS)-based catalyst indeed exhibits much higher activity than the structurally related XANTPHOS derived catalysts. Recall also that, as noted in the discussion of the initial screening, the Ni(NIXANTPHOS) catalyst outperformed the other 36 mono- and bidentate phosphines of the initial reactivity screen. Determination of the origin of the difference in reactivity between Ni(NIXANTPHOS)- and Ni(NIXANTPHOS)-based catalysts has proven challenging in the arylation of 2-benzylpyridine under our reaction conditions.

### 2.5 Determination of the reactivity of the Ni(NIXANTPHOS)-based catalyst in the arylation of 2-pyridylmethyl amines

As outlined in eqn (1), we previously demonstrated that the reactivity of the Pd(NIXANTPHOS)-based catalyst is remarkable compared to other palladium phosphine-based catalyst. Our hypothesis is this is due to the deprotonation of the palladium-ligated NIXANTPHOS ligand, which gives rise to a heterobimetallic catalyst that exhibits increased reactivity due to cooperativity between the palladium and main group metal.17*b* The significance of the nickel results above is that similar high reactivity is observed with the Ni(NIXANTPHOS)-based catalyst, suggesting that perhaps the exceptional reactivity imparted to palladium by NIXANTPHOS is likewise translatable to nickel.

**Scheme 7:**
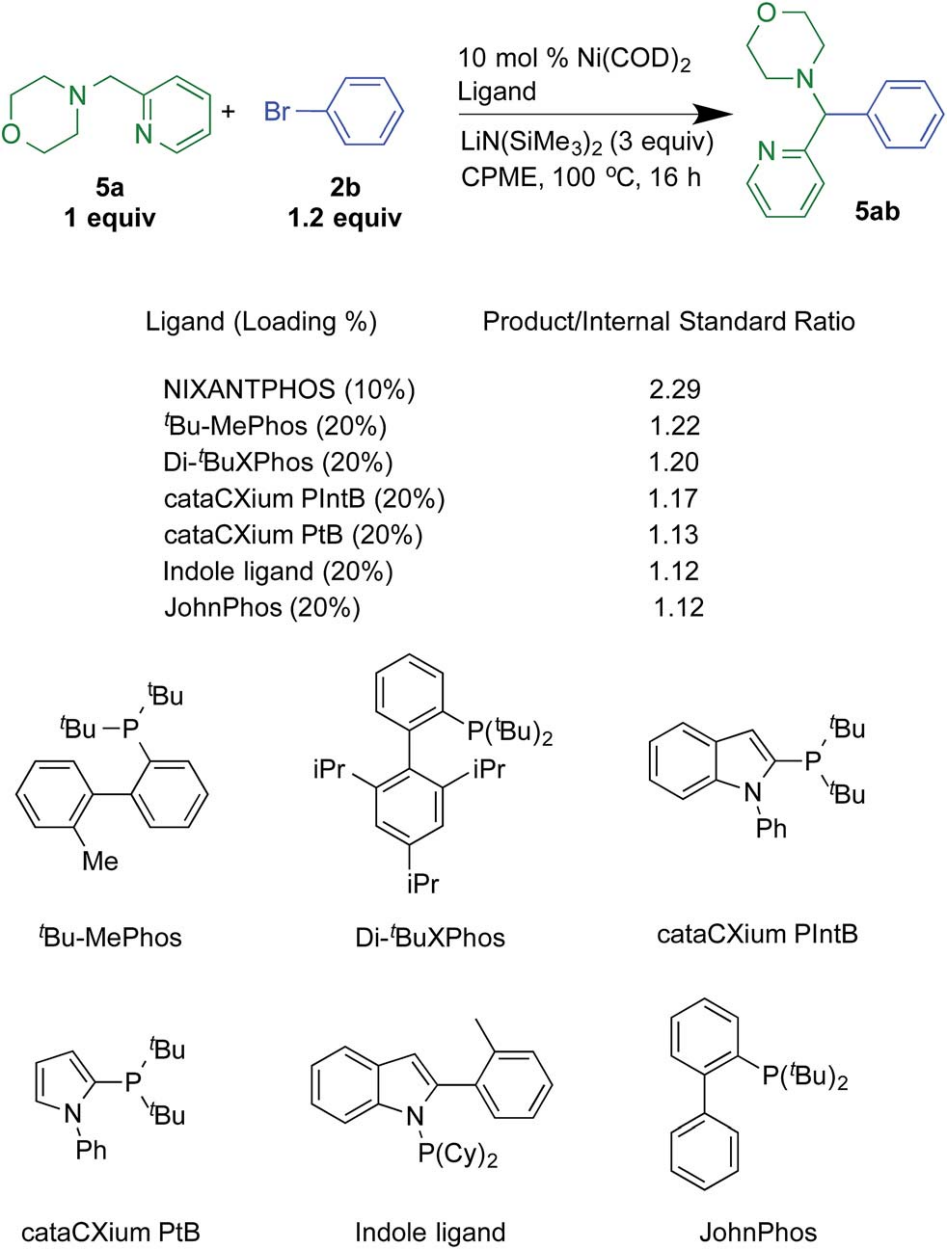
Reactivity comparisons with Ni-based catalysts and 2-pyridylmethyl amine **5a** with bromobenzene.

To further explore this possibility, we compared the reactivity of the Ni(NIXANTPHOS)-based catalyst to 37 different Ni(phosphine)-based catalysts using microscale techniques in a novel deprotonative cross-coupling process of a 2-pyridylmethyl amine with bromobenzene and LiN(SiMe_3_)_2_ as base (see ESI† for a list of all ligands and details). This reaction and the results are illustrated in Scheme 7. The Ni(NIXANTPHOS)-based catalyst exhibited the highest product/internal standard ratio (2.29), outperforming the other 37 ligands. The other ligands with the highest product/internal standard ratio were ^*t*^Bu-MePhos (1.22), di-^*t*^BuXPhos (1.20), cataCXium PIntB (1.17), JohnPhos (1.13), Kwong's indole ligand (1.12)^34^ and CataXCium PtB (1.12).^35^

**Scheme 8:**
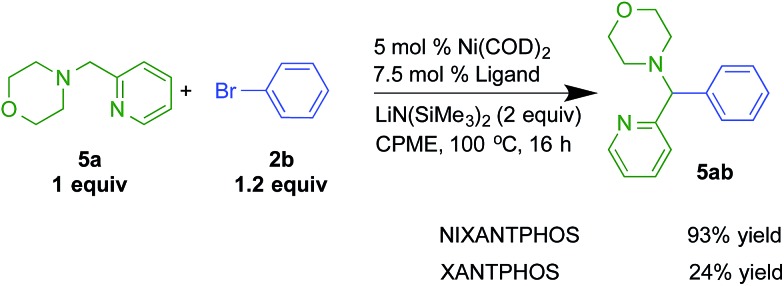
Reactivity comparisons with Ni(NIXANTPHOS) and Ni(XANTPHOS)-based catalysts in the arylation of 2-pyridylmethyl amine **5a** with bromobenzene.

The results with the Ni(NIXANTPHOS)-based catalyst was scaled to laboratory scale using 5 mol% Ni(COD)_2_ and 7.5 mol% NIXANTPHOS. The isolated yield was 93% (Scheme 8). The XANTPHOS based catalyst, however, exhibited only 24% conversion under the same reaction conditions.

## 3 Conclusions

In summary, we have presented an efficient method to form triarylmethane derivatives from diarylmethanes and aryl bromides and chlorides employing a Ni(NIXANTPHOS)-based catalyst. The method enables functionalization of C(sp^3^)–H's and does not require prefunctionalized organometallic reagents. A reactivity comparison between NIXANTPHOS- and XANTPHOS-based catalysts points to enhanced reactivity of NIXANTPHOS-based nickel catalyst. To test the generality of the Ni(NIXANTPHOS)-based catalyst, a novel arylation of a 2-pyridylmethyl amine was examined. Out of the 37 mono- and bidentate cross-coupling ligands examined with Ni(COD)_2_, NIXANTPHOS again showed the highest reactivity. These results are the first hint that the exceptional reactivity of NIXANTPHOS-based palladium catalysts may be translatable to other transition metal catalysts. This topic is currently under investigation in our research group.

## Supplementary Material

Supplementary information
