# Supplementary material for: Nickel-catalyzed arylation of heteroaryl-containing diarylmethanes: exceptional reactivity of the Ni(NIXANTPHOS)-based catalyst
Source: Chem Sci. 2015 Oct 26;7(1):611–8. doi: 10.1039/c5sc03704b (PMC4869726; doi:10.1039/c5sc03704b)
Supplement: Supplementary file 1 [file SC-007-C5SC03704B-s001.pdf]

Electronic Supplementary Material (ESI) for Chemical Science.
This journal is © The Royal Society of Chemistry 2015

SUPPORTING INFORMATION

Nickel–Catalyzed Arylation of Heteroaryl-containing Diarylmethanes: Exceptional Reactivity of the Ni(NIXANTPHOS)-based Catalyst

Xinyu Cao[a, b], Sheng–Chun Sha[b], Minyan Li[b], Byeong-Seon Kim[b], Catherine Morgan[b], Rudan Huang[a], Xiaodong Yang[c], and Patrick J. Walsh*[, b]

Key Laboratory of Cluster Science of Ministry of Education, School of Chemistry, Beijing Institute of Technology, Beijing 100081, PR China

Department of Chemistry, University of Pennsylvania, 231 S. 34th St., Philadelphia, PA 19104, USA

Key Laboratory of Medicinal Chemistry for Natural Resource, School of Chemical Science and Technology, Yunnan University, Kunming, 650091, PR China

E-mail: pwalsh@sas.upenn.edu

TABLE OF CONTENTS

1. General Methods ……………………………………………………………………………S2

2. Procedure and Characterization for the Formation of Triarylmethanes by Ni–Catalyzed Reactions ………………………………………………………………………………………S2

3. High–throughput Experimentation Screenings……………………………………..……. ………S9

4. References ………………………………………………………………………...…………S12

5. Comparison of Ligands ………………………………………………………………………S13

6. NMR Spectra …………………………………………………………..…………………S11

General Methods: All reactions were carried out under dry nitrogen using oven–dried glassware and standard Schlenk or vacuum line techniques. Air– and moisture–sensitive solutions were handled under nitrogen and transferred via syringe. Anhydrous cyclopentyl methyl ether (CPME) was purchased from Sigma–Aldrich and directly used without further purification. Unless otherwise stated, reagents were commercially available and used as purchased without further purification. Chemicals were purchased from Sigma–Aldrich, Acros, Alfa Aesar or Matrix Scientific, and solvents were purchased from Fisher Scientific. The progress of the reactions was monitored by thin–layer chromatography using Whatman Partisil K6F 250 μm precoated 60 Å silica gel plates and visualized by short–wave ultraviolet light as well as by treatment with iodine. Flash chromatography was performed with silica gel (230–400 mesh, Silicycle). The NMR spectra were obtained using a Brüker 500 MHz Fourier–transform NMR spectrometer at 500 and 125 MHz, respectively. Chemical shifts are reported in units of parts per million (ppm) downfield from tetramethylsilane (TMS), and all coupling constants are reported in hertz. The infrared spectra were obtained with KBr plates using a Perkin–Elmer Spectrum 100 Series FTIR spectrometer. High resolution mass spectrometry (HRMS) data were obtained on a Waters LC–TOF mass spectrometer (model LCT–XE Premier) using chemical ionization (CI) or electrospray ionization (ESI) in positive or negative mode, depending on the analyte. Melting points were determined on a Unimelt Thomas–Hoover melting point apparatus and are uncorrected.

Preparation of 4,7-di-*tert*-butyl-XANTPHOS.

4,7-di-*tert*-butyl-XANTPHOS was prepared according to literature procedures.[1] The spectroscopic data match the previously reported data.[1]

General Procedure: Ni–Catalyzed Arylation with heteroaryl-containing diarylmethanes. An oven–dried 10 mL reaction vial equipped with a stir bar was charged with $NaN(SiMe_3)_2$ (36.7 mg, 0.20 mmol, 2 equiv) under a nitrogen atmosphere. A solution (from a stock solution) of $Ni(COD)_2$ (2.75 mg, 0.010

mmol) and NIXANTPHOS (5.52 mg, 0.010 mmol) in 2 mL of dry CPME was taken up by syringe and added to the reaction vial under nitrogen. After stirring for 5 min at 24 ℃, 2–benzylpyridine (16.1 μL, 0.10 mmol, 1 equiv) was added to the reaction mixture followed by 1–bromo–4–*tert*–butylbenzene (26.1 μL, 0.15 mmol, 1.5 equiv). Note that diarylmethanes or aryl halides in a solid form were added to the reaction vial prior to $NaN(SiMe_3)_2$. The reaction mixture was stirred for 16 h at 110 ℃, quenched with three drops of H_2O, diluted with 3 mL of ethyl acetate, and filtered over a pad of $MgSO_4$ and silica. The pad was rinsed with 20 mL ethyl acetate, and the solution was concentrated *in vacuo*. The crude material was loaded onto a silica gel column and purified by flash chromatography with EtOAc:hexanes = 1:9.

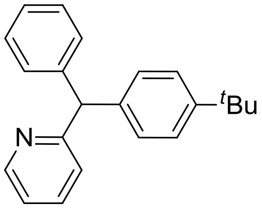

(4–*tert*–Butylphenyl)(2–pyridyl)phenylmethane (3aa): The reaction was performed following the General Procedure with **1a** (16.1 μL, 0.1 mmol), **2a** (26.1 μL, 0.15 mmol 1–*tert*–butyl–4–chlorobenzene; 25.1 μL, 0.15 mmol for 1–*tert*–butyl–4–chlorobenzene) and $NaN(SiMe_3)_2$ (36.7 mg, 0.20 mmol). The crude product was purified by flash chromatography on silica gel (eluted with hexanes to EtOAc:hexanes = 5:95 to 1:9) to give the product (29.8 mg, 99% yield for 1–*tert*–butyl–4–bromobenzene; 29.8 mg, 99 % yield for 1–*tert*–butyl–4–chlorobenzene) as a colorless oil. R_f = 0.3 (EtOAc:hexanes = 1:9). The spectroscopic data match the previously reported data.[2]

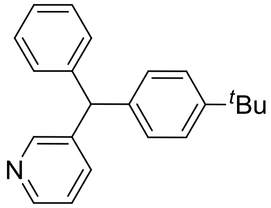

(4–*tert*–Butylphenyl)(3–pyridyl)phenylmethane (3ba): The reaction was performed following the General Procedure with **1b** (16.2 μL, 0.1 mmol), **2a** (26.1 μL, 0.15 mmol) and $NaN(SiMe_3)_2$ (73.4 mg, 0.40 mmol). The crude product was purified by flash chromatography on silica gel (eluted with hexanes to EtOAc:hexanes = 2:8 to 3:7) to give the product (20.2 mg, 67% yield) as a colorless oil. R_f = 0.50 (EtOAc:hexanes = 3:7). The spectroscopic data match the previously reported data.[2]

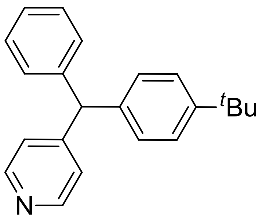

(4–*tert*–Butylphenyl)(4–pyridyl)phenylmethane (3ca): The reaction was performed following the General Procedure with **1c** (16.0 μL, 0.1 mmol), **2a**

(26.1 μL, 0.15 mmol) and $LiN(SiMe_3)_2$ (41.7 mg, 0.25 mmol). The crude product was purified by flash chromatography on silica gel (eluted with hexanes to EtOAc:hexanes = 2:8 to 3:7) to give the product (28.9 mg, 96% yield) as a colorless oil. R_f = 0.33 (EtOAc:hexanes = 3:7). The spectroscopic data match the previously reported data.[2]

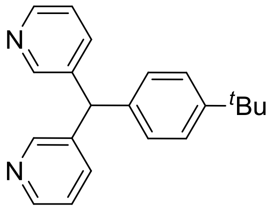

3,3'-((4-(*tert*-Butyl)phenyl)methylene)dipyridine (3da): The reaction was performed following the General Procedure with **1d** (17.0 mg, 0.1 mmol), **2a** (26.1 μL, 0.15 mmol) and $LiN(SiMe_3)_2$ (33.3 mg, 0.20 mmol). The crude product was purified by flash chromatography on silica gel (eluted with hexanes to methanol:DCM = 1:99 to 5:95) to give the product (16.9 mg, 94% yield) as a colorless oil. R_f = 0.3 (EtOAc:hexanes = 5:95). ^{1}H NMR (500 MHz, $CDCl_3$): δ 8.50 – 8.49 (d, *J* = 5.0 Hz, 2H), 8.44 (s, 2H), 7.42 – 7.41 (d, *J* = 5.0 Hz, 2H), 7.34 – 7.33 (d, *J* = 5.0 Hz, 2H), 7.25 – 7.23 (m, 2H), 7.02 – 7.01 (d, *J* = 5.0 Hz, 2H), 5.53 (s, 1H), 1.30 (s, 9H) ppm; ^{13}C{^{1}H} NMR (125 MHz, $CDCl_3$): δ 150.9, 150.2, 148.3, 138.7, 138.5, 136.7, 128.9, 125.9, 123.6, 51.7, 34.7, 31.5 ppm; IR (thin film): 3418, 2963, 2928, 2869, 2088, 1659, 1651, 1645, 1634, 1575, 1514, 1477, 1422, 1364, 1270, 1108, 1044, 1026, 715, 666 cm^{-1}; HRMS calculated for $C_{21}H_{23}N_2$ 303.1861, observed 303.1860 $[M+H]^+$.

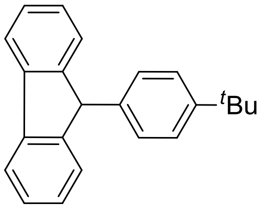

9–(4–*tert*–Butylphenyl)fluorene (3ea): The reaction was performed following the General Procedure with **1e** (16.6 mg, 0.1 mmol), **2a** (26.1 μL, 0.15 mmol) and $NaN(SiMe_3)_2$ (27.5 mg, 0.15 mmol). The crude product was purified by flash chromatography on silica gel (eluted with hexanes to EtOAc:hexanes = 2:98) to give the product (28.3 mg, 94% yield) as a white solid. R_f = 0.3 (hexanes). The spectroscopic data match the previously reported data.[3]

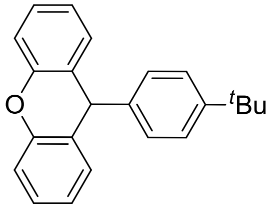

9–(4–*tert*–Butylphenyl)xanthene (3fa): The reaction was performed following the General Procedure with **1f** (16.1 μL, 0.1 mmol), **2a** (26 μL, 0.15 mmol) and $NaN(SiMe_3)_2$ (36.7 mg, 0.20 mmol). The crude product was purified by flash

chromatography on silica gel (eluted with hexanes to EtOAc:hexanes = 2:98) to give the product (29.5 mg, 99% yield) as a white solid. R_f = 0.17 (hexanes). The spectroscopic data match the previously reported data.[2]

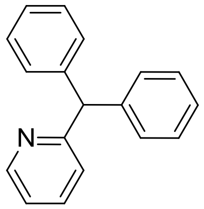

2–Pyridyldiphenylmethane (3ab): The reaction was performed following the General Procedure with **1a** (16.1 μL, 0.1 mmol), **2b** or **4b** (15.8 μL, 0.15 mmol for **2b** bromobenzene; 15.2 μL, 0.15 mmol for **4b** chlorobenzene) and $NaN(SiMe_3)_2$ (36.7 mg, 0.20 mmol). The crude product was purified by flash chromatography on silica gel (eluted with hexanes to EtOAc:hexanes = 5:95 to 1:9) to give the product (22.8 mg, 93% yield for bromobenzene; 22.6 mg, 92 % yield for chlorobenzene) as a colorless oil. R_f = 0.4 (EtOAc:hexanes = 2:8). The spectroscopic data match the previously reported data.[4]

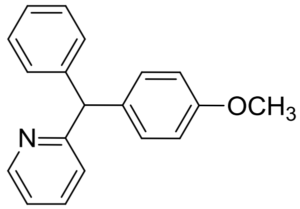

(4–Methoxyphenyl)(2–pyridyl)phenylmethane (3ac): The reaction was performed following the General Procedure with **1a** (16.1 μL, 0.1 mmol), **2c** or **4c** (18.8 μL, 0.15 mmol for **2c** 4-bromoanisole; 18.4 μL, 0.15 mmol for **4c** 4-chloroanisole) and $NaN(SiMe_3)_2$ (36.7 mg, 0.20 mmol). The crude product was purified by flash chromatography on silica gel (eluted with hexanes to EtOAc:hexanes = 5:95 to 1:9) to give the product (25.0 mg, 91% yield for 4-bromoanisole; 27.2 mg, 99% yield for 4-chloroanisole) as a colorless oil. R_f = 0.45 (EtOAc:hexanes = 2:8). The spectroscopic data match the previously reported data.[4]

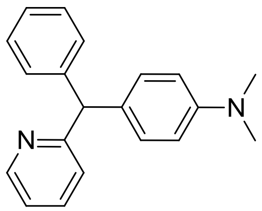

(4–*N,N*–Dimethylaminophenyl)(2–pyridyl)phenylmethane (3ad): The reaction was performed following the General Procedure with **1a** (16.1 μL, 0.1 mmol), **2d** (30.0 mg, 0.15 mmol) and $NaN(SiMe_3)_2$ (36.7 mg, 0.20 mmol). The crude product was purified by flash chromatography on silica gel (eluted with hexanes to EtOAc:hexanes = 1:9 to 2:8) to give the product (23.9 mg, 83% yield) as a colorless oil. R_f = 0.2 (EtOAc:hexanes = 2:8). The spectroscopic data match the previously reported data.[4]

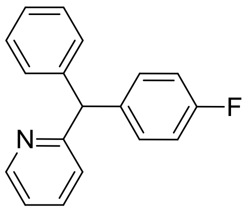

(4–Fluorophenyl)(2–pyridyl)phenylmethane (3ae): The reaction was performed following the General Procedure with **1a** (16.1 μL, 0.1 mmol), **2e** or **4e** (16.5 μL, 0.15 mmol for **2e** 1-bromo-4-fluorobenzene; 16.0 μL, 0.15 mmol for **4e** 1-chloro-4-fluorobenzene) and $NaN(SiMe_3)_2$ (36.7 mg, 0.20 mmol). The crude product was purified by flash chromatography on silica gel (eluted with hexanes to EtOAc:hexanes = 5:95 to 1:9) to give the product (21.8 mg, 83% yield for 1-bromo-4-fluorobenzene; 23.7 mg, 90% yield for 1-chloro-4-fluorobenzene) as a colorless oil. R_f = 0.5 (EtOAc:hexanes = 2:8). The spectroscopic data match the previously reported data.[4]

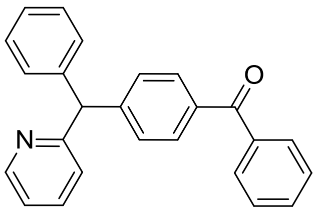

Phenyl(4-(phenyl(pyridin-2-yl)methyl)phenyl)methanone (3af): The reaction was performed following the General Procedure with **1a** (16.1 μL, 0.1 mmol), **2f** or **4f** (32.5 mg, 0.15 mmol for **2f** 4-bromobenzophenone; 32.5 mg, 0.15 mmol for **4f** 4-chlorobenzophenone) and $NaN(SiMe_3)_2$ (36.7 mg, 0.20 mmol). The crude product was purified by flash chromatography on silica gel (eluted with hexanes to EtOAc:hexanes = 5:95 to 1:9) to give the product (25.8 mg, 74% yield for 4-bromobenzophenone; 32.1 mg, 92% yield for 4-chlorobenzophenone) as a colorless solid. R_f = 0.3 (EtOAc:hexanes = 1:9) ^{1}H NMR (500 MHz, $CDCl_3$): δ 8.63 – 8.62 (d, *J* = 4.0 Hz, 1H), 7.80 (s, 1H), 7.79 (d, *J* = 1.5 Hz, 1H), 7.76 (s, 1H), 7.75 (s, 1H), 7.65 – 7.60 (dt, *J* = 7.5Hz, 1.5 Hz, 1H), 7.58 – 7.55 (t, *J* = 7.5 Hz, 1H), 7.47 – 7.44 (t, *J* = 7.5 Hz, 2H), 7.32 – 7.29 (t, *J* = 8.5 Hz, 4H), 7.27 – 7.25 (t, *J* = 3.5 Hz, 1H), 7.21 – 7.15 (m, 3H), 7.12 – 7.11 (d, *J* = 7.5 Hz, 1H), 5.77 (s, 1H) ppm; $^{13}C\{^1H\}$ NMR (125 MHz, $CDCl_3$): δ 196.5, 162.6, 149.9, 147.9, 142.1, 137.9, 136.8, 136.0, 132.5, 130.5, 130.2, 129.5, 129.5, 128.8, 128.4, 127.1, 124.0, 121.9, 59.5 ppm; IR (thin film): 3438, 3061, 2926, 2854, 1652, 1607, 1588, 1494, 1470, 1447, 1433, 1411, 1317, 1279, 1178, 1149, 11076, 939, 925, 844, 773, 700, 666 cm^{-1}; HRMS calc'd for $C_{25}H_{20}NO$ 350.1545, observed 350.1544 $[M+H]^+$**;** Melting range: 133–135 ^oC.

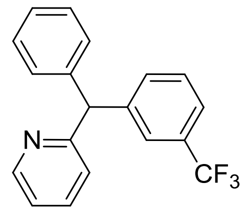

2-(Phenyl(3-(trifluoromethyl)phenyl)methyl)pyridine (3ag): The reaction was performed following the General Procedure with **1a** (16.1 μL, 0.1 mmol), **2g** or **4g** (20.9 μL, 0.15 mmol for **2g** 3-bromobenzotrifluoride; 27.1 mg, 0.15 mmol for **4g** 3-chlorobenzotrifluoride) and $NaN(SiMe_3)_2$ (36.7 mg, 0.20 mmol). The crude product was purified by flash chromatography on silica gel (eluted with hexanes to EtOAc:hexanes = 5:95 to 1:9) to give the product (26.9 mg, 86% yield for 3-bromobenzotrifluoride; 28.2 mg, 90% yield for 3-chlorobenzotrifluoride) as a colorless oil. R_f = 0.6 (EtOAc:hexanes = 2:8) ^{1}H NMR (500 MHz, $CDCl_3$): δ 8.61 – 8.60 (m, 1H), 7.64 – 7.61 (dt, *J* = 7.5 Hz, 2.0 Hz, 1H), 7.49–7.47 (d, *J* = 7.5 Hz, 1H), 7.45 (s, 1H), 7.42 – 7.37 (m, 2H), 7.30 (s, 1H), 7.29 (s, 1H), 7.27 – 7.26 (d, *J* = 6.5 Hz, 1H), 7.18 – 7.14 (m, 3H), 7.09 – 7.07 (d, *J* = 7.5 Hz, 1H), 5.72 (s, 1H) ppm; ^{13}C{^{1}H} NMR (125 MHz, $CDCl_3$): δ 162.4, 150.0, 144.0, 142.1, 136.9, 133.0, 130.9 (q, *J* = 32 Hz), 129.5, 129.0, 128.9, 127.1, 126.3 (q, *J* = 4 Hz), 124.0, 123.7 (q, *J* = 4 Hz), 124.4 (q, *J* = 273 Hz), 122.0, 59.3 ppm; IR (thin film): 3431, 3064, 3029, 2925, 2854, 1954, 1589, 1572, 1495, 1470, 1447, 1434, 1329, 1247, 1164, 1123, 1098, 1075, 1051, 1032, 995, 910, 800, 749, 701 cm^{-1}; HRMS calculated for $C_{19}H_{15}NF_3$ 314.1157, observed 314.1162 $[M+H]^+$.

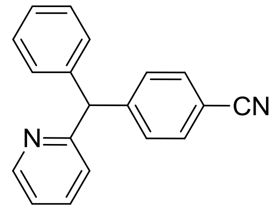

4-(Phenyl(pyridin-2-yl)methyl)benzonitrile (3ah): The reaction was performed following the General Procedure with **1a** (16.1 μL, 0.1 mmol), **2h** (20.6 mg, 0.15 mmol) and $NaN(SiMe_3)_2$ (36.7 mg, 0.20 mmol). The crude product was purified by flash chromatography on silica gel (eluted with hexanes to EtOAc:hexanes = 5:95 to 2:8) to give the product (21.4 mg, 79% yield) as a colorless oil. R_f = 0.3 (EtOAc:hexanes = 2:8). The spectroscopic data match the previously reported data.[4]

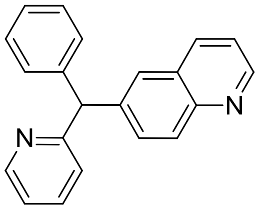

6-(Phenyl(pyridin-2-yl)methyl)quinoline (3ai): The reaction was performed following the General Procedure with **1a** (16.1 μL, 0.1 mmol), **2i** (26.1 μL, 0.15 mmol) and $NaN(SiMe_3)_2$ (36.7 mg, 0.20 mmol). The crude product was purified by flash chromatography on silica gel (eluted with hexanes to EtOAc:hexanes = 3:7 to 6:4) to give the product (23.4 mg, 73% yield) as a colorless oil. R_f = 0.2 (EtOAc:hexanes = 6:4). ^{1}H

NMR (500 MHz, $CDCl_3$): δ 8.87 – 8.86 (m, 1H), 8.63 – 8.62 (d, J = 5.0 Hz, 1H), 8.05 – 8.02 (m, 2H), 7.64 – 7.58 (m, 2H), 7.52 (s, 1H), 7.35 – 7.31 (m, 3H), 7.27 – 7.21 (m, 3H), 7.18 – 7.13 (m, 2H), 5.89 (s, 1H) ppm; $^{13}C\{^1H\}$ NMR (125 MHz, $CDCl_3$): δ 162.8, 150.4, 149.9, 147.5, 142.4, 141.5, 136.8, 136.2, 131.7, 129.6, 128.8, (128.8), 128.4, 127.8, 127.0, 124.1, 121.9, 121.4, 59.4 ppm; IR (thin film): 3390, 3060, 3027, 2924, 2216, 1952, 1808, 1667, 1588, 1570, 1496, 1470, 1433, 1328, 1252, 1188, 1156, 1118, 1076, 1051, 1032, 909, 832, 800, 749, 730, 701 cm^{-1}; HRMS calculated for $C_{21}H_{17}N_2$ 297.1392, observed 297.1392 $[M+H]^+$.

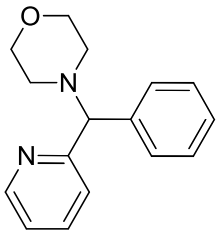

4-(phenyl(pyridin-2-yl)methyl)morpholine (5ab): An oven–dried 10 mL reaction vial equipped with a stir bar was charged with $LiN(SiMe_3)_2$ (33.3 mg, 0.20 mmol, 2 equiv) under a nitrogen atmosphere. A solution (from a stock solution) of $Ni(COD)_2$ (1.4 mg, 0.005 mmol) and NIXANTPHOS (8.3 mg, 0.0075 mmol) in 1 mL of dry CPME was taken up by syringe and added to the reaction vial under nitrogen. After stirring for 5 min at 24 ℃, 4-(pyridin-2-ylmethyl)morpholine **5a** (17.3 μL, 0.1 mmol, 1 equiv) was added to the reaction mixture followed by bromobenzene **2b** (12.6 μL, 0.12 mmol, 1.2 equiv). The reaction mixture was stirred for 16 h at 100 ℃, quenched with three drops of H_2O, diluted with 3 mL of ethyl acetate, and filtered over a pad of $MgSO_4$ and silica. The pad was rinsed with 20 mL ethyl acetate, and the solution was concentrated *in vacuo*. The crude product was purified by flash chromatography on silica gel (eluted with hexanes to EtOAc:hexanes = 2:3) to give the product (23.6 mg, 93% yield) as a white oil. R_f = 0.25 (EtOAc:hexanes = 2:3). ^1H NMR (500 MHz, $CDCl_3$): δ 8.51 – 8.50 (d, J = 5.0 Hz, 1H), 7.61 – 7.55 (m, 2H), 7.51 – 7.49 (d, J = 7.5 Hz, 2H), 7.29 – 7.26 (t, J = 7.5 Hz, 2H), 7.20 – 7.17 (t, J = 7.5 Hz, 1H), 7.08 – 7.05 (m, 1H), 4.42 (s, 1H), 3.75 – 3.70 (m, 4H), 2.47 – 2.35 (m, 4H) ppm; $^{13}C\{^1H\}$ NMR (125 MHz, $CDCl_3$): δ 161.8, 149.4, 140.7, 136.8, 128.7, 128.4, 127.5, 122.3, 122.2, 78.5, 67.1, 52.7 ppm; IR (thin film): 3061, 2958, 2851, 2811, 2762, 1587, 1570, 1493, 1470, 1451, 1432, 1395, 1279, 1246, 1117, 1070, 1012 cm^{-1}; HRMS calc'd for $C_{16}H_{19}N_2O$ 255.1497, observed 255.1498 $[M+H]^+$.

High-throughput experimentation screenings

(1) Ligands screening of coupling between 2–benzylpyridine 1a and 1–bromo–4–*tert*–butylbenzene 2a

Experiments were set up inside a glovebox under a nitrogen atmosphere. Two 24-well aluminum blocks containing 1 mL glass vials were predosed with $Ni(COD)_2$ (1 μmol) and the phosphine ligands (2 μmol for monodentate ligands and 1 μmol for bidentate ligands) in THF. The solvent was removed to dryness using a GeneVac and $NaN(SiMe_3)_2$ (30 μmol) in THF was added to the ligand/catalyst mixture. The solvent was removed on the GeneVac and a parylene stir bar was then added to each reaction vial. 2–benzylpyridine **1a** (12 μmol/reaction) and 1–bromo–4–*tert*–butylbenzene **2a** (10 μmol) were then dosed together into each reaction vial as a solution in CPME (100 μL, 0.1 M). The 24-well plates were then sealed and stirred for 16 h at 110°C then cooled to room temperature.

Work up:

Upon opening the plate to air, 500 μL of a solution of biphenyl (used as internal standard to measure HPLC yields) in acetonitrile (0.002 mol/L) was added into each vial. The plate was covered again and the vials stirred for 10 min. to ensure good homogenization. Into a separate 96-well LC block was added 700 μL of acetonitrile, followed by 25 μL of the diluted reaction mixtures. The LC block was then sealed with a silicon-rubber storage mat and mounted on an automated HPLC instrument for analysis.

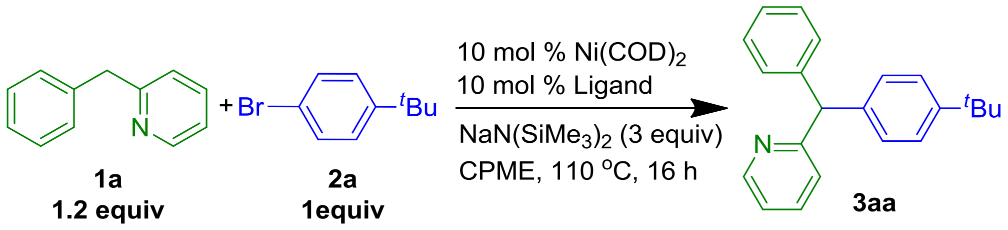

Entry	Ligand	Prod/IS[a]
1	2–(Di–*t*–butylphosphino)biphenyl (JohnPhos)	0.90
2	2–Dicyclohexylphosphino–2'–(*N*,*N*–dimethylamino)biphenyl (DavePhos)	0.98
3	2–Dicyclohexylphosphino–2',6'–di–i–propoxy–1,1'–biphenyl (RuPhos)	0.76
4	2–Di–tert–butylphosphino–2',4',6' –triisopropylbiphenyl **(***t*–Bu XPhos**)**	0.82
5	2–Di–tert-butylphosphino-3,4,5,6-tetramethyl-2′,4′,6′-triisopropyl-1,1′-biphenyl (Me4–*t*–Bu XPhos**)**	0.80
6	(2–Biphenyl)dicyclohexylphosphine (Cyclohexyl JohnPhos)	0.78

7	Bis[(2–diphenylphosphino)phenyl] ether (DPEPhos)	1.08
8	1–[2–[Bis(*t*–butyl)phosphino]phenyl]–3,5–diphenyl–1H–pyrazole (Trippyphos)	0.86
9	Di(1-adamantyl)-2-dimethylaminophenylphosphine (MeDal Phos)	1.12
10	Di(1-adamantyl)-2-morpholinophenylphosphine (MorDal Phos)	1.20
11	Tri(o–tolyl)phosphine	0.49
12	5–(Di–*t*–butylphosphino)–1', 3', 5'–triphenyl–1'H–[1,4']bipyrazole (BippyPhos)	0.61
13	Tricyclohexylphosphine tetrafluoroborate (PCy_3 HBF_4)	0.23
14	Tri-*tert*-butylphosphonium tetrafluoroborate (P*t*Bu_3 HBF_4)	1.33
15	Di–tert–butyl(neopentyl)phosphine HBF_4	0.64
16	2′–(Dicyclohexylphosphino)acetophenone ethylene ketal (SymPhos)	0.70
17	2–Di–tert–butylphosphino–3–Methoxy–6–Methyl–2'–4'–6'–triisopropylbiphenyl (RockPhos)	2.91
18	2-Di-*tert*-butylphosphino-1,1'-binaphthyl (TrixiePhos)	0.84
19	2–(Di–*t*–butylphosphino)–2'–methylbiphenyl (*t*Bu–MePhos)	0.74
20	*N*,*N*'-dicyclohexyl-1-diphenylphosphanyl-formamidine (DCyF)	1.20
21	1,1'–Bis(di–*t*–butylphosphino)ferrocene (dtbpf)	1.32
22	1,1'–Bis(diisopropylphosphino)ferrocene (dippf)	1.95
23	1,1'–Bis(diphenylphosphino)ferrocene (dppf)	1.75
24	1,2,3,4,5–Pentaphenyl–1'–(di–*t*–butylphosphino)ferrocene (QPhos)	2.05
25	[1,1′–Binaphthalene]–2,2′–diylbis[diphenylphosphine] (Binap)	1.71
26	9,9–Dimethyl–4,5–bis(diphenylphosphino)xanthene (Xantphos)	4.98
27	*N*–phenyl–2–(di–*t*–butylphosphino)pyrrole (CataXCium PtB)	0.96
28	Dicyclohexyl–(1–phenylindol–2–yl)phosphane (cataCXium PInCy)	1.26
29	*N*–phenyl–2–(dicyclohexylphosphino)pyrrole (cataCXium PCy)	1.59
30	Butyldi–1–adamantylphosphine (CataCXium A)	1.49
31	Di–*t*–butyl–(1–phenylindol–2–yl)phosphane (cataCXium PIntB)	0.82

32	Benzyldi–1–adamantylphosphine (cataCXium ABn)	1.00
33	2–Dicyclohexylphosphino–2',6'–dimethoxy–1,1'–biphenyl (SPhos)	1.06
34	Sodium 2′-dicyclohexylphosphino-2,6-dimethoxy-1,1′-biphenyl-3-sulfonate hydrate (^{S}SPhos)	0.71
35	Dicyclohexyl–[3,6–dimethoxy–2–(2,4,6–triisopropylphenyl)phenyl]phosphane (Brettphos)	1.08
36	2–Dicyclohexylphosphino–2′,6′–bis(*N*,*N*–dimethylamino)biphenyl (CPhos)	1.19
37	4,6–Bis(diphenylphosphino)phenoxazine (NIXANTPHOS)	11.70

[a]Product-internal standard radio.

(1) Ligands screening of coupling between 4–(pyridin–2–ylmethyl)morpholine 5a and bromobenzene 2b:

Experiments were set up inside a glovebox under a nitrogen atmosphere. Two 24-well aluminum blocks containing 1 mL glass vials were predosed with $Ni(COD)_2$ (1 μmol) and the phosphine ligands (2 μmol for monodentate ligands and 1 μmol for bidentate ligands) in THF. The solvent was removed to dryness using a GeneVac and $NaN(SiMe_3)_2$ (30 μmol) in THF was added to the ligand/catalyst mixture. The solvent was removed on the GeneVac and a parylene stir bar was then added to each reaction vial. 4-(pyridin-2-ylmethyl)morpholine **5a** (10 μmol/reaction) and bromobenzene **2b** (12 μmol) were then dosed together into each reaction vial as a solution in CPME (100 μL, 0.1 M). The 24-well plates were then sealed and stirred for 16 h at 100°C then cooled to room temperature.

Work up:

Upon opening the plate to air, 500 μL of a solution of 4,4'-Di-tert-butylbiphenyl (used as internal standard to measure UPLC yields) in acetonitrile (0.002 mol/L) was added into each vial. The plate was covered again and the vials stirred for 10 min. to ensure good homogenization. Into a separate 96-well LC block was added 700 μL of acetonitrile, followed by 25 μL of the diluted reaction mixtures. The LC block was then sealed with a silicon-rubber storage mat and mounted on an automated UPLC instrument for analysis.

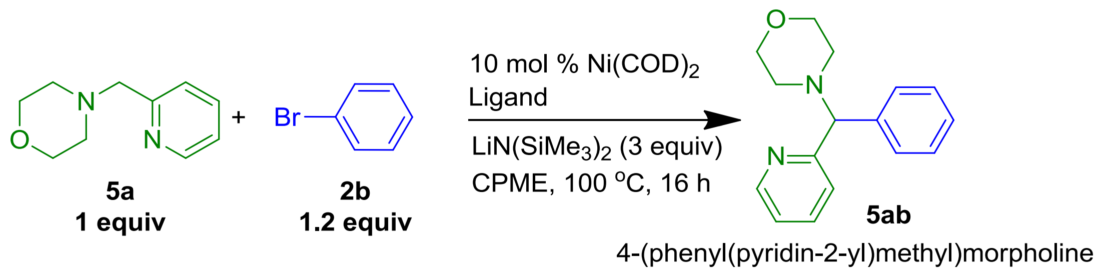

Entry	Ligand	Prod/IS[a]
1	2–(Di–*t*–butylphosphino)biphenyl (JohnPhos)	1.13
2	2–Dicyclohexylphosphino–2'–(*N*,*N*–dimethylamino)biphenyl (DavePhos)	1.03
3	Tris(2,4,6-trimethylphenyl)phosphine (PXy_3)	0.73
4	Rac-1,1′-Binaphthalene-2,2′-diyl)bis(diphenylphosphine)	0.74
5	2–Di–tert-butylphosphino-3,4,5,6-tetramethyl-2′,4′,6′-triisopropyl-1,1′-biphenyl (Me4–*t*–Bu XPhos)	1.19
6	(2–Biphenyl)dicyclohexylphosphine (Cy JohnPhos)	0.89
7	Bis[(2–diphenylphosphino)phenyl] ether (DCEPhos)	0.78
8	1–[2–[Bis(*t*–butyl)phosphino]phenyl]–3,5–diphenyl–1H–pyrazole (Trippyphos)	0.93
9	Di(1-adamantyl)-2-dimethylaminophenylphosphine (MeDal Phos)	0.13
10	Di(1-adamantyl)-2-morpholinophenylphosphine (MorDal Phos)	1.00
11	2-(Di-tert-butylphosphino)-2',4',6'- triisopropyl-3,6-dimethoxy-1,1'-biphenyl (*t*Bu-BrettPhos)	1.08
12	5–(Di–*t*–butylphosphino)–1', 3', 5'–triphenyl–1'H–[1,4']bipyrazole (BippyPhos)	1.04
13	Tricyclohexylphosphine tetrafluoroborate (PCy_3 HBF_4)	0.77
14	Tri-*tert*-butylphosphonium tetrafluoroborate (P*t*Bu_3 HBF_4)	0.75
15	Di–tert–butyl(neopentyl)phosphine HBF_4	0.75
16	2′–(Dicyclohexylphosphino)acetophenone ethylene ketal (SymPhos)	0.98
17	2–Di–tert–butylphosphino–3–Methoxy–6–Methyl–2'–4'–6'–triisopropylbiphenyl (RockPhos)	1.01
18	2-Di-*tert*-butylphosphino-1,1'-binaphthyl (TrixiePhos)	0.91

19	2-Dicyclohexylphosphino-2',4',6'-triisopropylbiphenyl	0.97
20	*N*,*N*'-dicyclohexyl-1-diphenylphosphanyl-formamidine (DCyPF)	0.74
21	2-Di-tert-butylphosphino-2',4',6'-triisopropylbiphenyl (Di-tBu-XPhos)	1.20
22	(4-(N,N-Dimethylamino)phenyl)di-tert-butyl phosphine (A^{ta}-Phos)	1.06
23	Tri(furan-2-yl)phosphine	0.81
24	1,2,3,4,5–Pentaphenyl–1'–(di–*t*–butylphosphino)ferrocene (QPhos)	1.10
25	N-(dicyclohexylphosphino)-2-(2'-methylphenyl)-1H-indole	1.12
26	2-Di-tert-butylphosphino-2'-methylbiphenyl (tBu-MePhos)	1.22
27	*N*–phenyl–2–(di–*t*–butylphosphino)pyrrole (CataXCium PtB)	1.12
28	2-(Dicyclohexylphosphino)-1-(2,4,6-trimethyl-phenyl)-1H-imidazole (cataCXium PICy)	0.95
29	*N*–phenyl–2–(dicyclohexylphosphino)pyrrole (cataCXium PCy)	0.91
30	Butyldi–1–adamantylphosphine (CataCXium A)	1.09
31	Di–*t*–butyl–(1–phenylindol–2–yl)phosphane (cataCXium PIntB)	1.17
32	Benzyldi–1–adamantylphosphine (cataCXium ABn)	1.09
33	2–Dicyclohexylphosphino–2',6'–dimethoxy–1,1'–biphenyl (SPhos)	1.04
34	(2R)-1-[(1R)-1-[Bis(1,1-dimethylethyl)phosphino]ethyl]-2-(dicyclohexylphosphino)ferrocene (Josiphos SL-J009-1)	0.20
35	Dicyclohexyl–[3,6–dimethoxy–2–(2,4,6–triisopropylphenyl)phenyl]phosphane (Brettphos)	0.94
36	2-Dicyclohexylphosphino-2'-methylbiphenyl (MePhos)	0.91
37	4,6–Bis(diphenylphosphino)phenoxazine (NIXANTPHOS) 5 mol %	1.84
38	4,6–Bis(diphenylphosphino)phenoxazine (NIXANTPHOS) 10 mol %	2.30
39	4,6–Bis(diphenylphosphino)phenoxazine (NIXANTPHOS) 15 mol %	2.78
40	4,6–Bis(diphenylphosphino)phenoxazine (NIXANTPHOS) 20 mol %	2.62
41	Triphenylphosphine	0.82
42	2-(Dicyclohexylphosphino)-1-phenylindole (cataCXium PInCy)	0.92

[a]Product-internal standard radio.

Comparison of Ligands

Preparation of Stock Solution: An oven–dried 10 mL reaction vial equipped with a stir bar was added with $Ni(COD)_2$ (1.38 mg, 0.005 mmol) and ligands (For NIXANTPHOS: 2.76 mg, 0.005 mmol; XANTPHOS: 2.89 mg, 0.005 mmol; 4,7-di-*tert*-butyl-XANTPHOS: 3.45 mg, 0.005 mmol) under a nitrogen atmosphere. Next 2 mL of dry CPME was added.

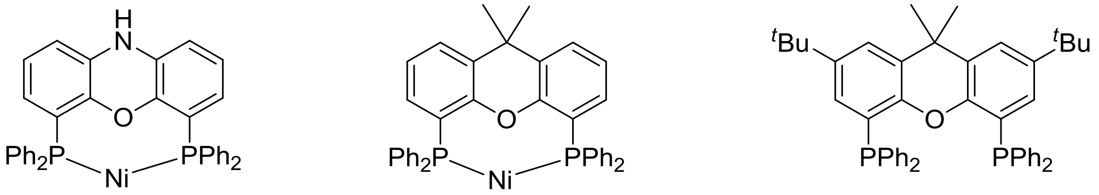

NIXANTPHOS,	XANTPHOS	^{t}BuXANTPHOS
Precipitate after 30 min	Precipitate after 5 min	No precipitate
92% AY	21% AY	3% AY

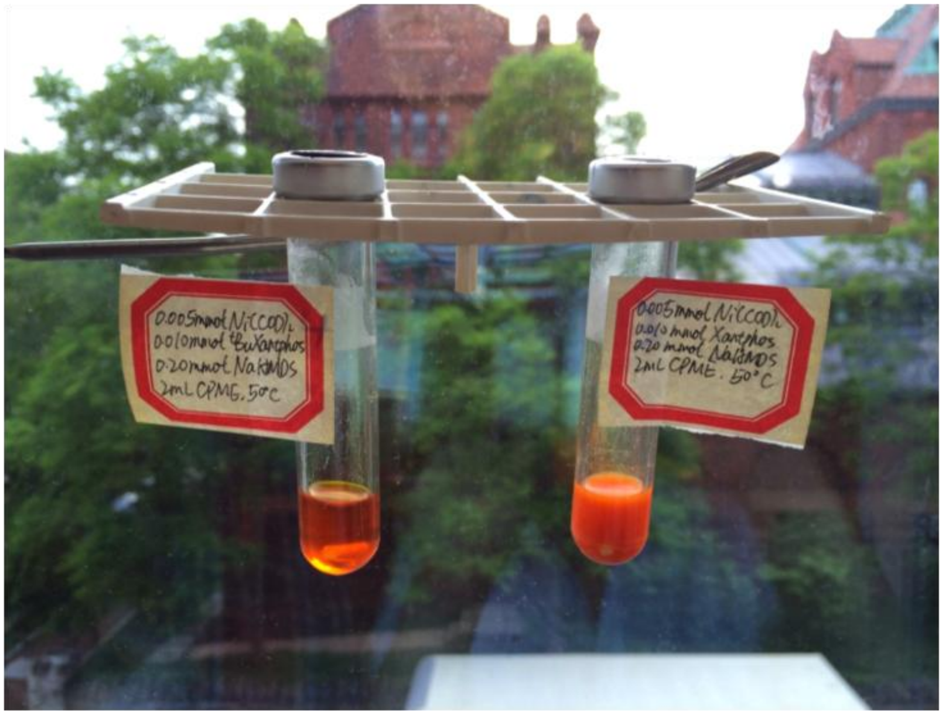

Figure S1. Stock Solutions of Nickel-4,7-di-*tert*-butyl-XANTPHOS (left) and Nickel-XANTPHOS (right) in CPME.

Cross-coupling of 2-benzyl pyridine with bromobenzene with different ligands.

The reactions were performed following the General Procedure with **1a** (16.1 μL, 0.1 mmol), **2b** (15.8 μL, 0.15 mmol for bromobenzene) and $NaN(SiMe_3)_2$ (36.7 mg, 0.20 mmol), quenched after the listed reaction time. For NIXANTPHOS (2.76 mg, 0.005 mmol), XANTPHOS (2.89 mg, 0.005 mmol), 4,7-di-*tert*-butyl-XANTPHOS (3.45 mg, 0.005 mmol).

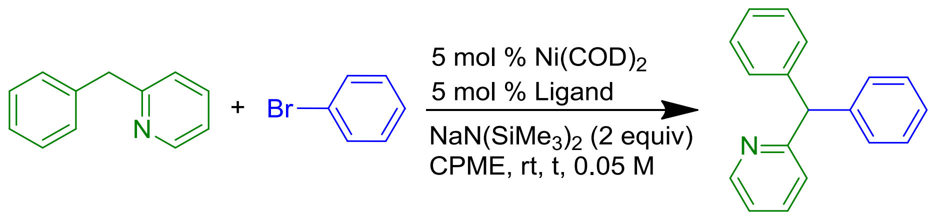

	5min	30min	1h	2h	4h	16h
**NIXANTPHOS**	3	17	34	46	64	95
**XANTPHOS**	2	2	2	9	7	17
**^{t}BuXANTPHOS**	0	0	0	0	0	9

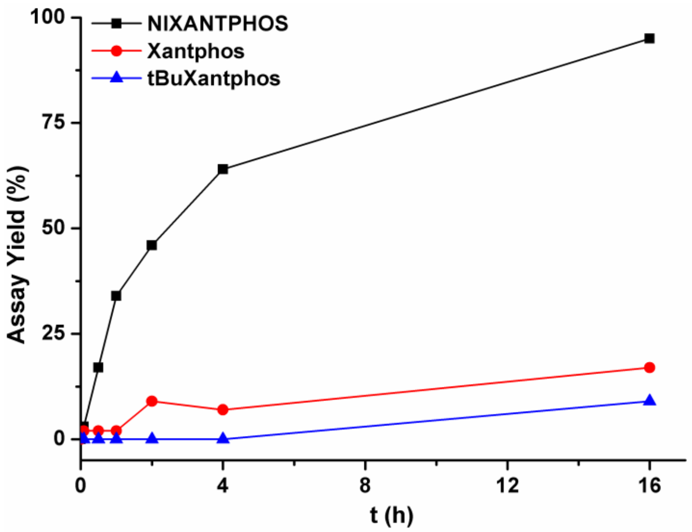

Figure S1. Assay yield at different reaction time with bromobenzene catalyzed by different ligands systems.

Cross-coupling of 2-benzyl pyridine with chlorobenzene with different ligands. The reaction was performed following the General Procedure with **1a** (16.1 μL, 0.1 mmol), **4b** (15.2 μL, 0.15 mmol for chlorobenzene) and $NaN(SiMe_3)_2$ (36.7 mg, 0.20 mmol), quenched after the listed reaction time. For NIXANTPHOS (2.76 mg, 0.005 mmol), XANTPHOS (2.89 mg, 0.005 mmol), 4,7-di-*tert*-butyl-XANTPHOS (3.45 mg, 0.005 mmol). Note that under the reaction conditions with 4,7-di-*tert*-butyl-XANTPHOS the product decomposed over time.

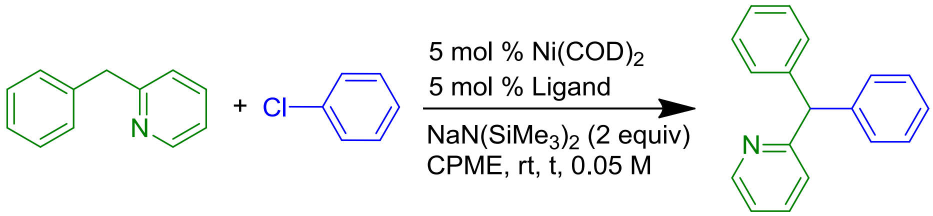

	**5min**	**30min**	**1h**	**2h**	**4h**	**16h**
**NIXANTPHOS**	3	19	23	31	48	93
**XANTPHOS**	2	3	3	3	9	9
**^{t}BuXANTPHOS**	0	0	0	4	9	0

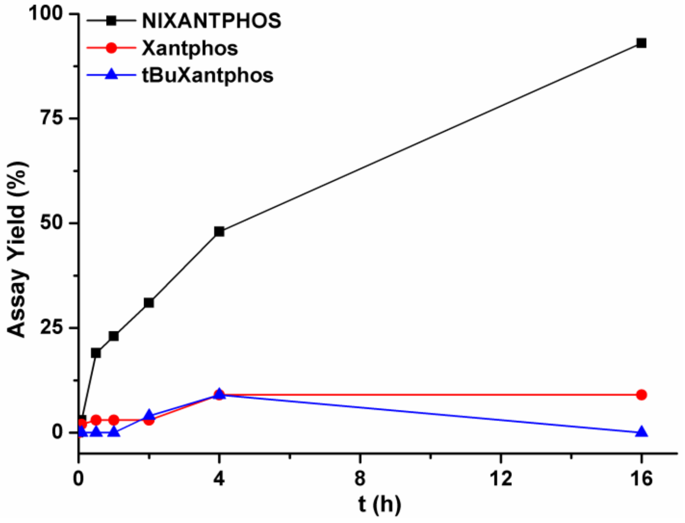

Figure S1. Assay yield at different reaction time with chlorobenzene with different ligands.

References:

1. A. M. Johns, N. Sakai, A. Ridder and J. F. Hartwig, *J. Am. Chem. Soc.*, 2006, **128,** 9306.

2. J. Zhang, A. Bellomo, A. D. Creamer, S. D. Dreher and P. J. Walsh, *J. Am. Chem. Soc.*, 2012, **134**, 13765.

3. P. D. Robinson, A. W. McLean and C. Y. Meyers, *Acta Cryst.*, 2003, **C59**, o539.

4. A. Bellomo, J. D. Zhang, N. Trongsiriwat and P. J. Walsh, *Chem. Sci.*, 2013,**4**, 849.

NMR Spectra

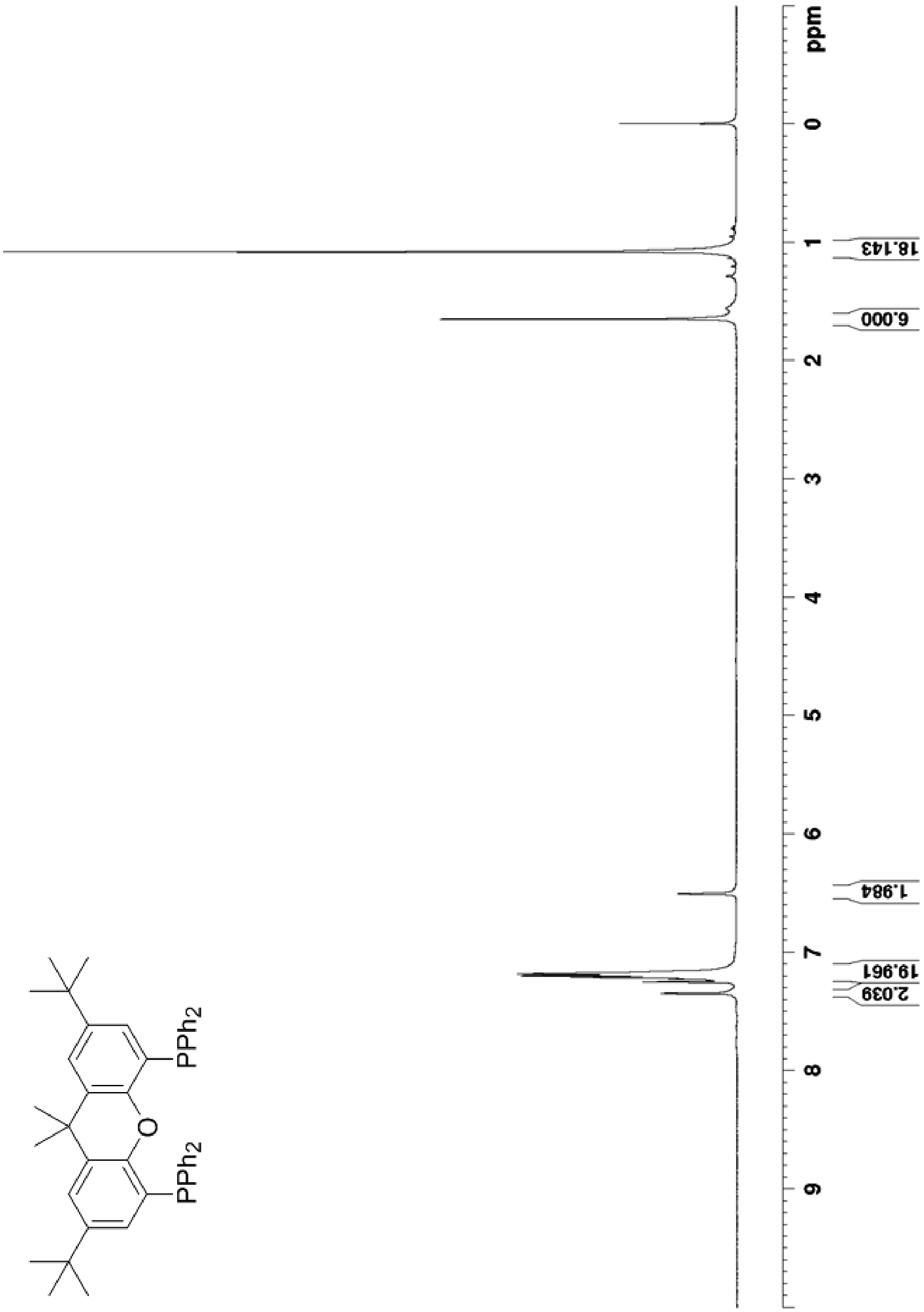

500 MHz ^{1}H NMR of ^{t}BuXANTPHOS in $CDCl_3$

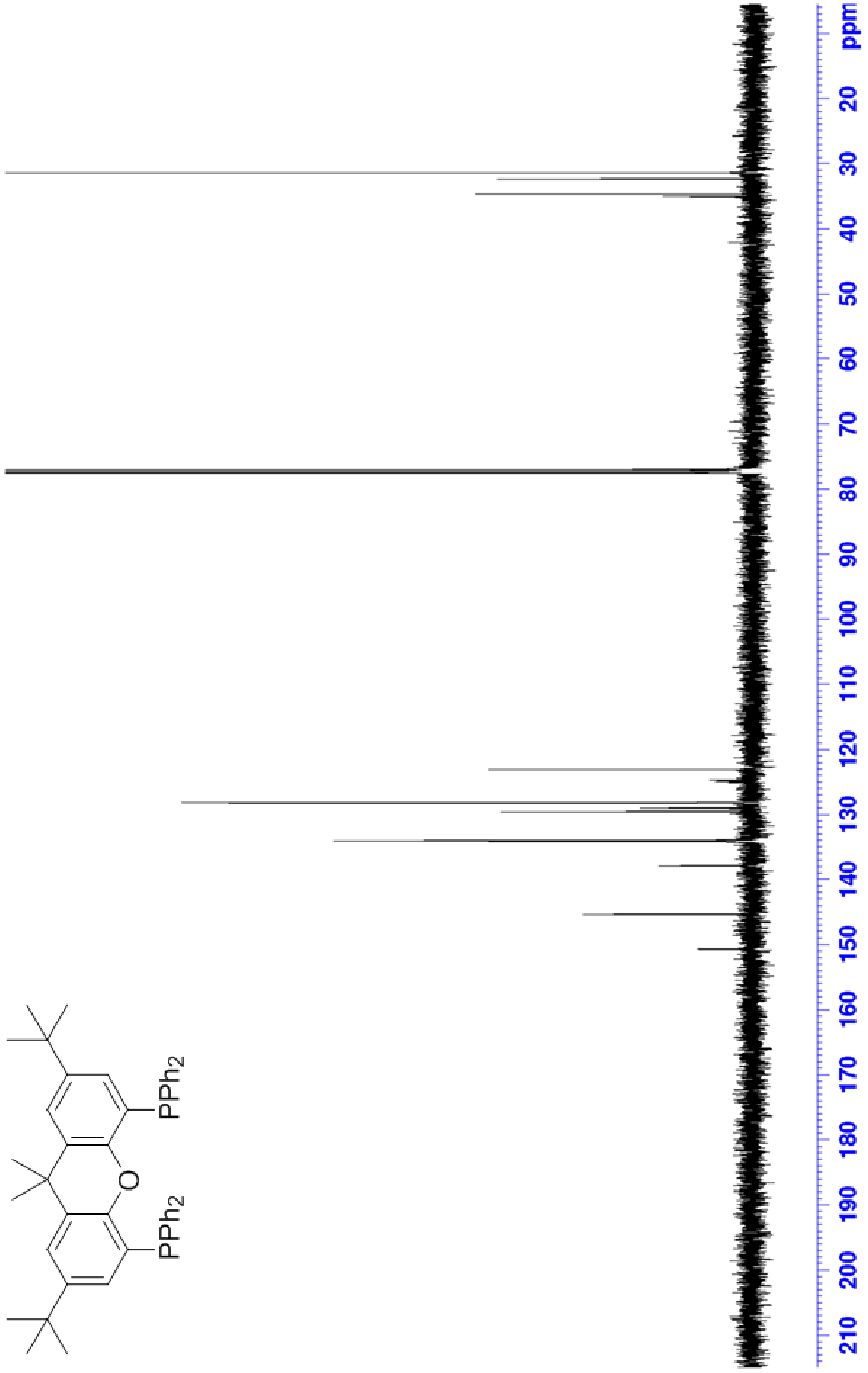

125 MHz $^{13}C\{^{1}H\}$ NMR of ^{t}BuXantphos in $CDCl_3$

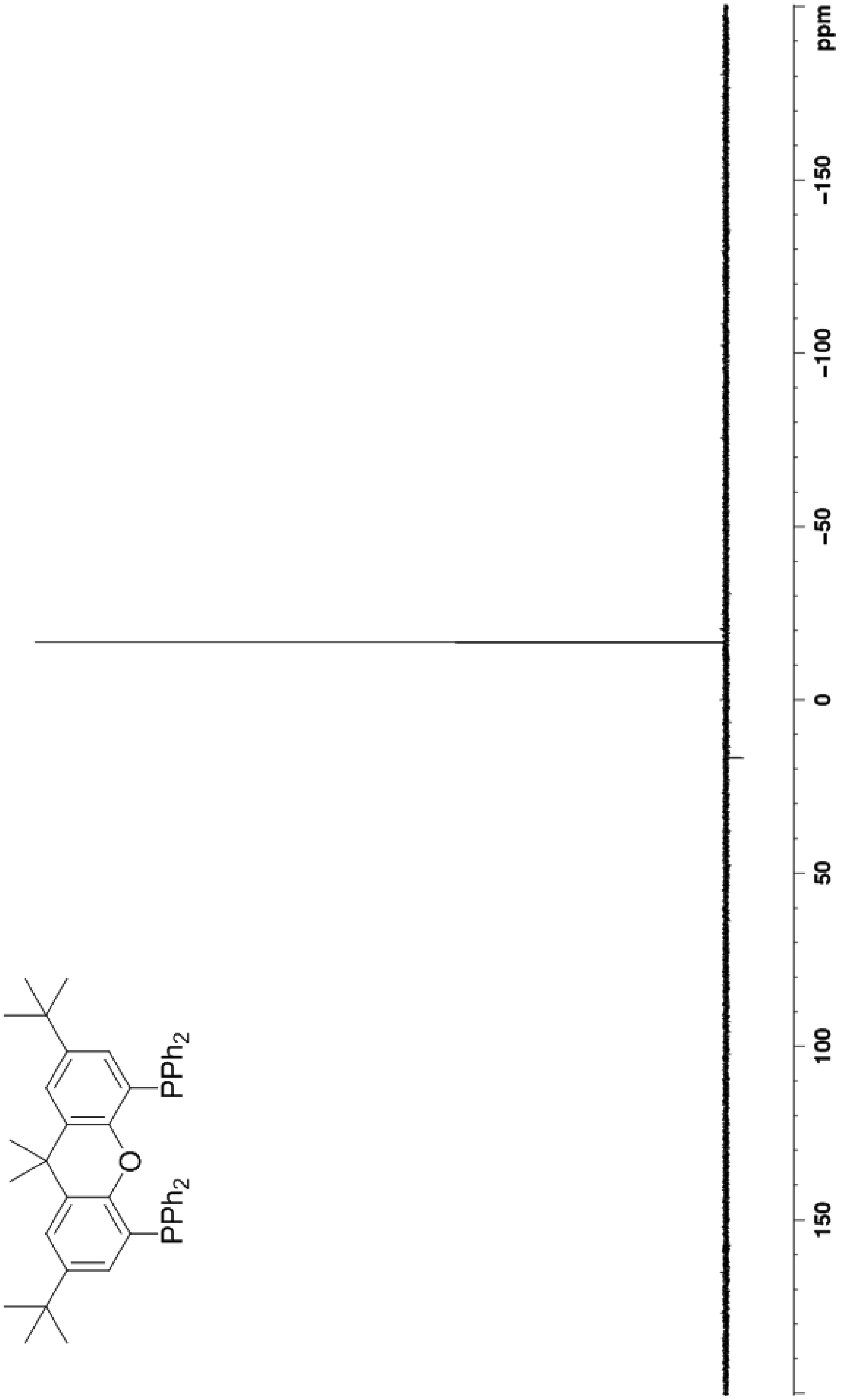

162 MHz $^{31}P\{^{1}H\}$ NMR of ^{t}BuXANTPHOS in $CDCl_3$

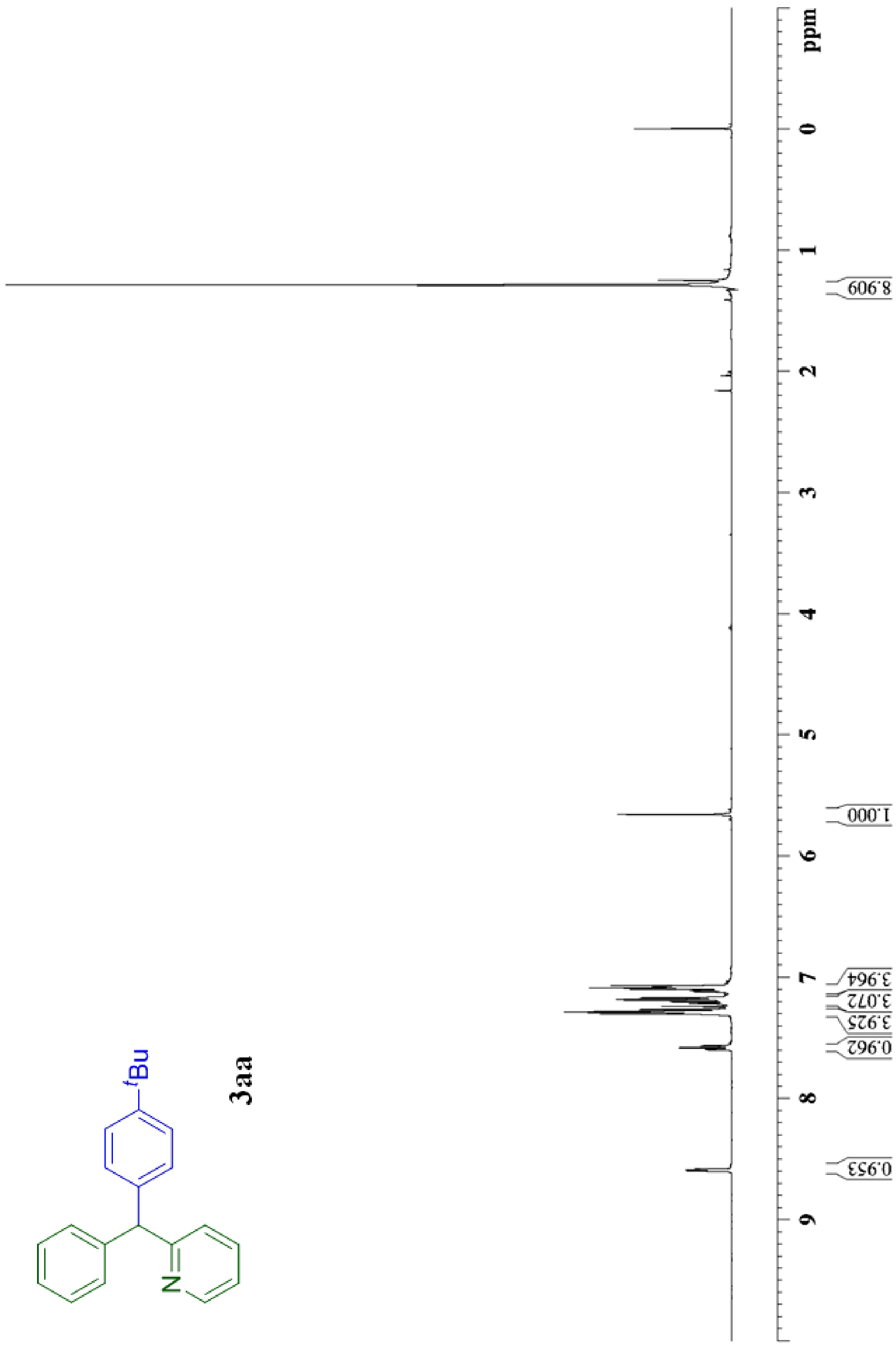

500 MHz ^{1}H NMR of **3aa** in $CDCl_3$

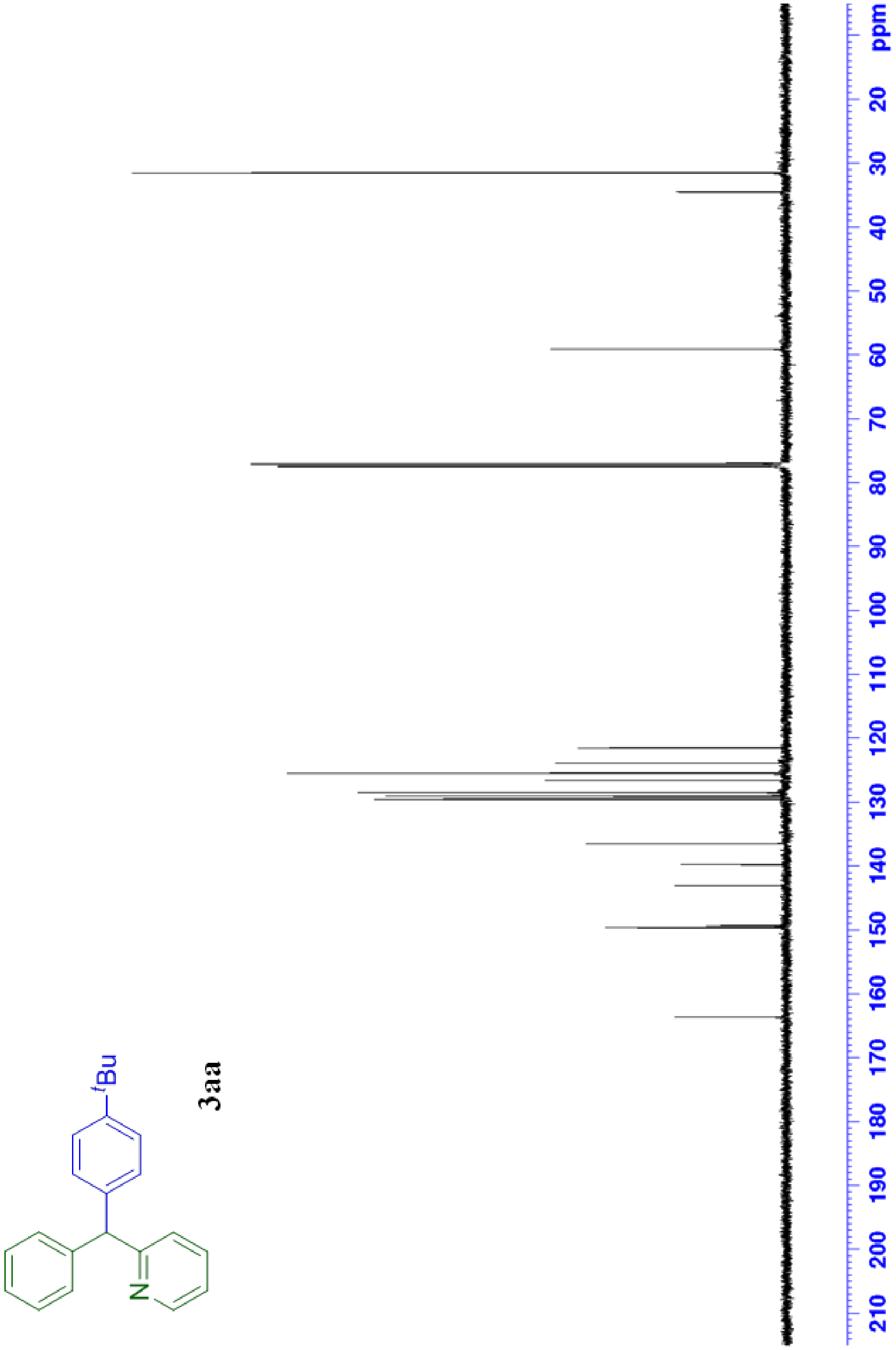

125 MHz $^{13}C\{^{1}H\}$ NMR of **3aa** in $CDCl_3$

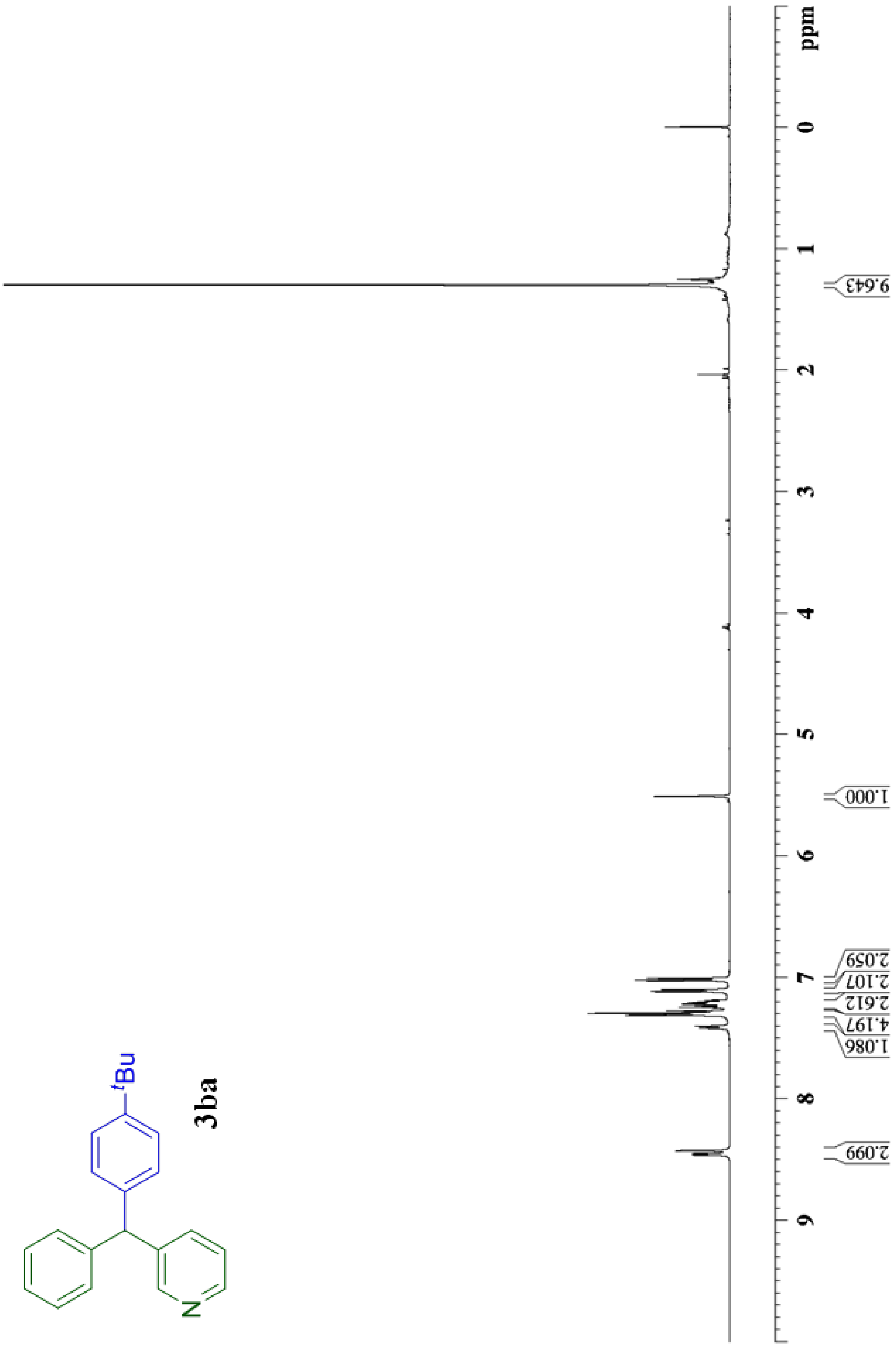

500 MHz ^{1}H NMR of **3ba** in $CDCl_3$

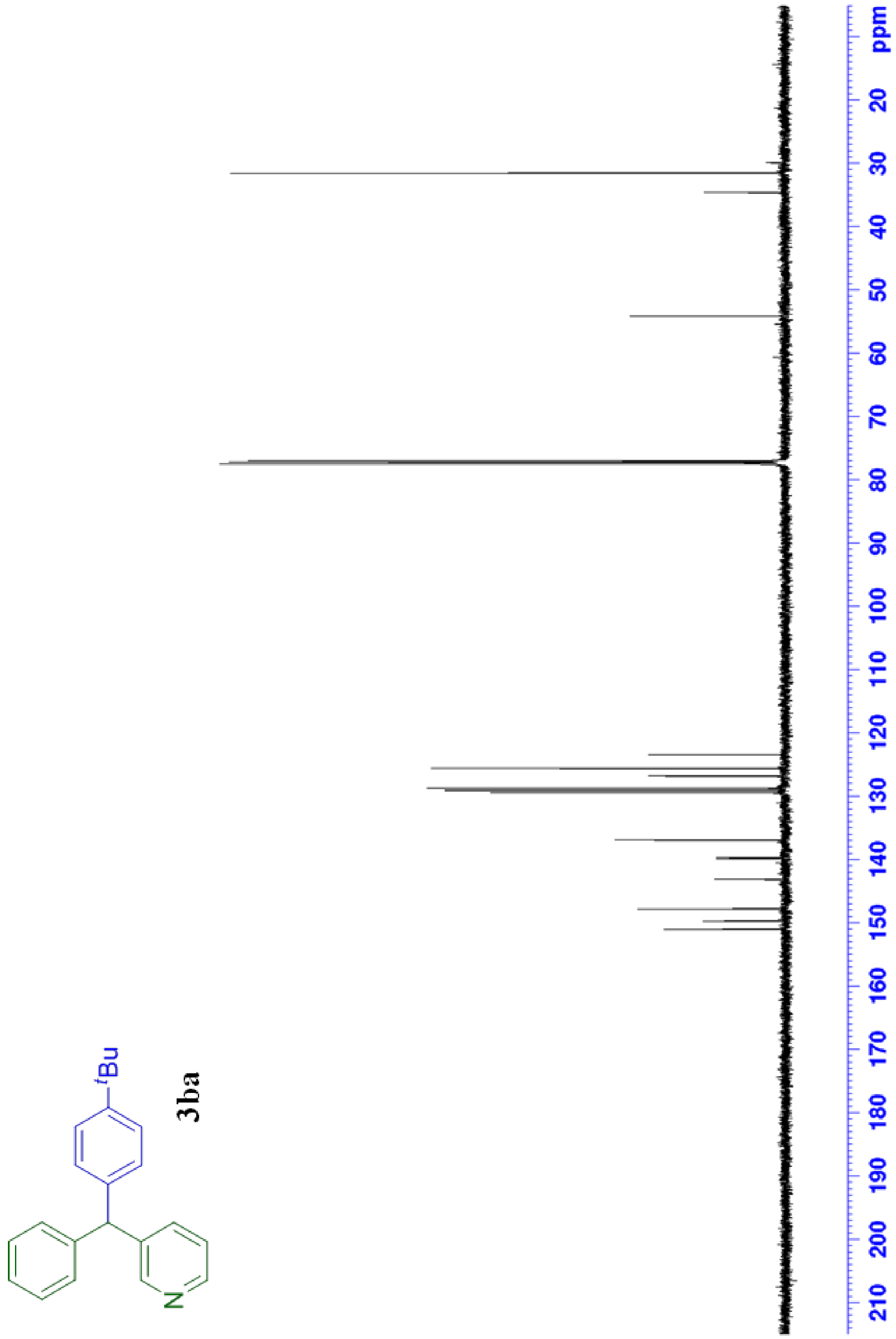

125 MHz $^{13}C\{^1H\}$ NMR of **3ba** in $CDCl_3$

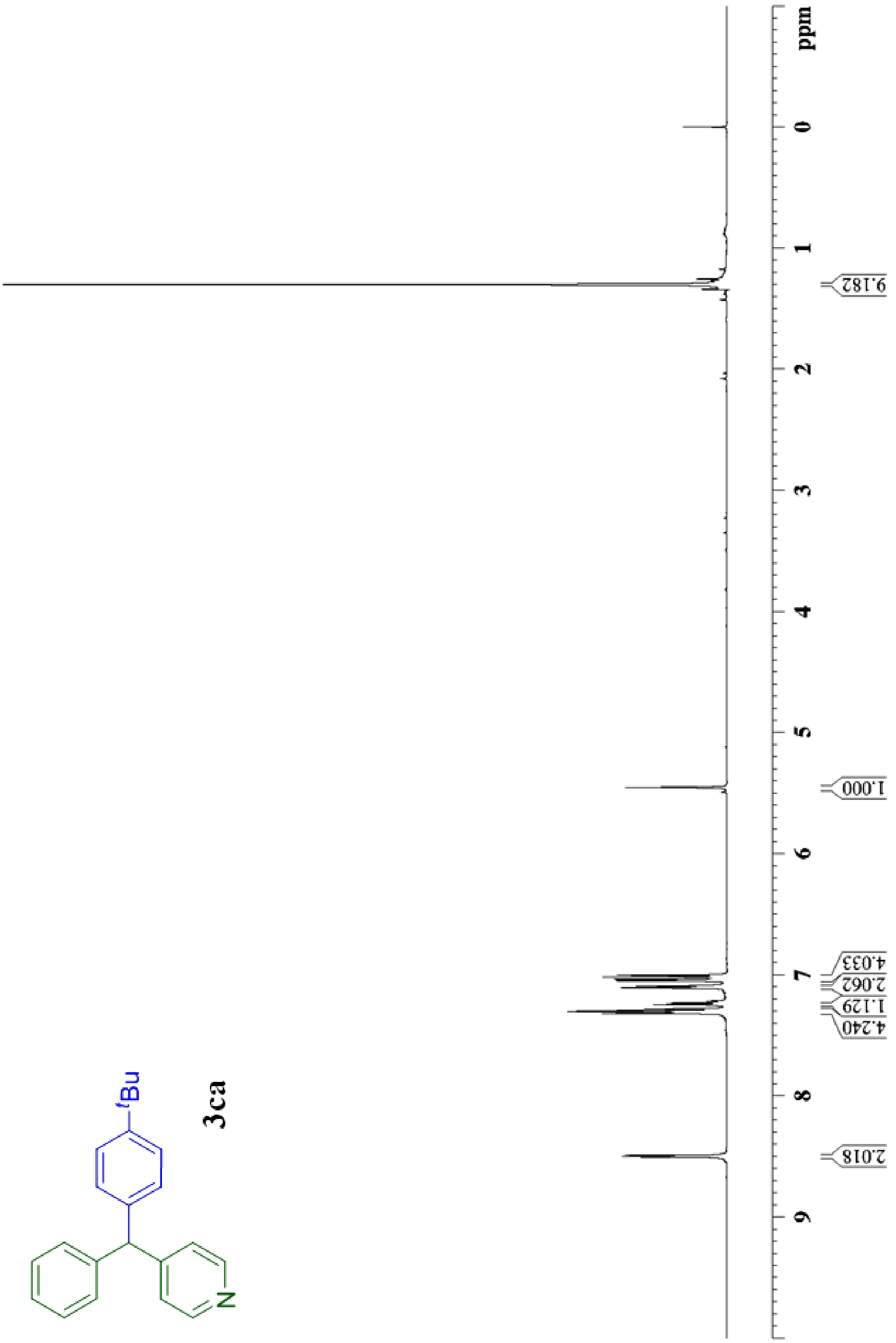

500 MHz ^{1}H NMR of **3ca** in $CDCl_3$

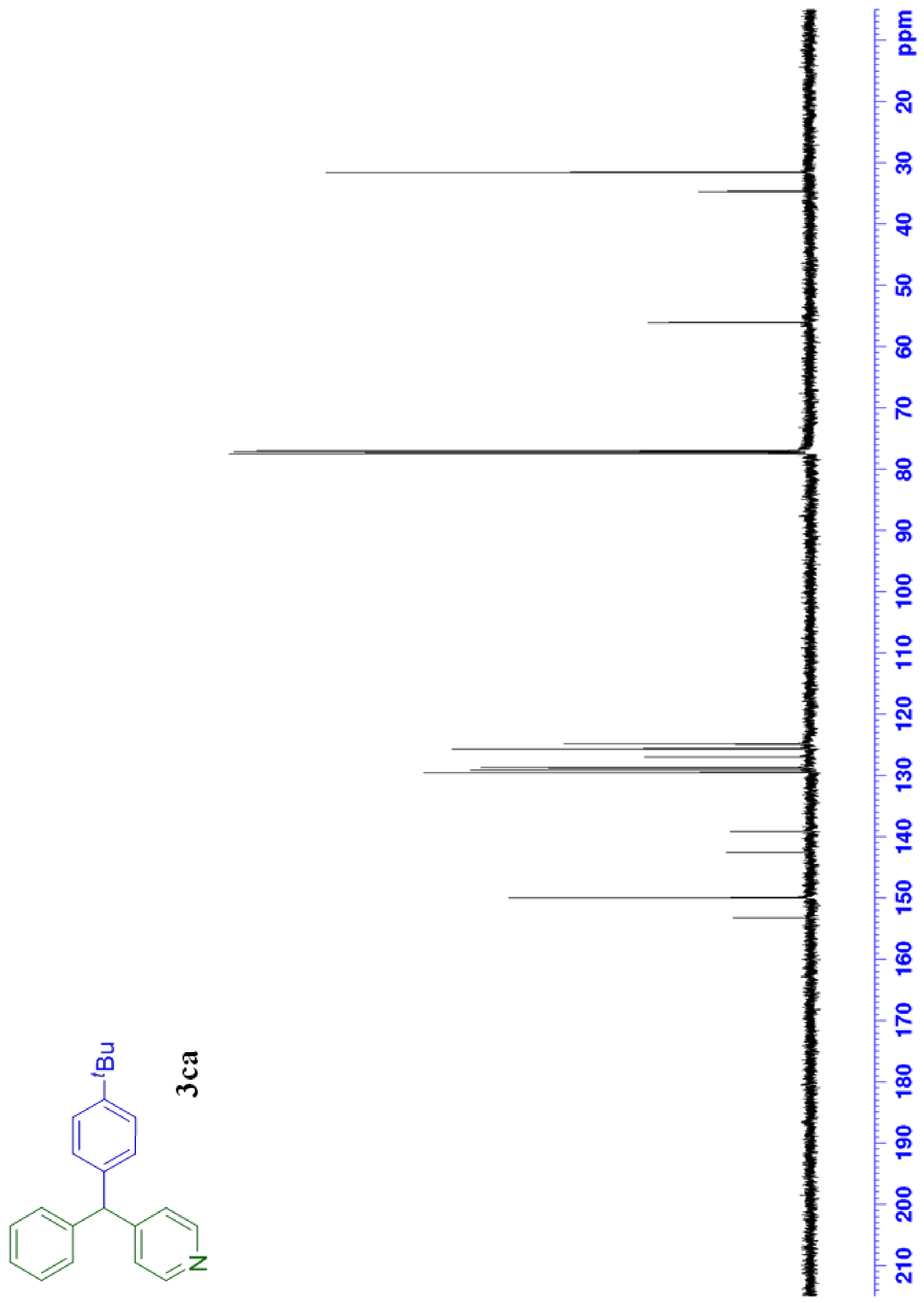

125 MHz $^{13}C\{^{1}H\}$ NMR of **3ca** in $CDCl_3$

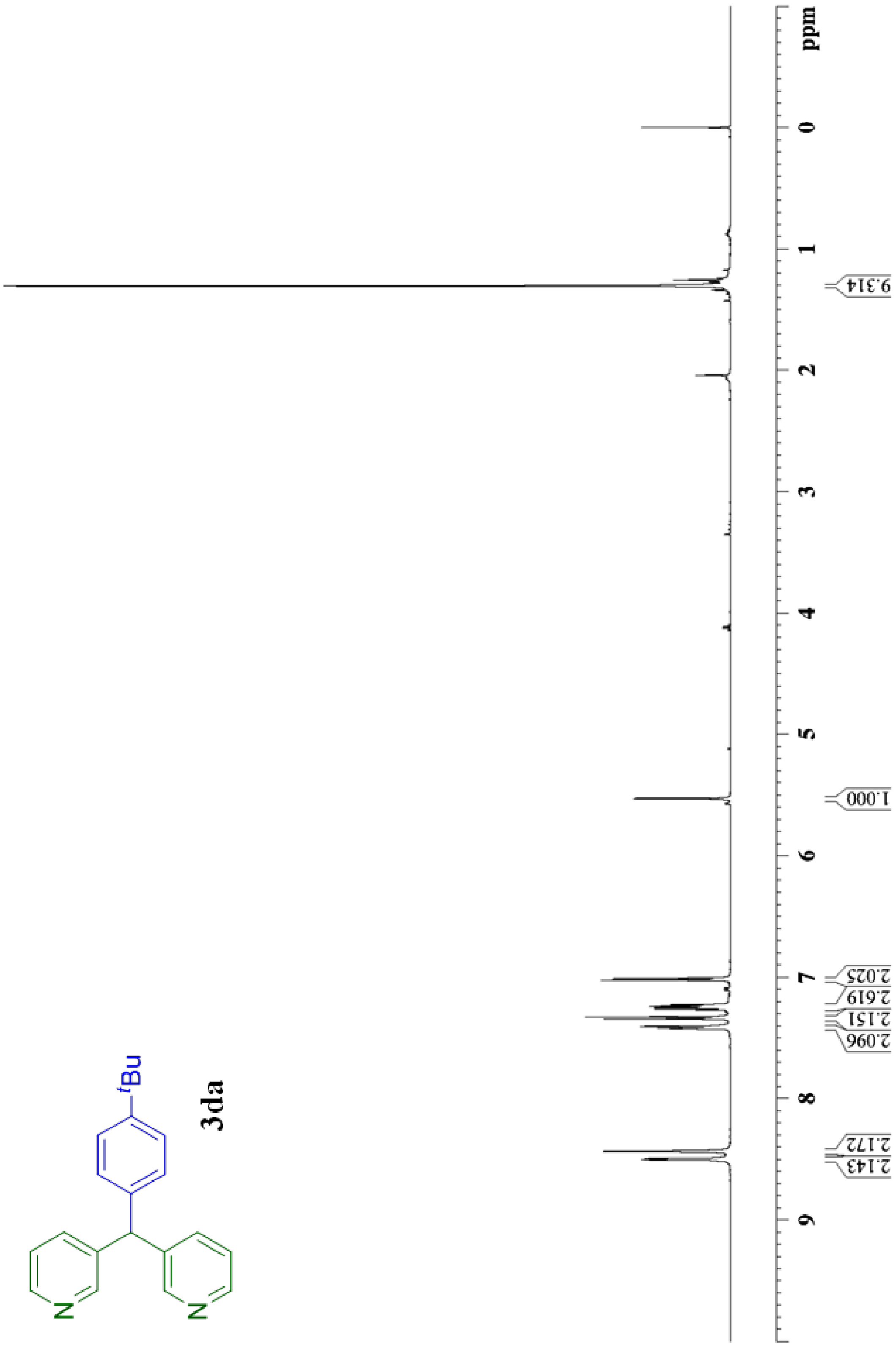

500 MHz ^{1}H NMR of **3da** in $CDCl_3$

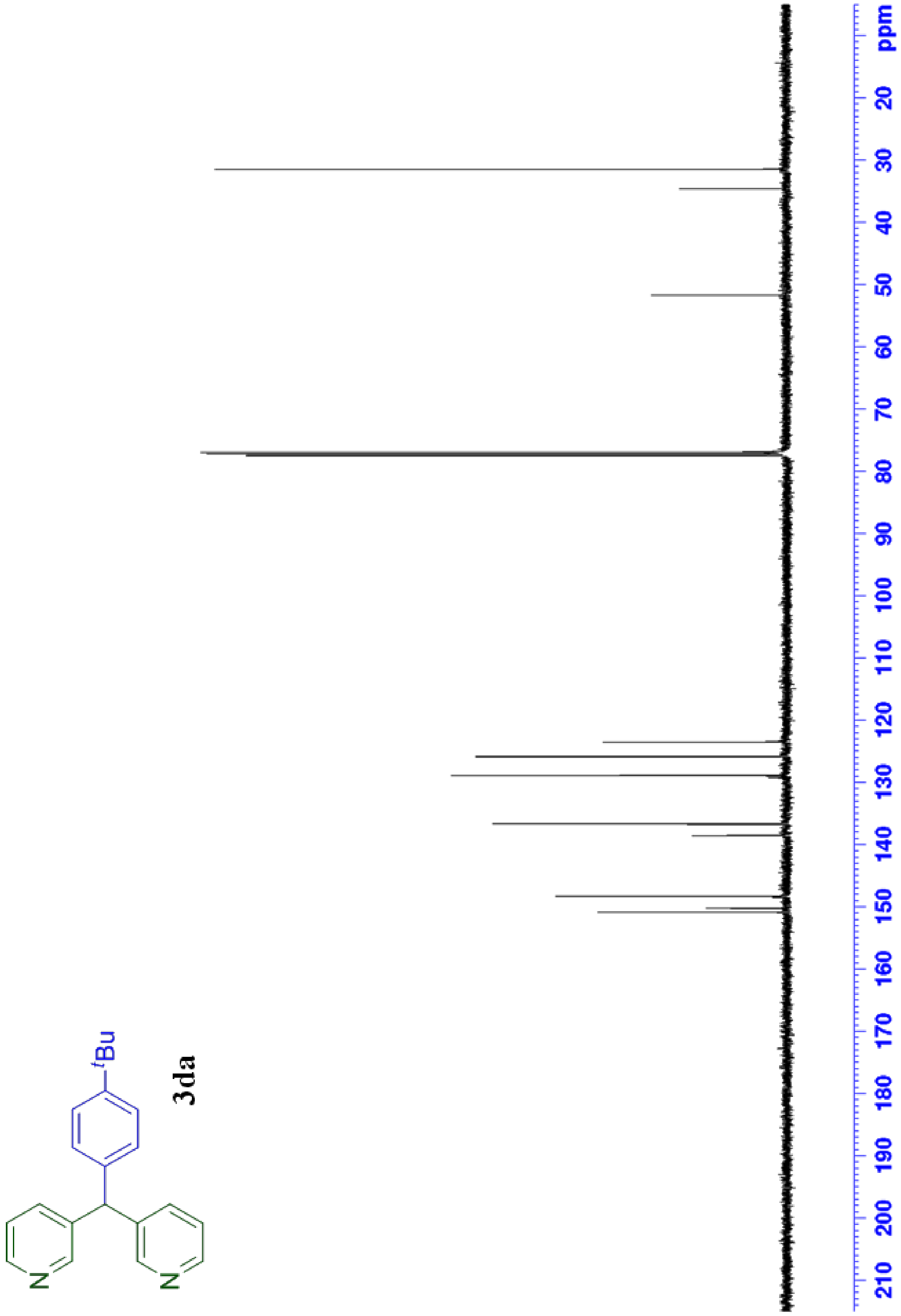

125 MHz $^{13}C\{^{1}H\}$ NMR of **3da** in $CDCl_3$

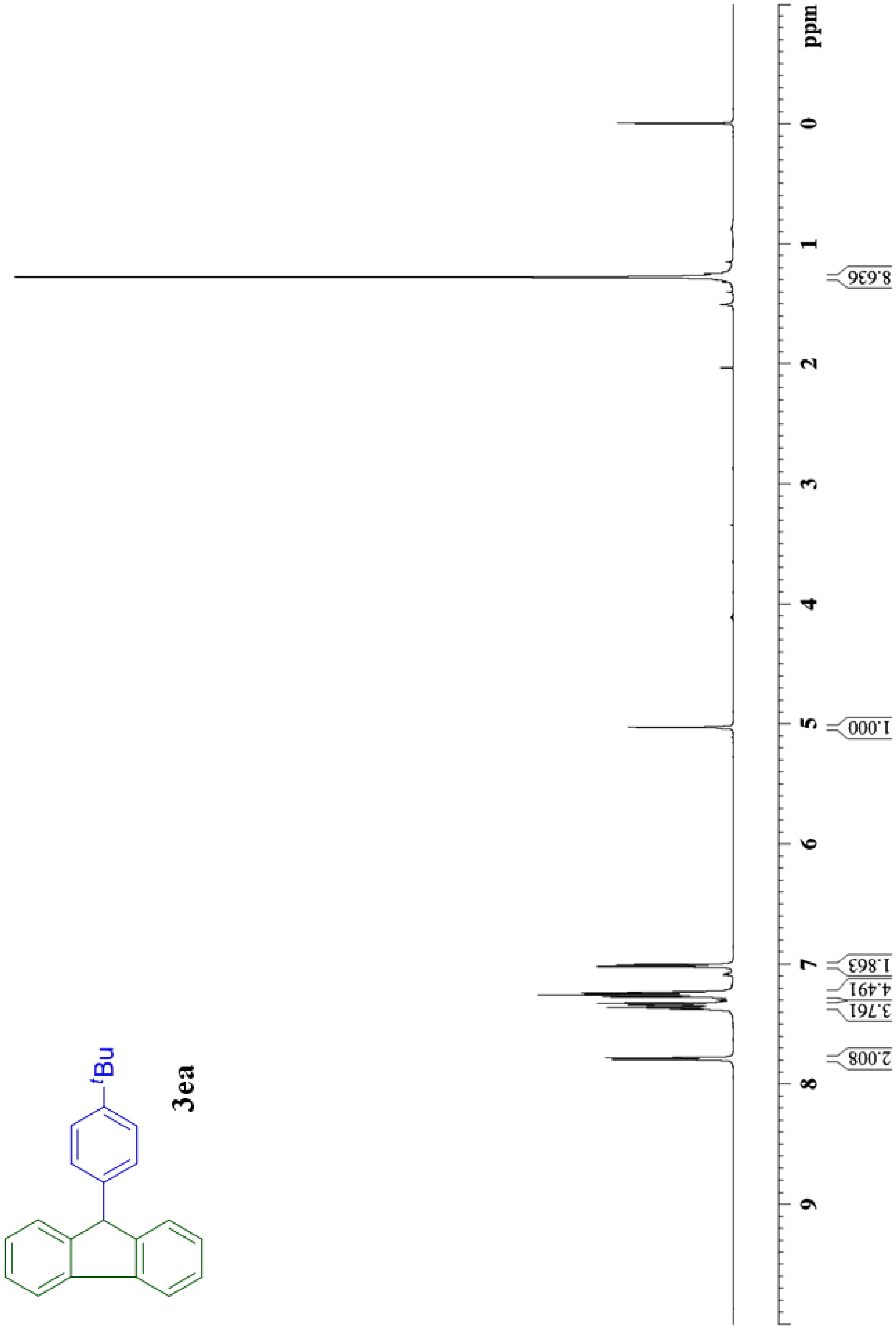

500 MHz ^{1}H NMR of **3ea** in $CDCl_3$

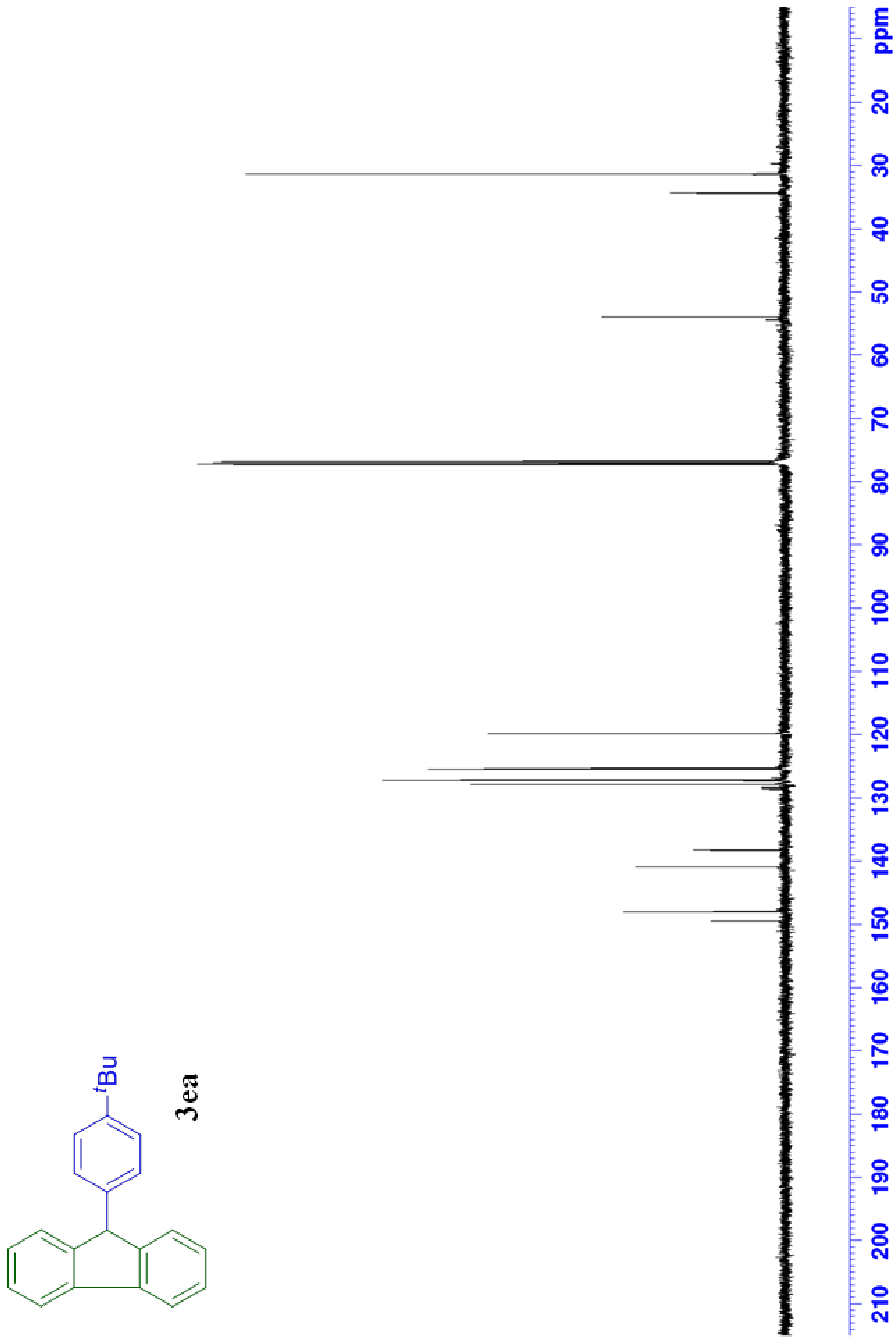

125 MHz $^{13}C\{^1H\}$ NMR of **3ea** in $CDCl_3$

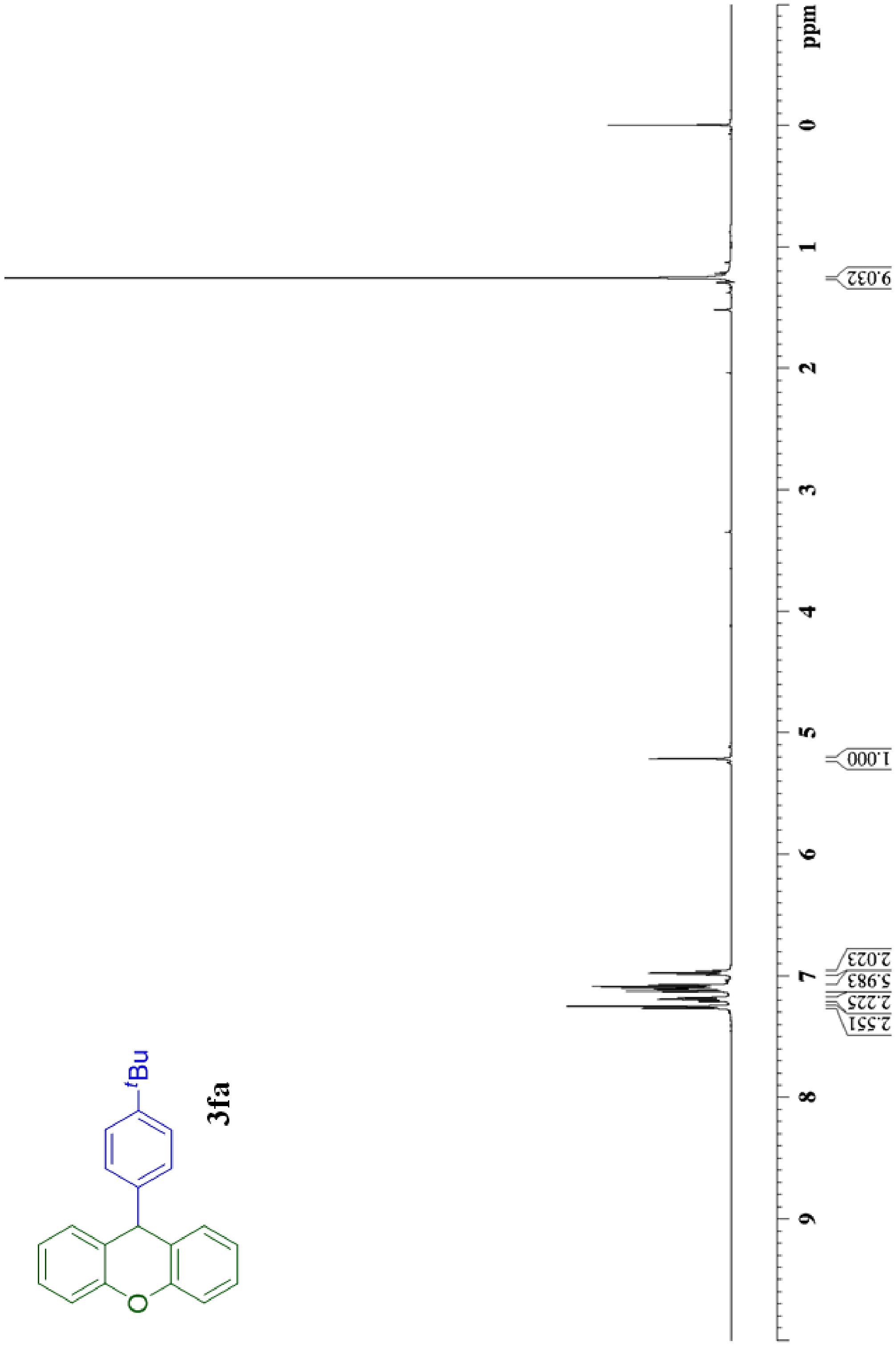

500 MHz ^{1}H NMR of **3fa** in $CDCl_3$

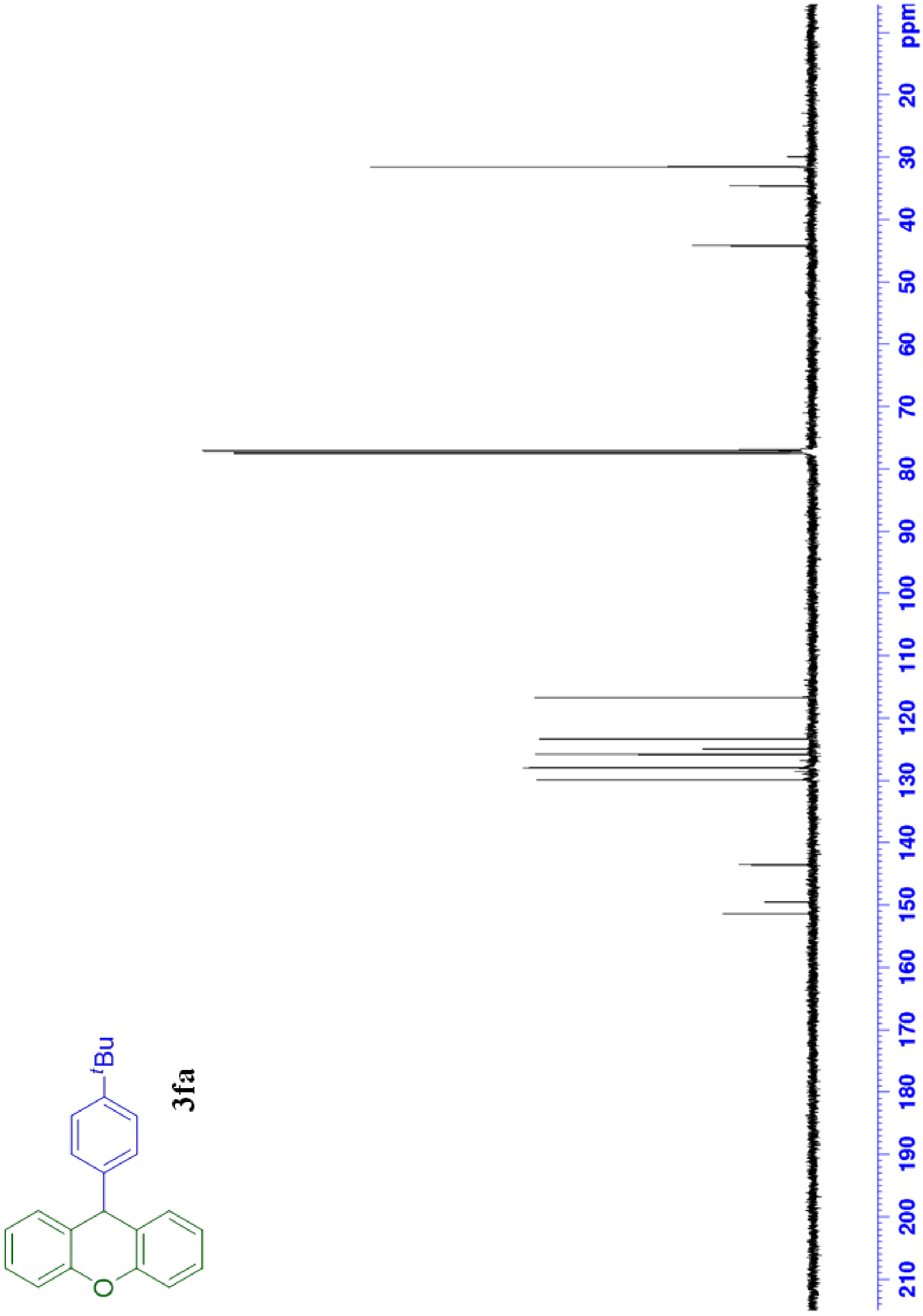

125 MHz $^{13}C\{^{1}H\}$ NMR of **3fa** in $CDCl_3$

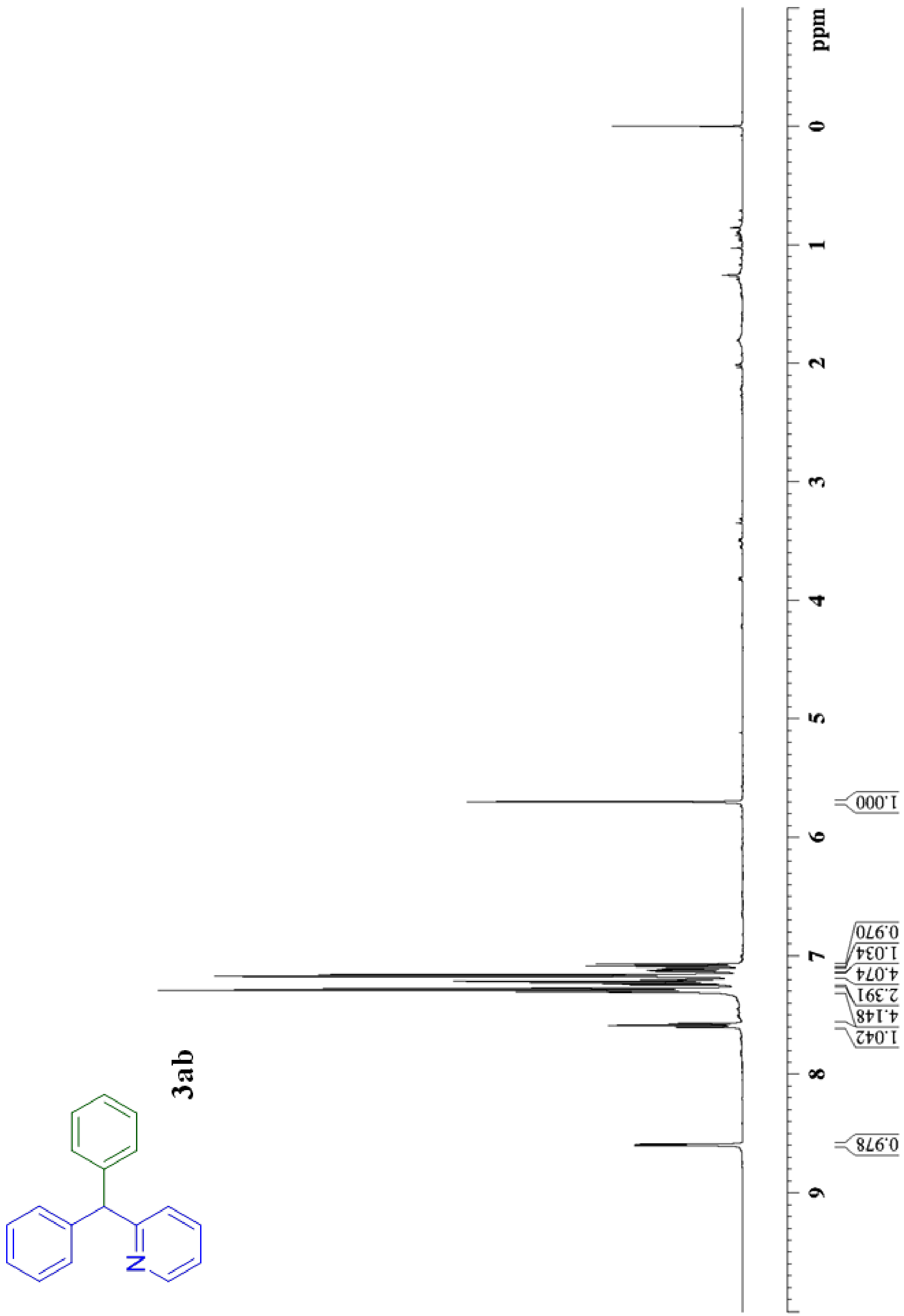

500 MHz ^{1}H NMR of **3ab** in $CDCl_3$

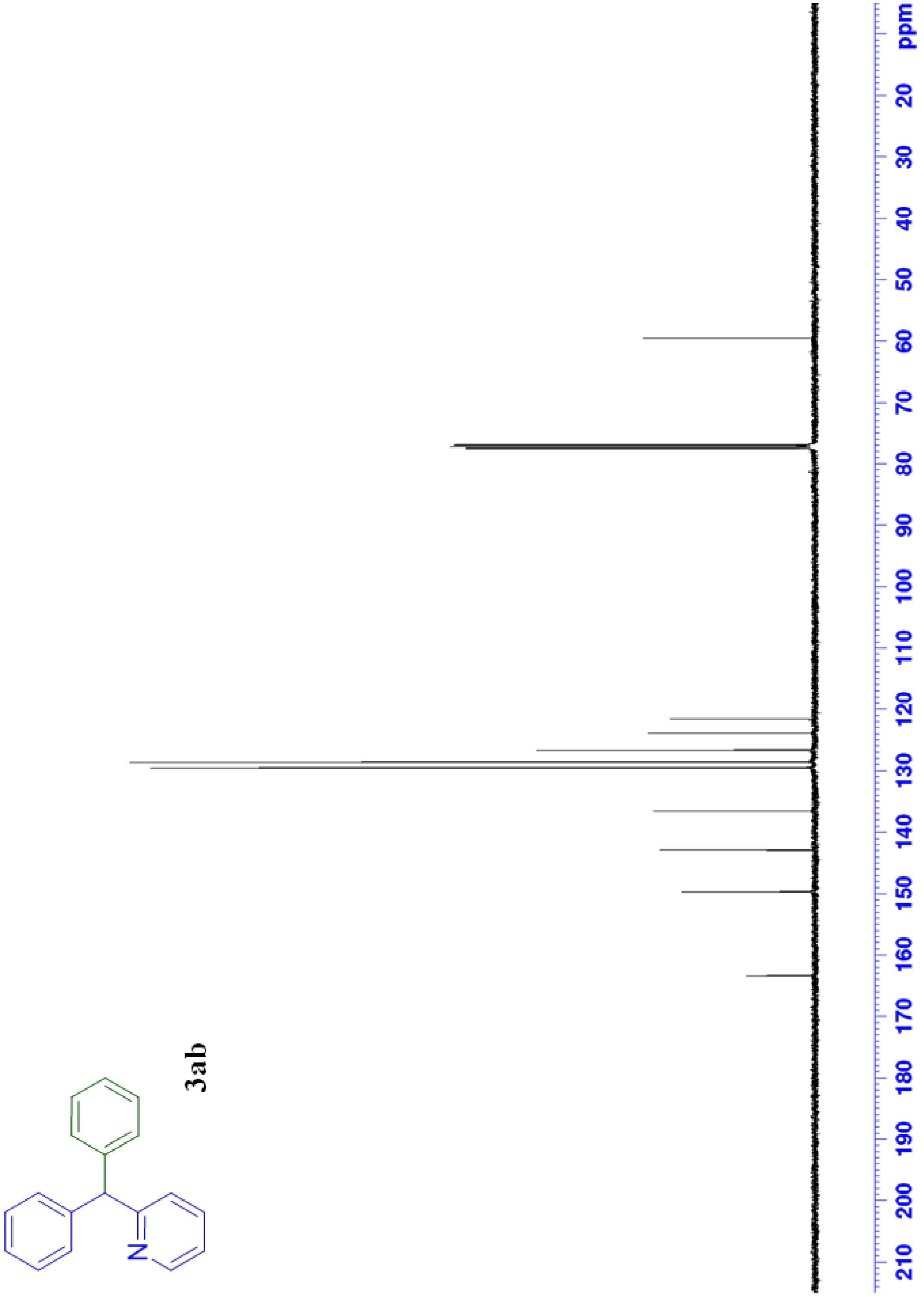

125 MHz $^{13}C\{^{1}H\}$ NMR of **3ab** in $CDCl_3$

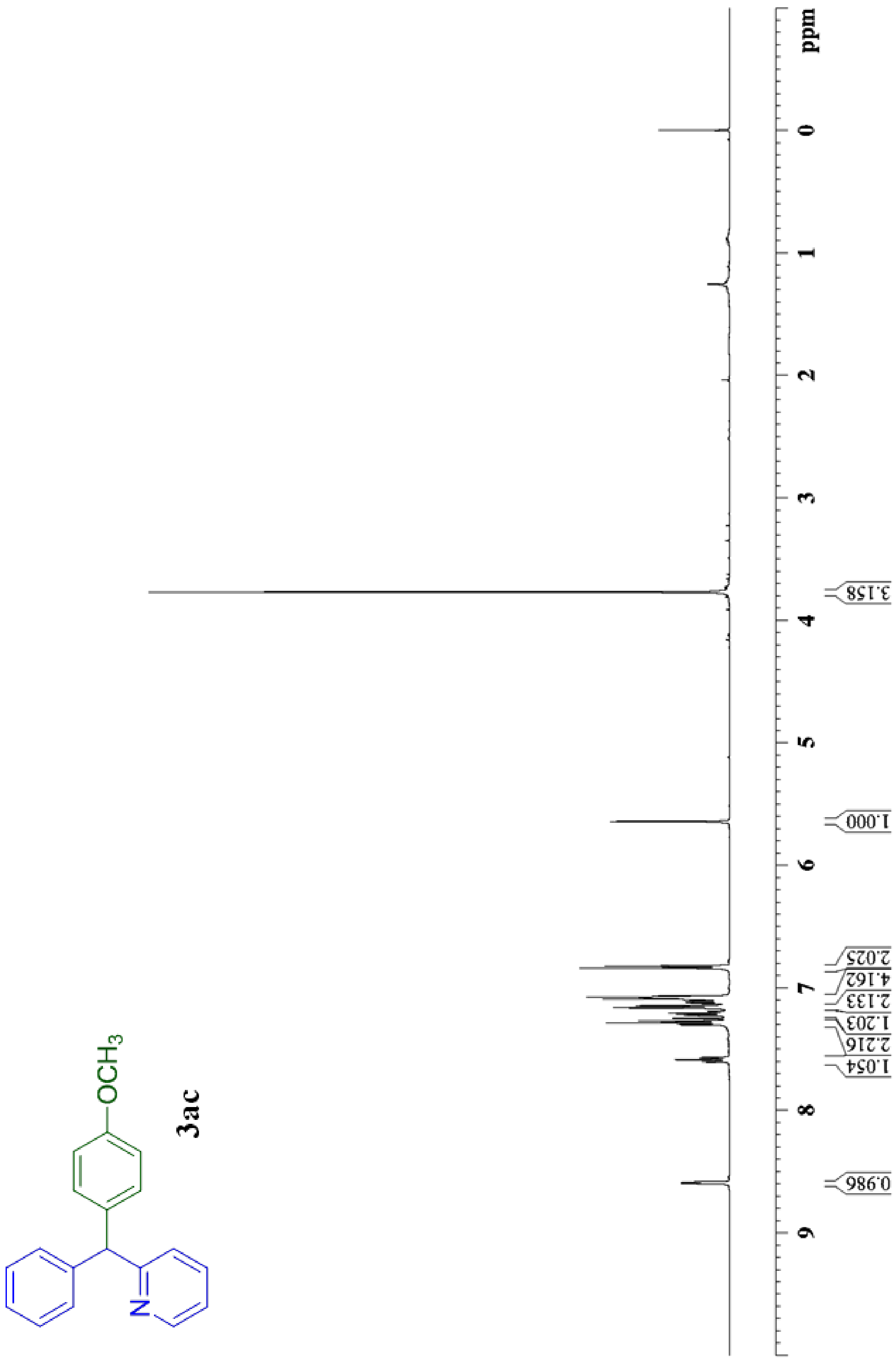

500 MHz ^{1}H NMR of **3ac** in $CDCl_3$

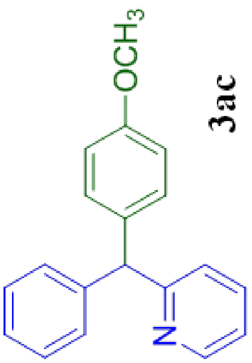

3ac

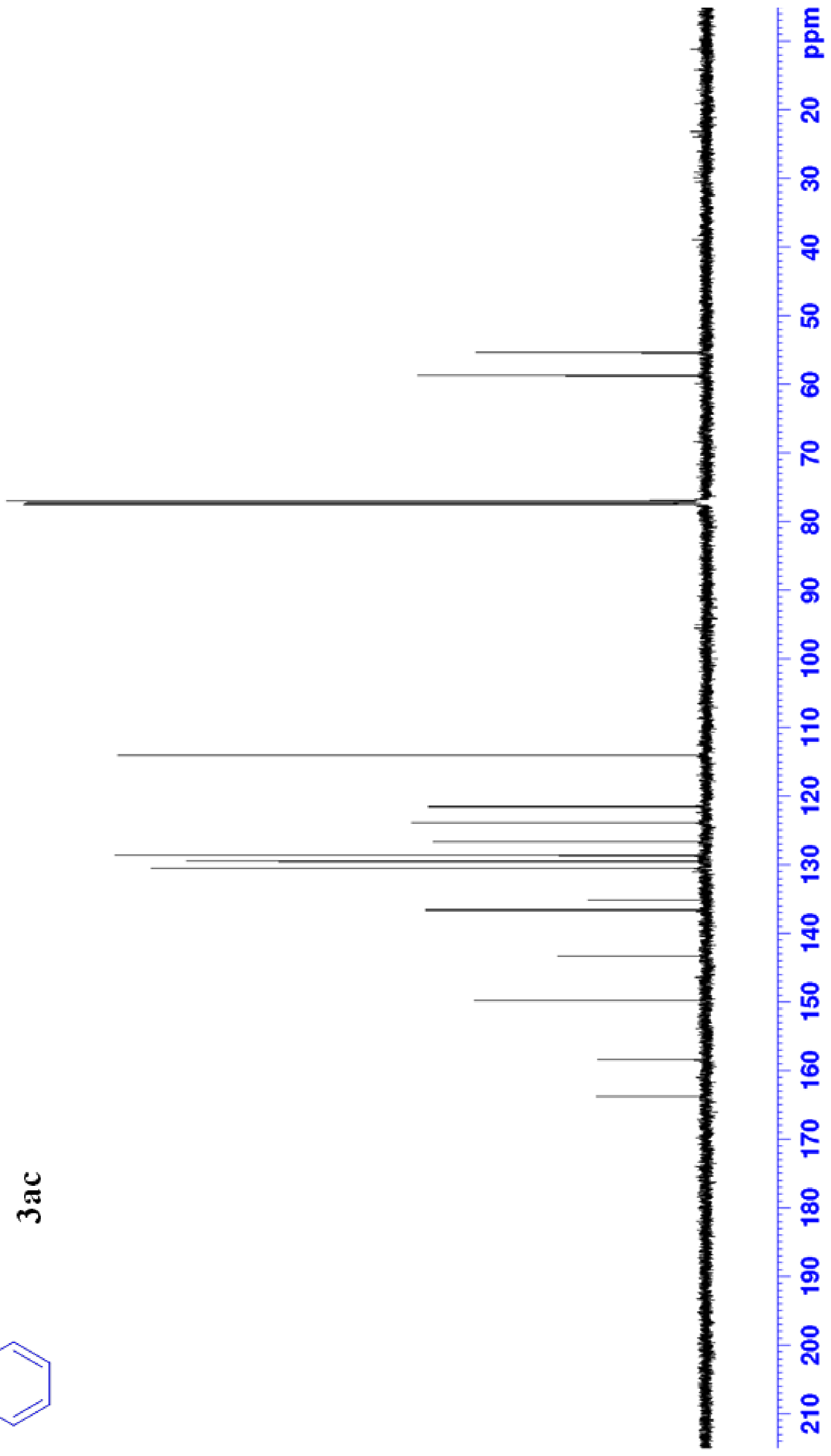

125 MHz $^{13}C\{^{1}H\}$ NMR of **3ac** in $CDCl_3$

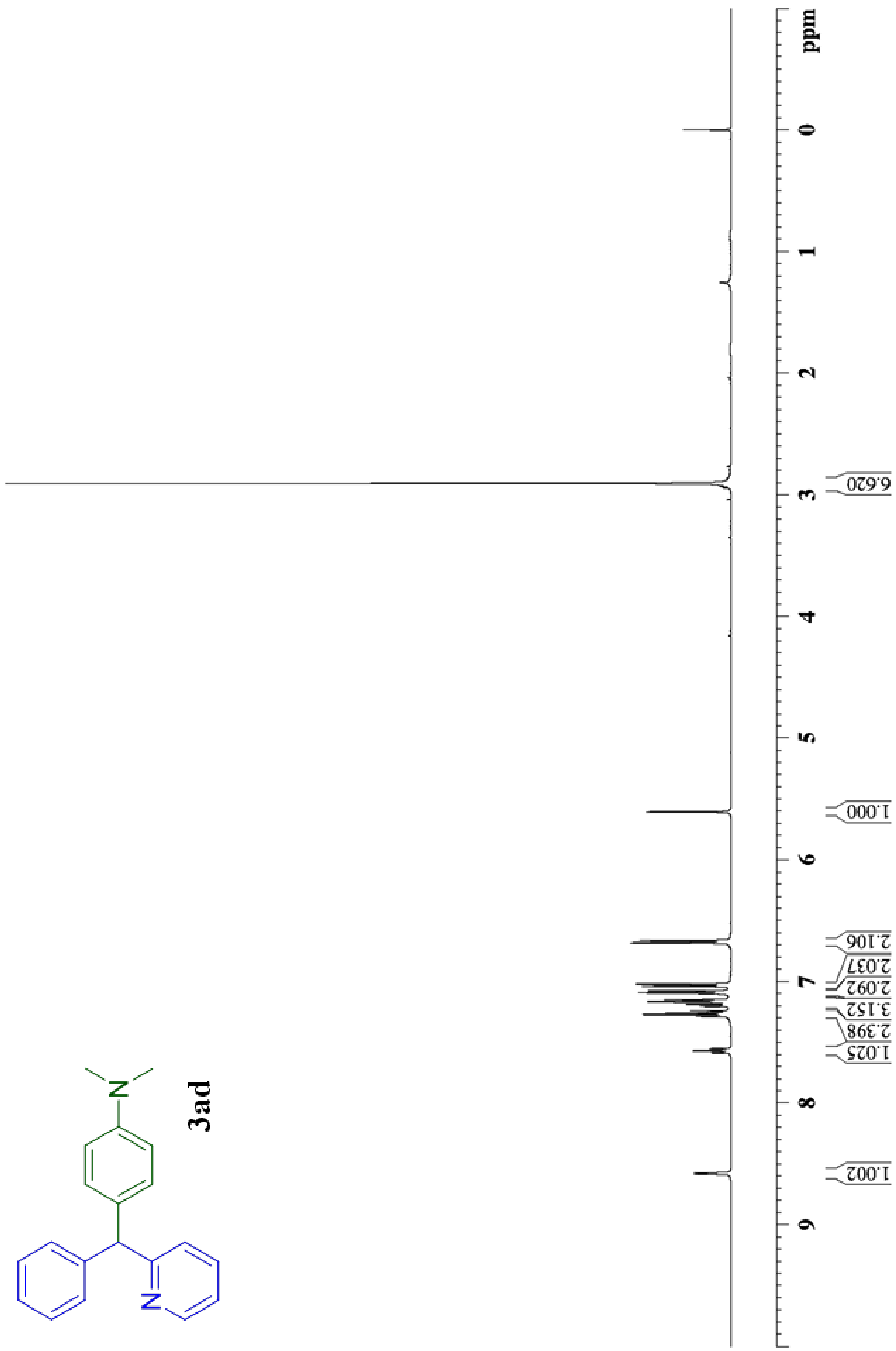

500 MHz ^{1}H NMR of **3ad** in $CDCl_3$

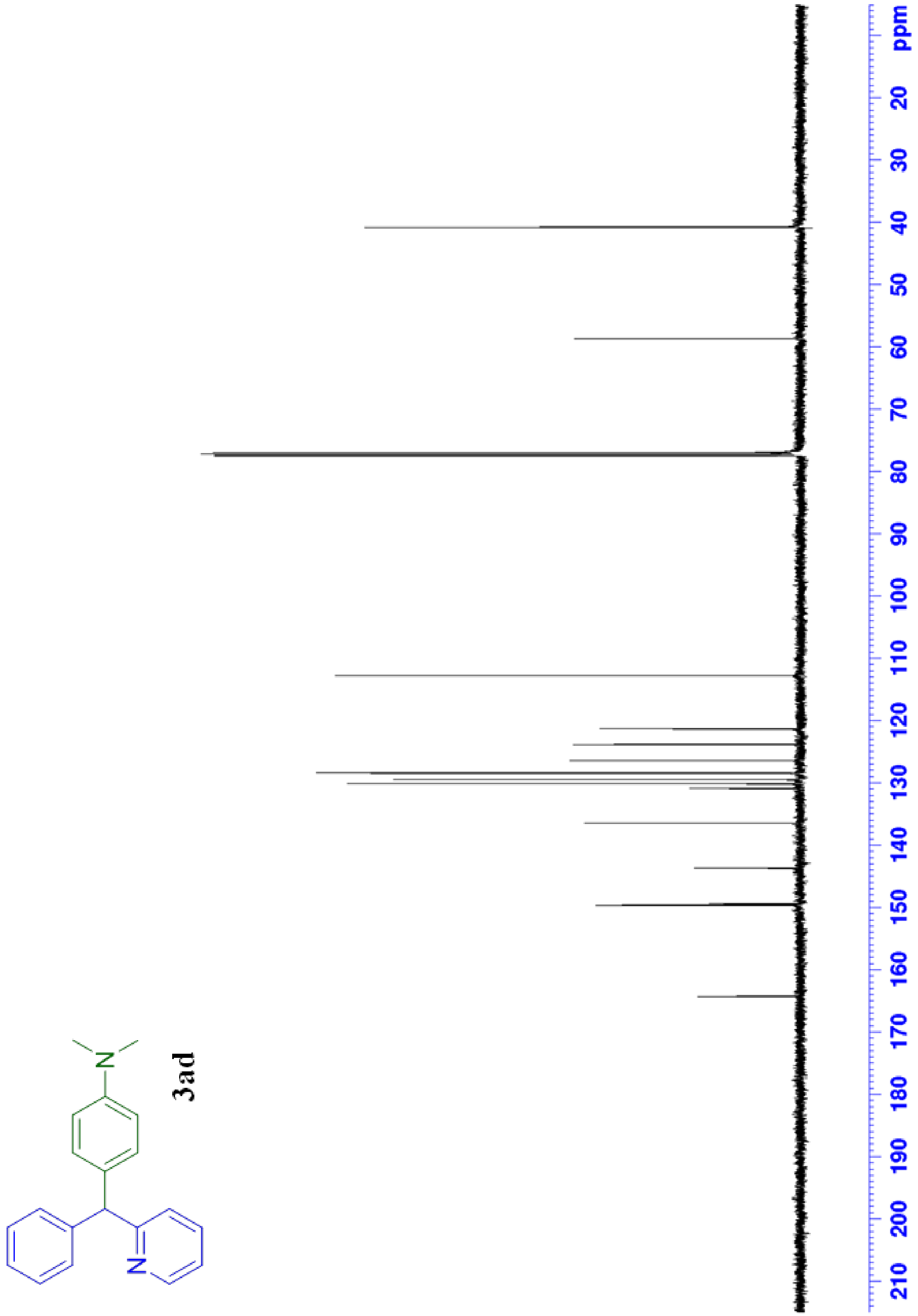

125 MHz $^{13}C\{^{1}H\}$ NMR of **3ad** in $CDCl_3$

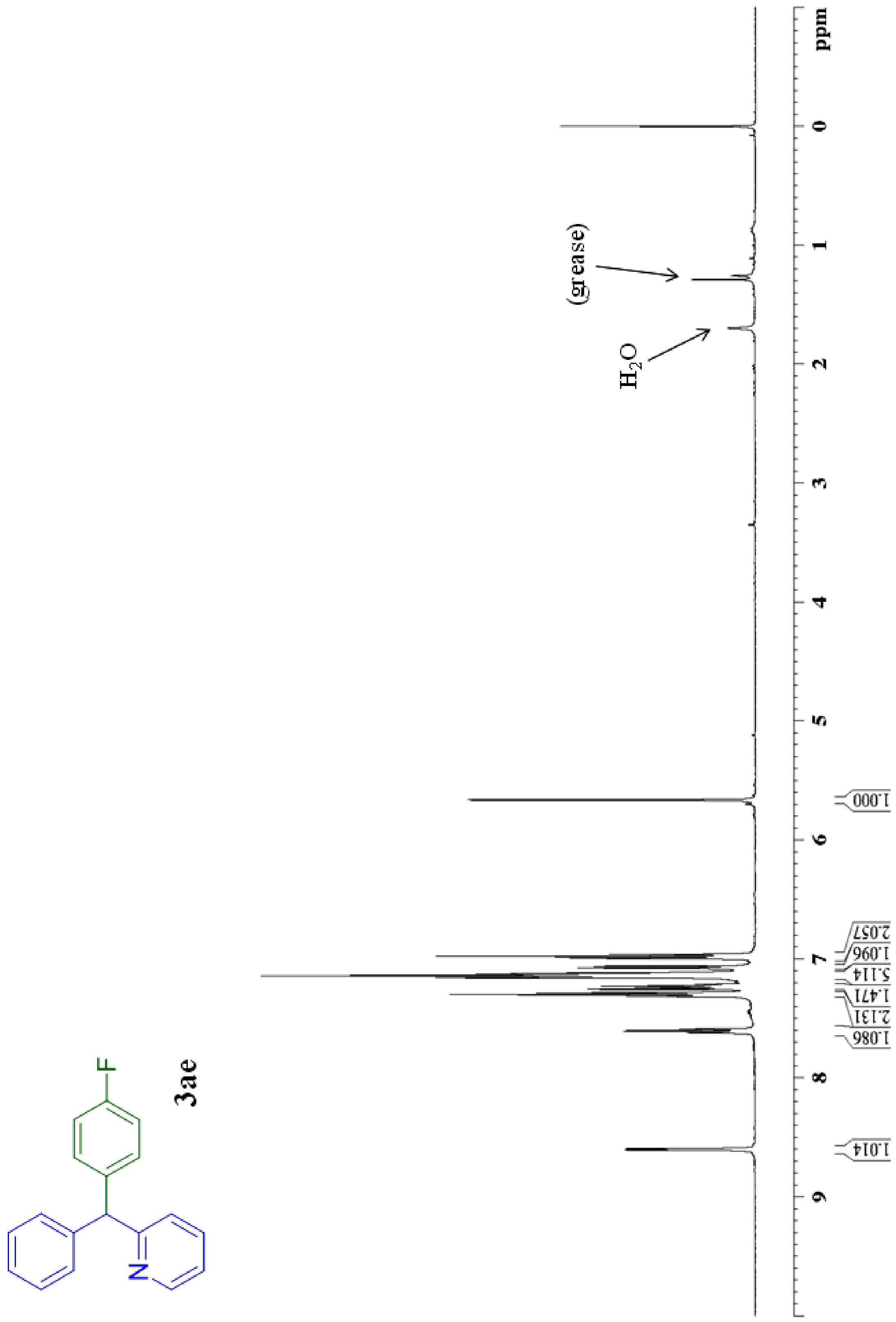

500 MHz ^{1}H NMR of **3ae** in $CDCl_3$

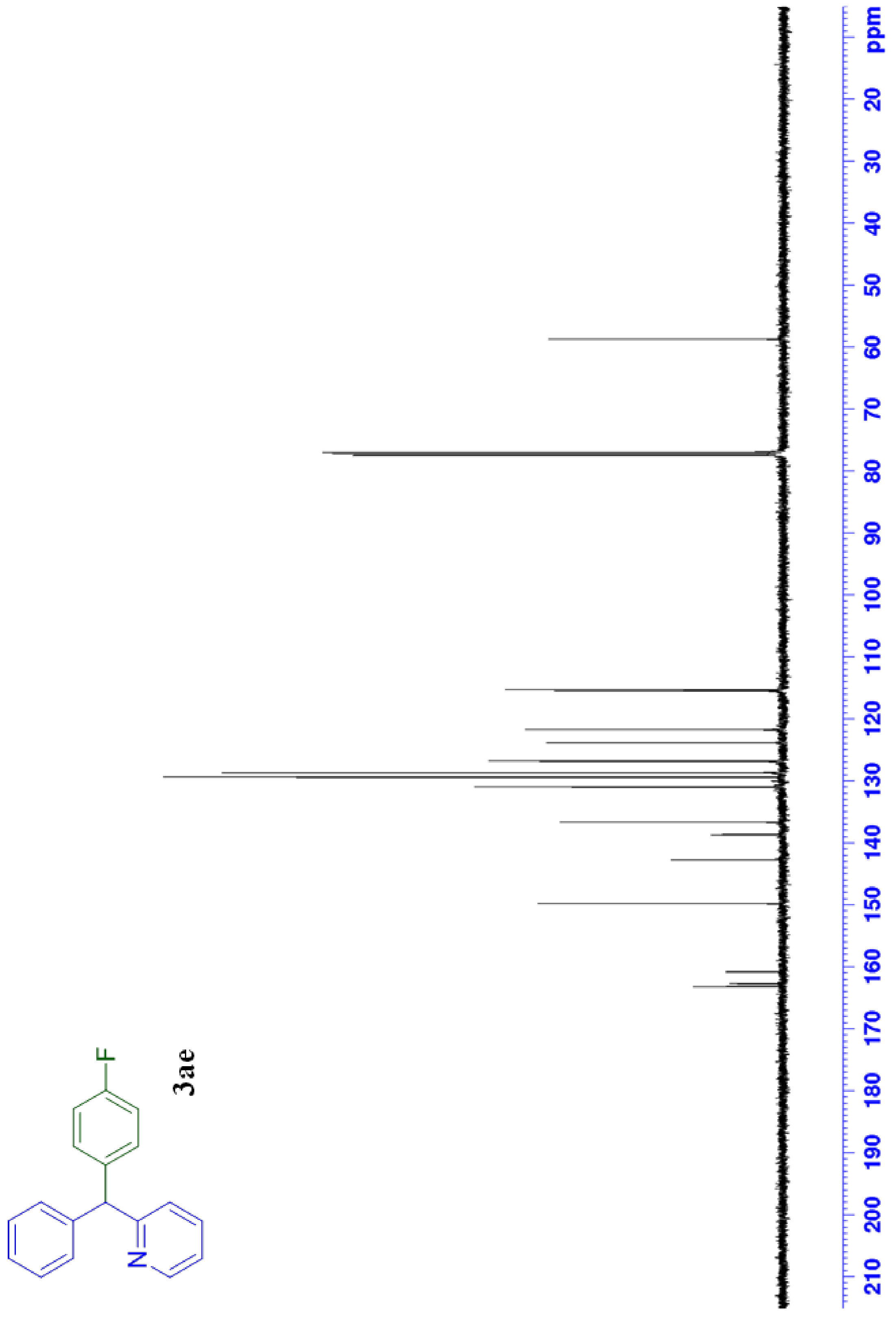

125 MHz $^{13}C\{^{1}H\}$ NMR of **3ae** in $CDCl_3$

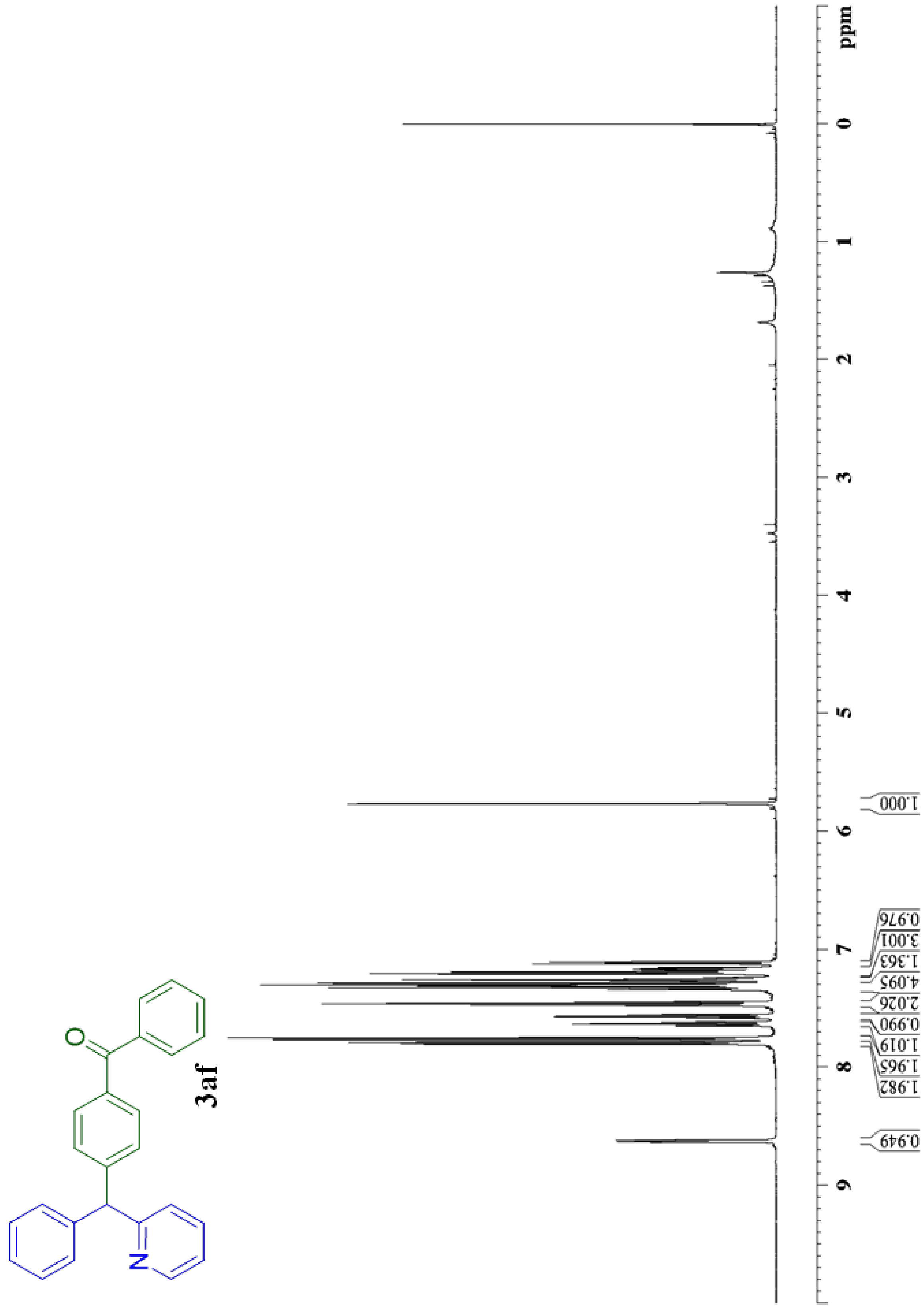

500 MHz ^{1}H NMR of **3af** in $CDCl_3$

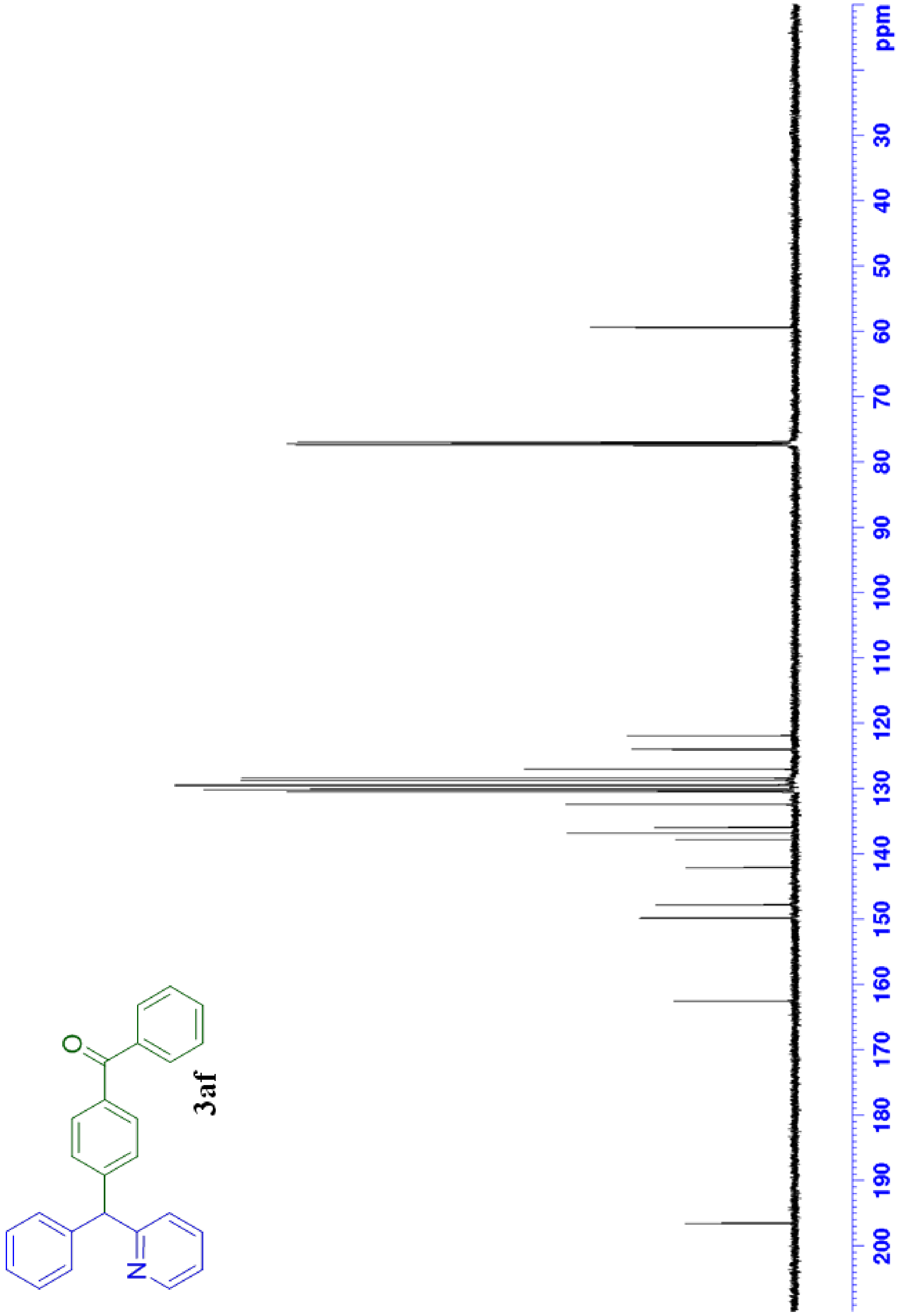

125 MHz $^{13}C\{^{1}H\}$ NMR of **3af** in $CDCl_3$

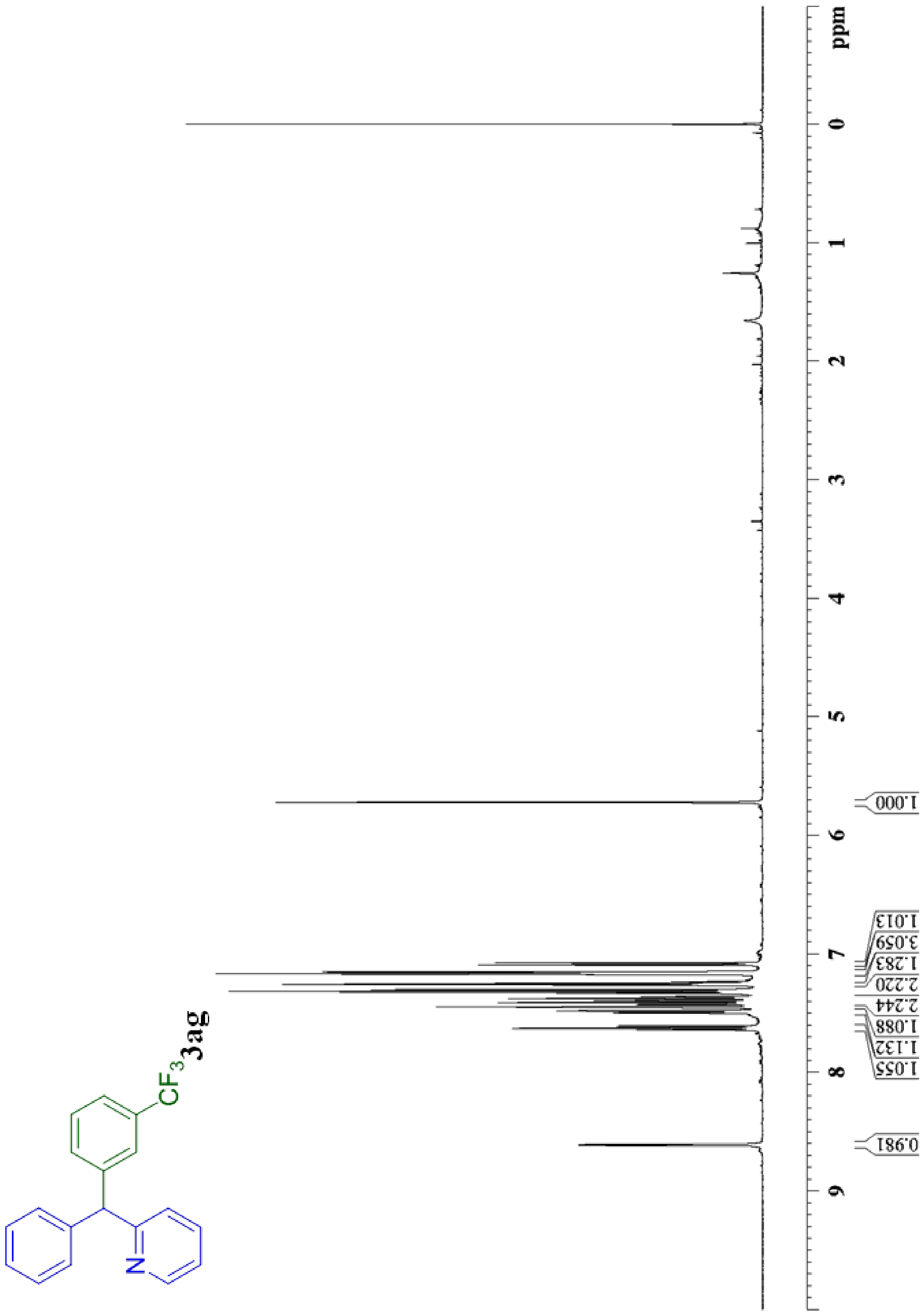

500 MHz ^{1}H NMR of **3ag** in $CDCl_3$

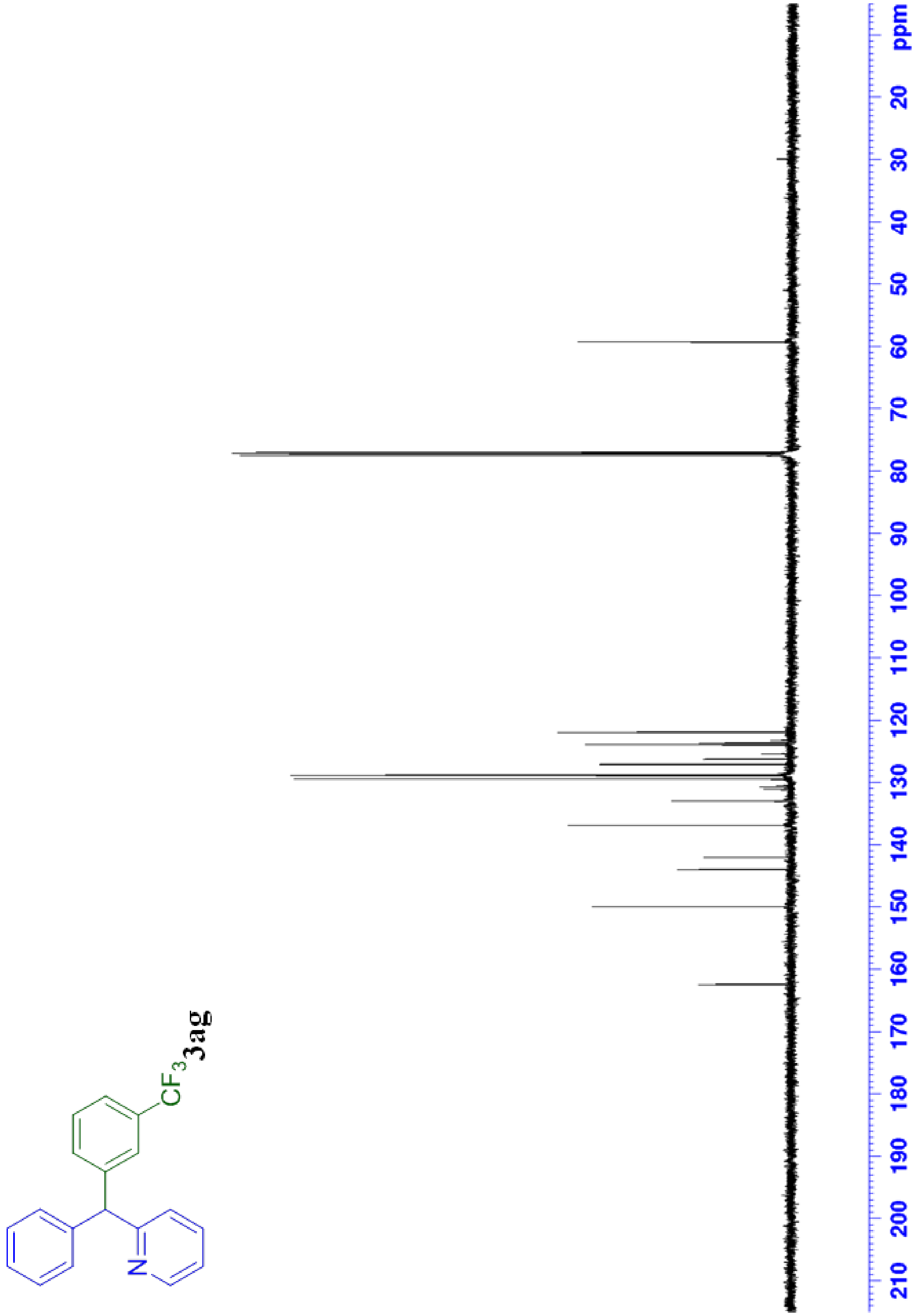

125 MHz $^{13}C\{^{1}H\}$ NMR of **3ag** in $CDCl_3$

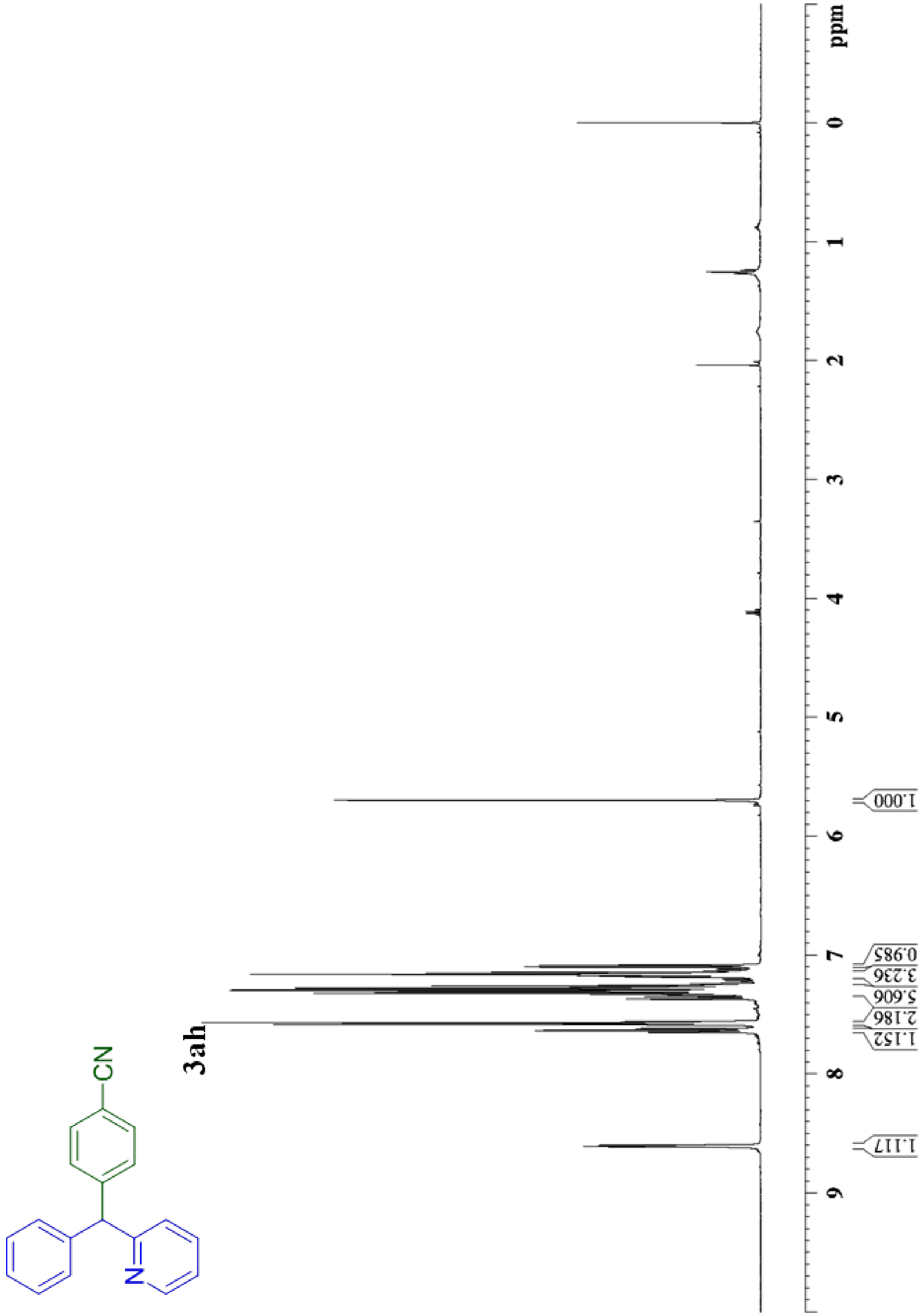

500 MHz ^{1}H NMR of **3ah** in $CDCl_3$

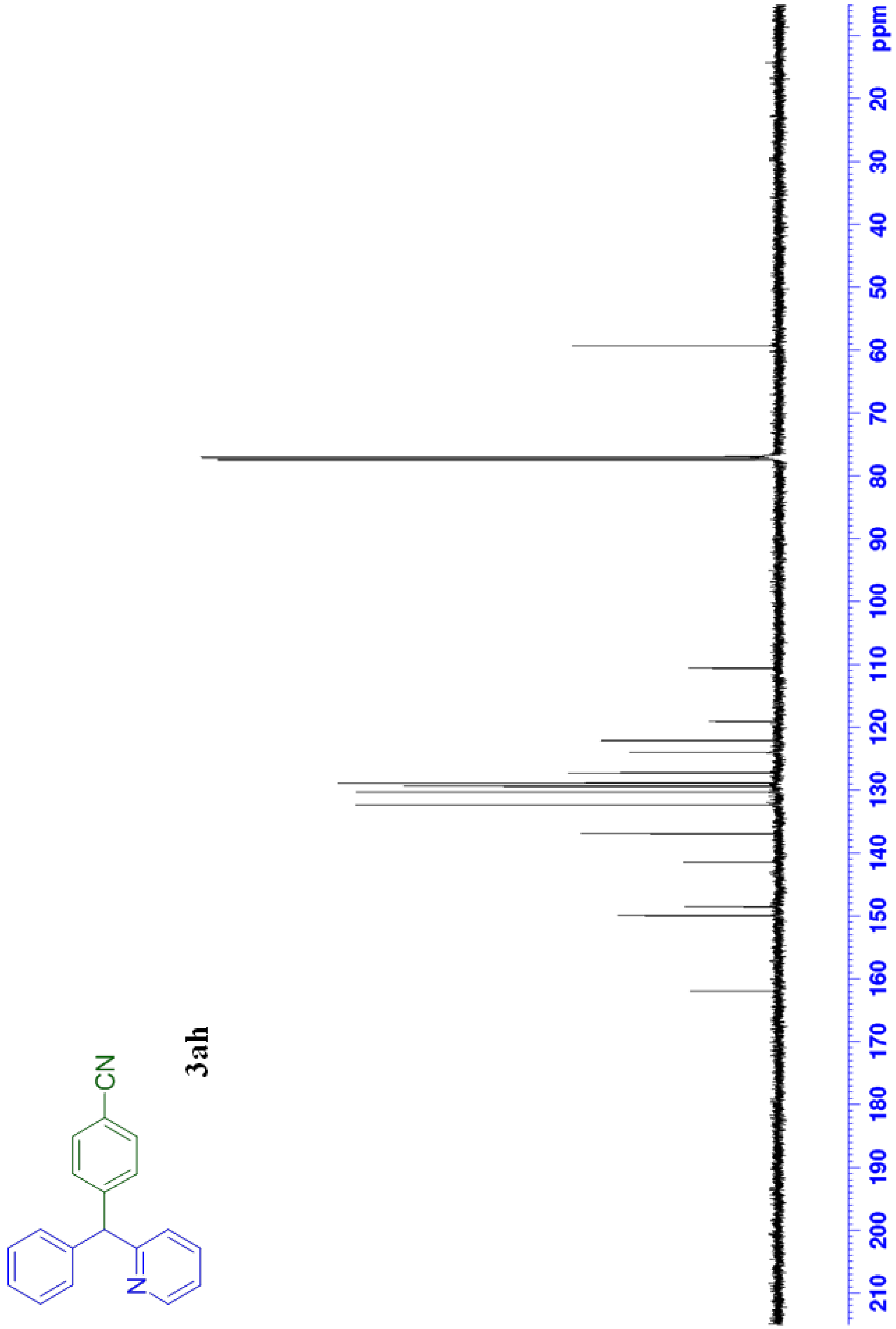

125 MHz $^{13}C\{^{1}H\}$ NMR of **3ah** in $CDCl_3$

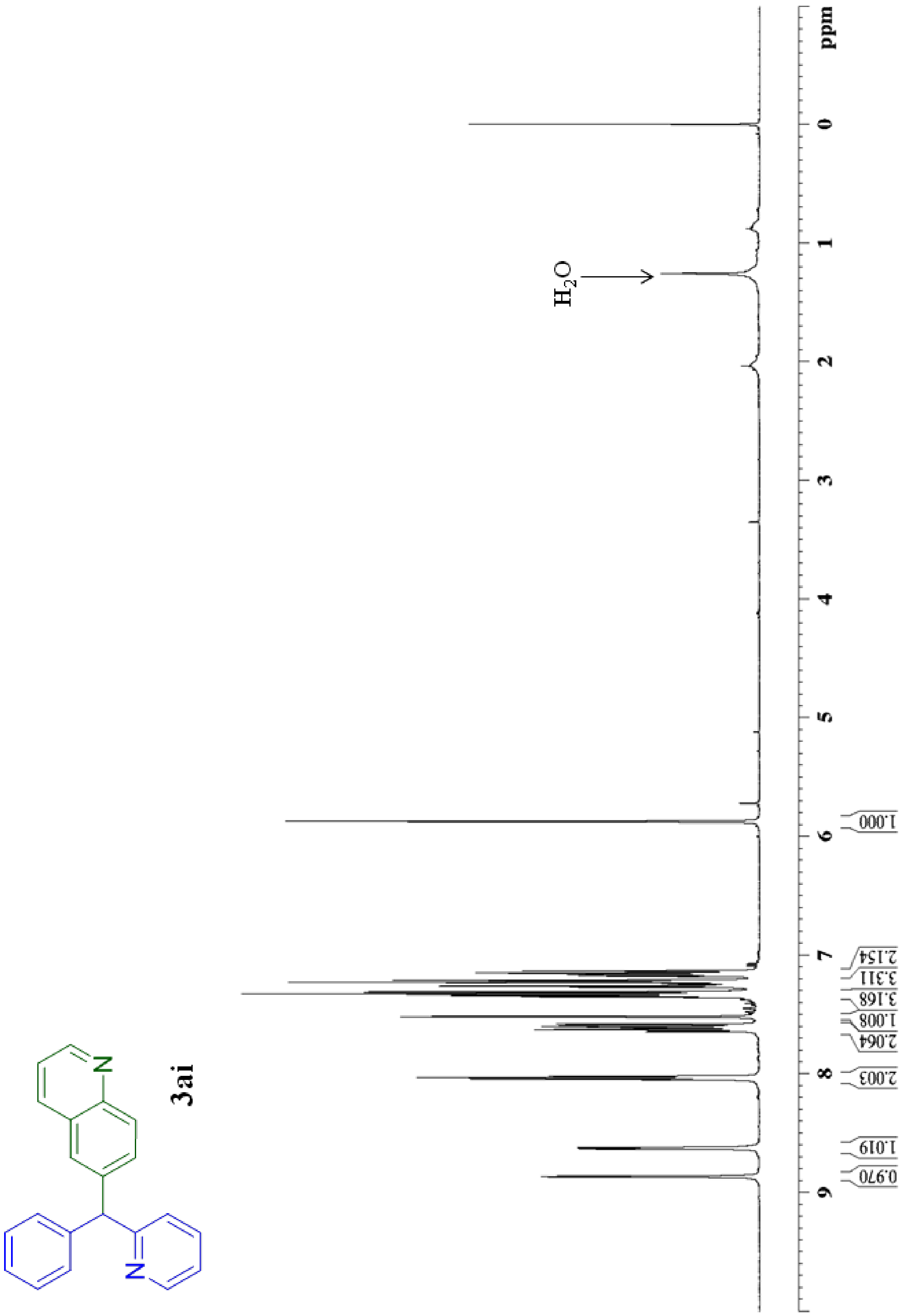

500 MHz ^{1}H NMR of **3ai** in $CDCl_3$

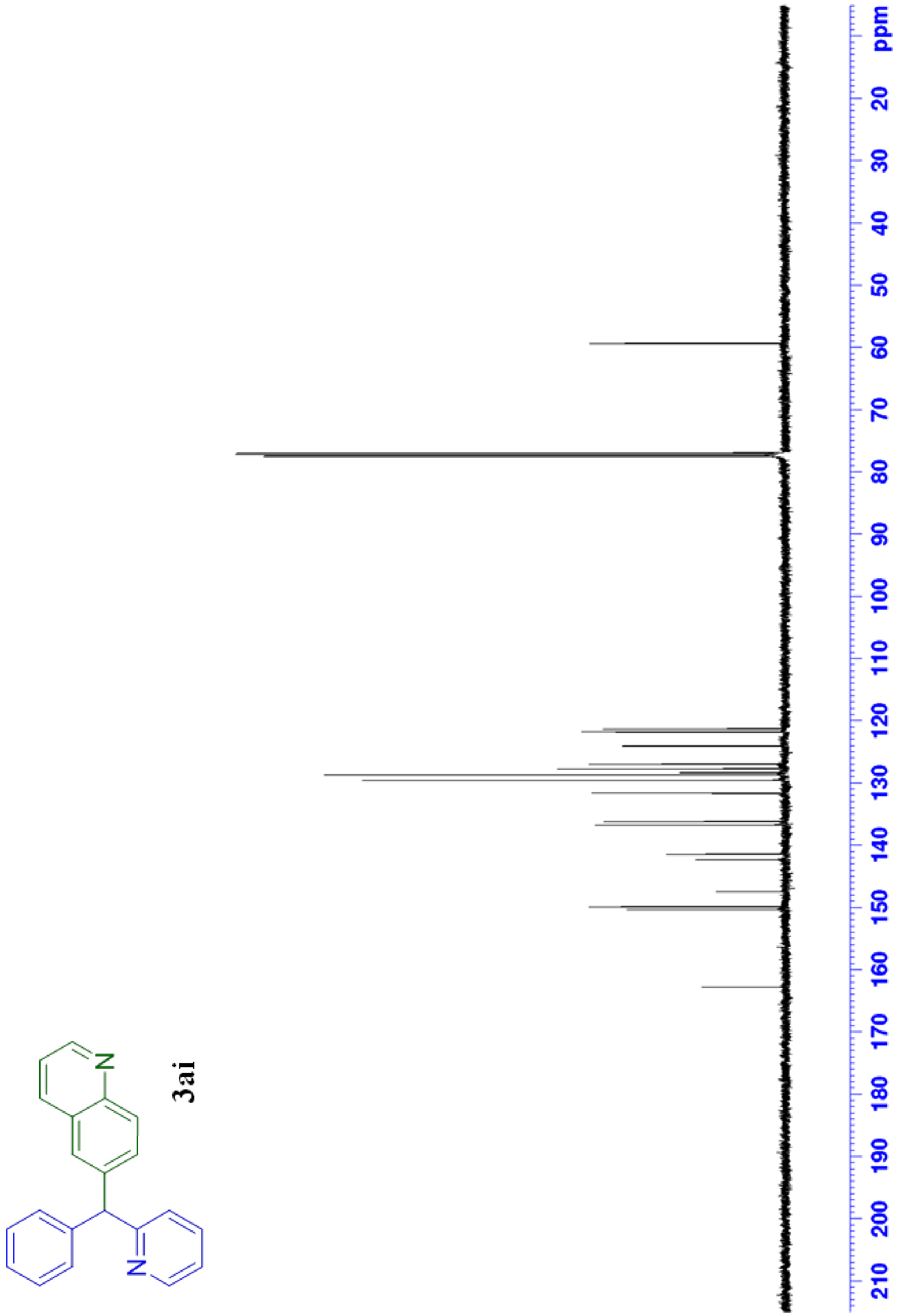

3ai

125 MHz $^{13}C\{^{1}H\}$ NMR of **3ai** in $CDCl_3$

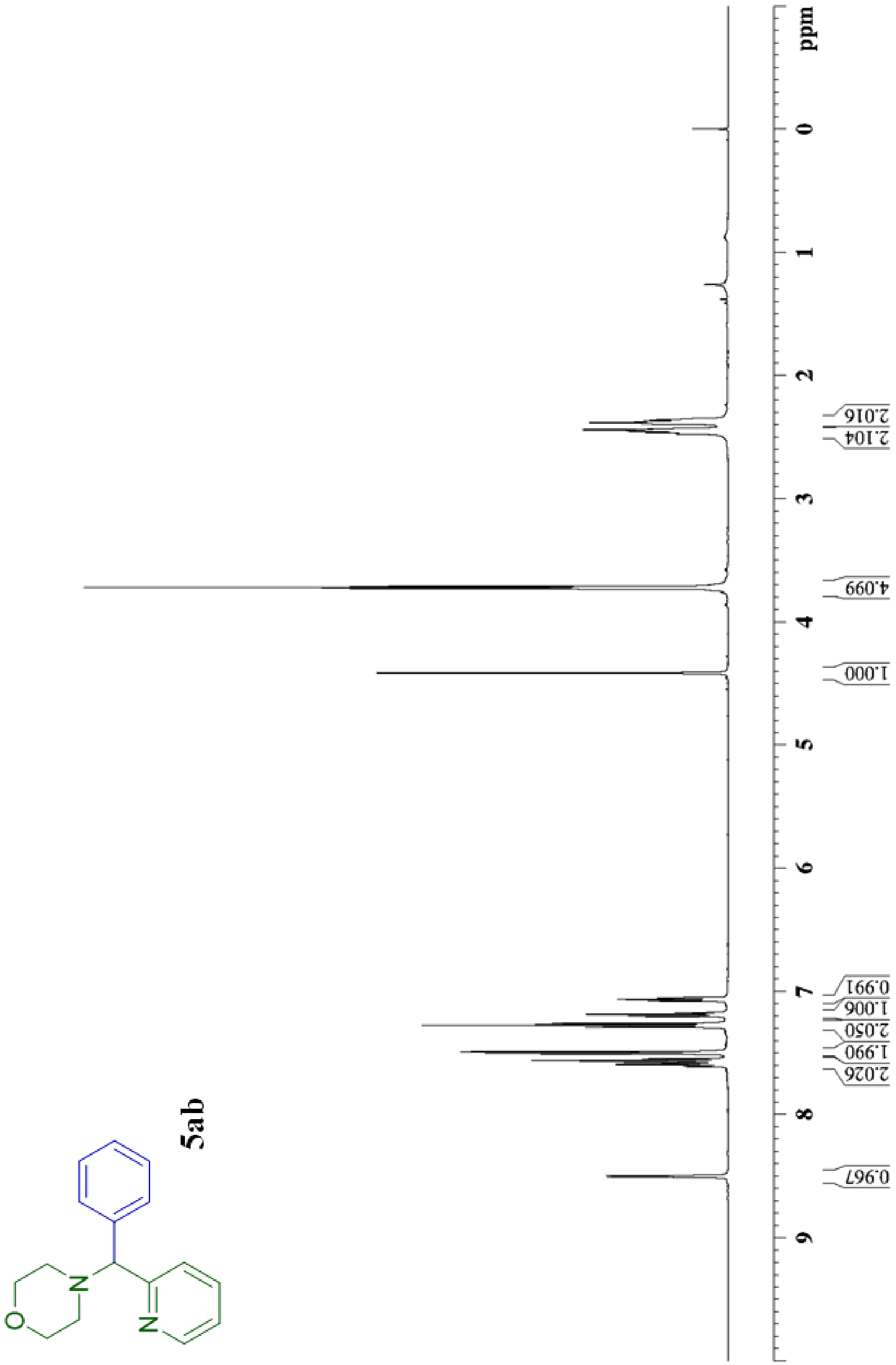

500 MHz ^{1}H NMR of **5ab** in $CDCl_3$

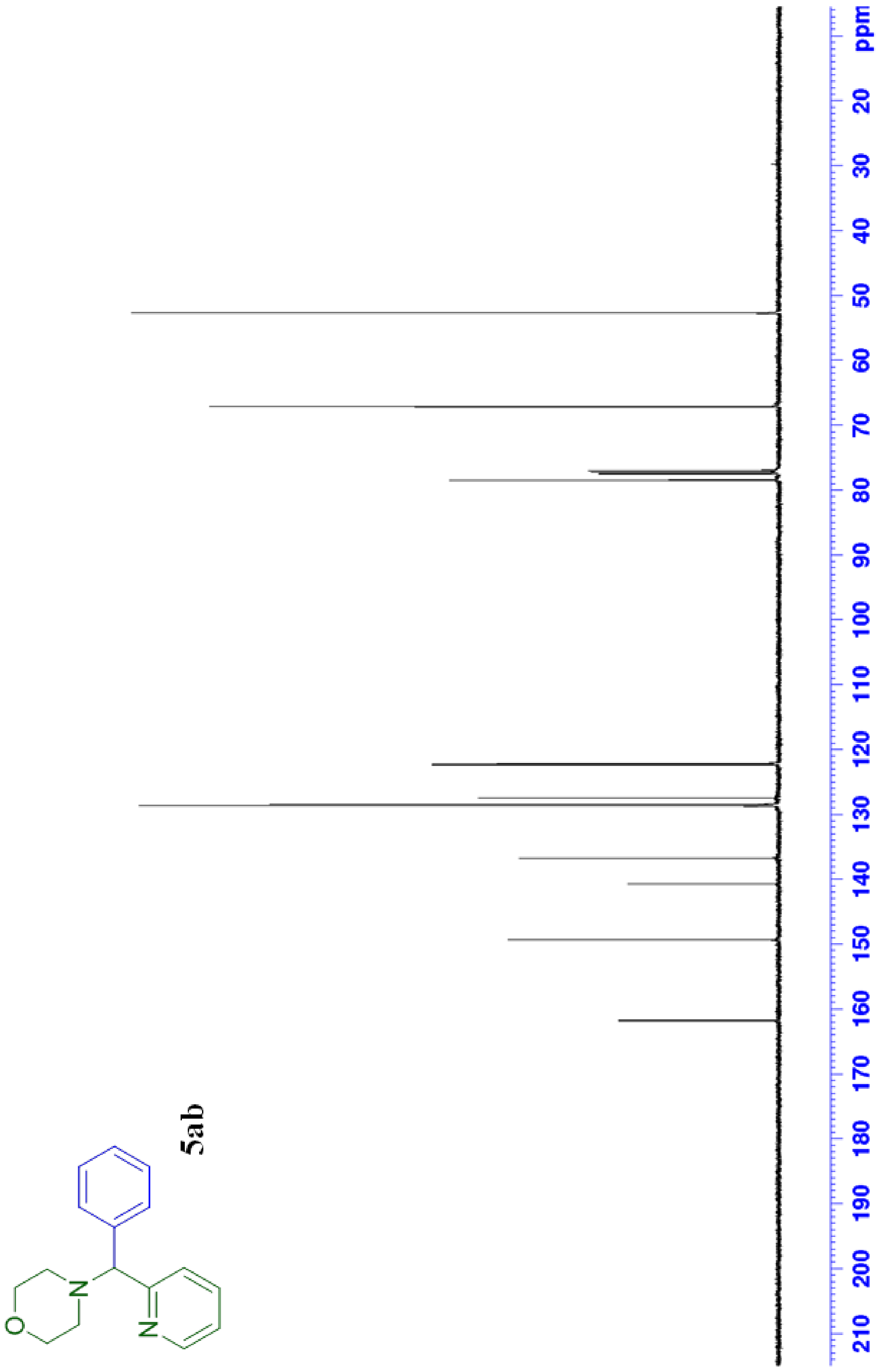

125 MHz $^{13}C\{^{1}H\}$ NMR of **5ab** in $CDCl_3$
